# Smart Contact Lenses as Wearable Ophthalmic Devices for Disease Monitoring and Health Management

**DOI:** 10.1021/acs.chemrev.3c00290

**Published:** 2023-09-25

**Authors:** Hunkyu Seo, Won Gi Chung, Yong Won Kwon, Sumin Kim, Yeon-Mi Hong, Wonjung Park, Enji Kim, Jakyoung Lee, Sanghoon Lee, Moohyun Kim, Kyeonghee Lim, Inhea Jeong, Hayoung Song, Jang-Ung Park

**Affiliations:** † Department of Materials Science and Engineering, Yonsei University, Seoul 03722, Republic of Korea; ‡ Department of Neurosurgery, Yonsei University College of Medicine, Seoul 03722, Republic of Korea; § Center for Nanomedicine, Institute for Basic Science (IBS), Yonsei University, Seoul 03722, Republic of Korea

## Abstract

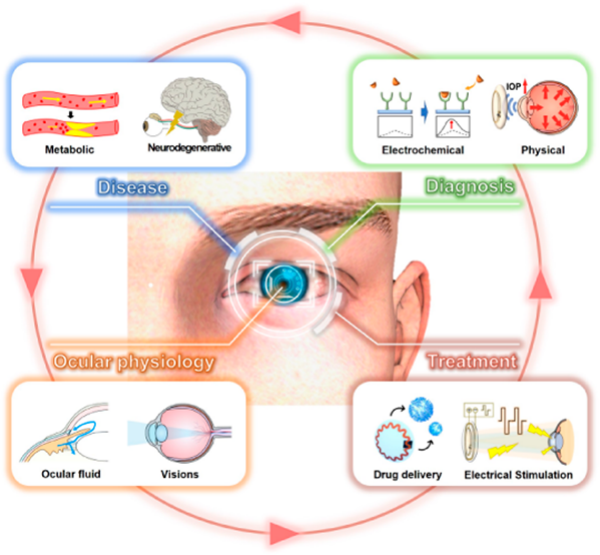

The eye contains a complex network of physiological information and biomarkers for monitoring disease and managing health, and ocular devices can be used to effectively perform point-of-care diagnosis and disease management. This comprehensive review describes the target biomarkers and various diseases, including ophthalmic diseases, metabolic diseases, and neurological diseases, based on the physiological and anatomical background of the eye. This review also includes the recent technologies utilized in eye-wearable medical devices and the latest trends in wearable ophthalmic devices, specifically smart contact lenses for the purpose of disease management. After introducing other ocular devices such as the retinal prosthesis, we further discuss the current challenges and potential possibilities of smart contact lenses.

## 1 Introduction

As people’s living standards have risen and their quality of life has improved, the desire for personalized healthcare has increased significantly. However, despite this demand, accurate diagnoses and treatments of diseases currently are provided only in hospitals. The limited access to healthcare for patients can cause inconvenience, resulting in time-consuming and costly diagnosis and treatment. Moreover, the limited and fragmented nature of the information provided during hospital visits can restrict the amount of information that the doctor can obtain, potentially affecting the accuracy of various diagnoses. As a result, there is a strong demand for wearable medical devices that are customized to the individual, convenient, and capable of obtaining real-time information related to the health of the human body.^1−6^

Recently, wearable medical devices for health management have been developed that can be compatible with various body parts.^7−10^ With the increasing demand for personalized health care and point-of-care diagnostics, the development of wearable medical devices for quick and precise diagnoses is essential.^11−13^ As an example, biosensors can be used to detect and monitor the biological and chemical molecules that exist in the human body in various forms.^14−17^ Chemical biomarkers, such as metabolites, ions, and proteins, can be found in various fluids in the body, such as blood, interstitial fluid, tears, and sweat.^18,19^ In addition, physical factors, such as temperature, heart rate, and pressure, also can act as biomarkers.^20−23^ Electrophysiological signals also can be identified along the nerve cells in organs, such as the brain, heart, and muscles, that are associated with various cranial nervous systems.^24−30^ Among them, the eyes are especially important because they provide both chemical and physical biomarkers related to specific point-of-care diagnosis and personalized treatment. For example, the ocular fluids, including tears, the aqueous humor (AH), and the vitreous humor (VH), contain a diverse range of chemical biomarkers derived from blood and ocular tissue, and physical biomarkers, such as intraocular pressure (IOP) and temperature, can be acquired as well as electrophysiological signals, such as electroretinogram (ERG).^31−34^

The smart contact lens (SCL) is a wearable ophthalmic device that can provide additional functions beyond vision correction by the integration of devices with the already popularized platform for the eye, soft contact lens. The integration of electronic components, such as sensors, microprocessors, and components of wireless communication, can be embedded inside SCLs. These electronic components allow SCLs to perform the function of measuring various biometric data, such as the glucose in tears, IOP, and other biomarkers for various diseases. Also, the SCLs can treat eye diseases through the delivery of drugs, heat, light, and electrical stimulation. Moreover, multifunctional SCLs capable of simultaneously diagnosing and treating diseases also are developed as personalized wearable platforms. The current SCLs face challenges regarding the accuracy and reliability of data due to limitations in sensing technology. Additionally, there are issues related to wireless communication, power delivery, wearer comfort, and stability. Although these challenges, the SCLs remain the most promising platform that can monitor and treat diseases in real time. As a wearable device applied to the eye for medical purposes, another representative platform is the retinal prosthesis.^35−37^ This device, also known as an artificial retina, is a type of medical device designed to restore vision for people with certain types of blindness. Retinal prostheses primarily are used to treat degenerative retinal diseases, such as retinitis pigmentosa (RP) and age-related macular degeneration (AMD), which damage the photoreceptor cells in the retina. These devices do not restore normal vision, but they can provide patients with some degree of visual perception, such as the ability to recognize shapes, detect movement, and navigate their environment. Although the retinal prosthesis is still in its early stages of development, it is one of the most innovative ocular devices in terms of being able to restore sight.

**Figure 1:**
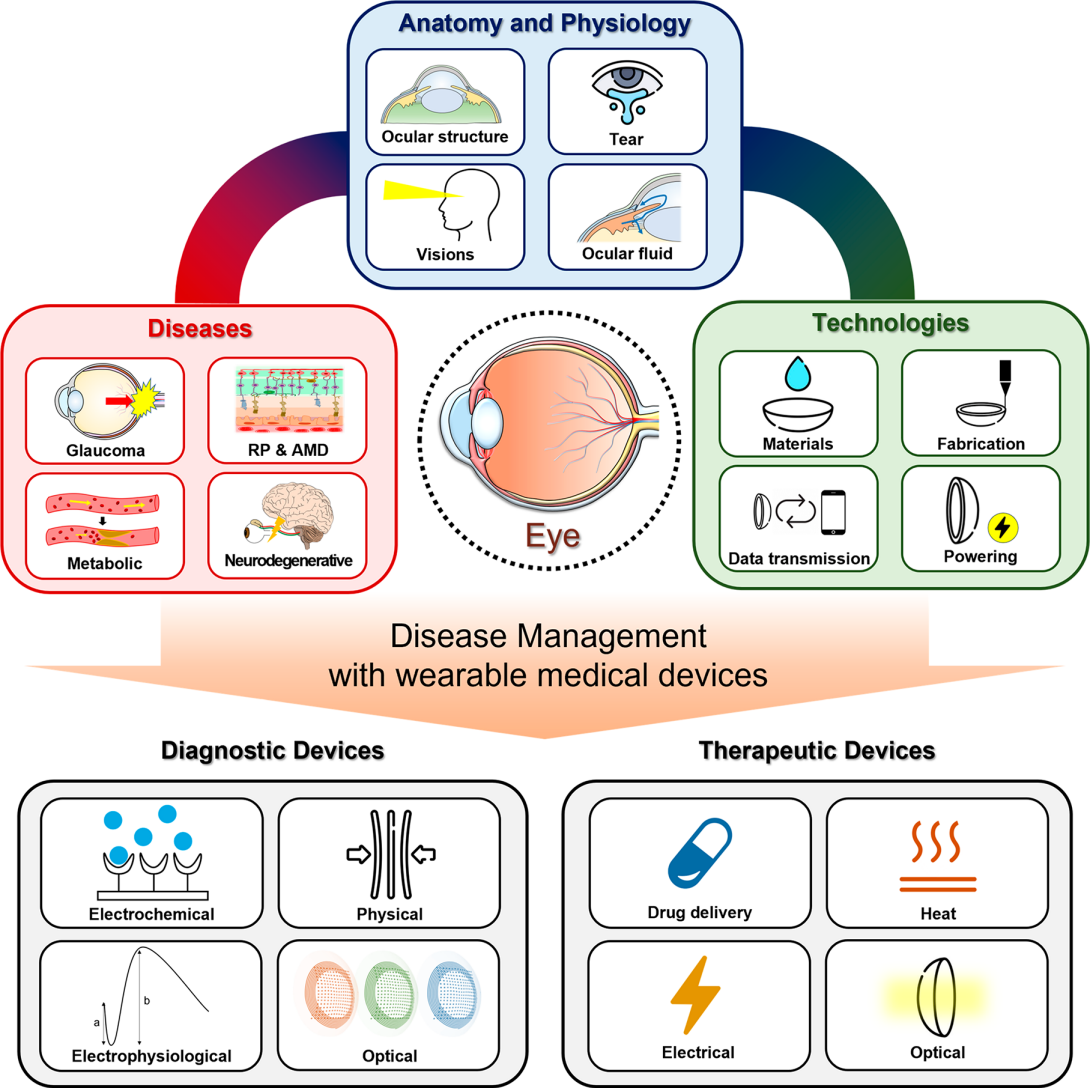
Overview of wearable ophthalmic devices for disease management.

In this overview, we will describe wearable eye devices designed for disease management (Figure 1), but first introduce the anatomy and physiology of the eye. Understanding the anatomy and physiology of the eye is necessary for their potential use in disease monitoring and treatment. Based on the anatomical and physiological characteristics of the eye, various biomarkers that can be obtained from the eye and their related diseases also will be described. Then, we will introduce the recent technologies required to fabricate state-of-the-art ocular devices. In addition, the current SCLs and retinal prostheses will be presented as representative platforms. Finally, we will summarize by discussing the challenges for ophthalmic devices, including SCLs, and by discussing their scalability and future direction.

## 2 Anatomy and Physiology of the Eye

The eye is a complex sensory organ that allows people to see objects in their immediate surroundings. Understanding the anatomy and physiology of the eye is essential for diagnosing and treating various diseases and disorders. The eye is composed of several interconnected structures that work together to capture, focus, and transmit visual information to the brain. The physiology of the eye includes the interaction of structures and their associated cells and tissues, as well as the complex mechanisms involved in the conversion of light into electrical signals that are transmitted to the brain. The eye contains body fluids, such as tears, AH, and VH, which help in maintaining visual function. These fluids contain various substances, such as glucose and cholesterol, which can act as biomarkers for specific diseases. In this part, we will explore the physiology of the eye in detail, including the anatomy and function of its various structures, and the mechanisms involved in vision.

### 2.1 Ocular Structure

**Figure 2:**
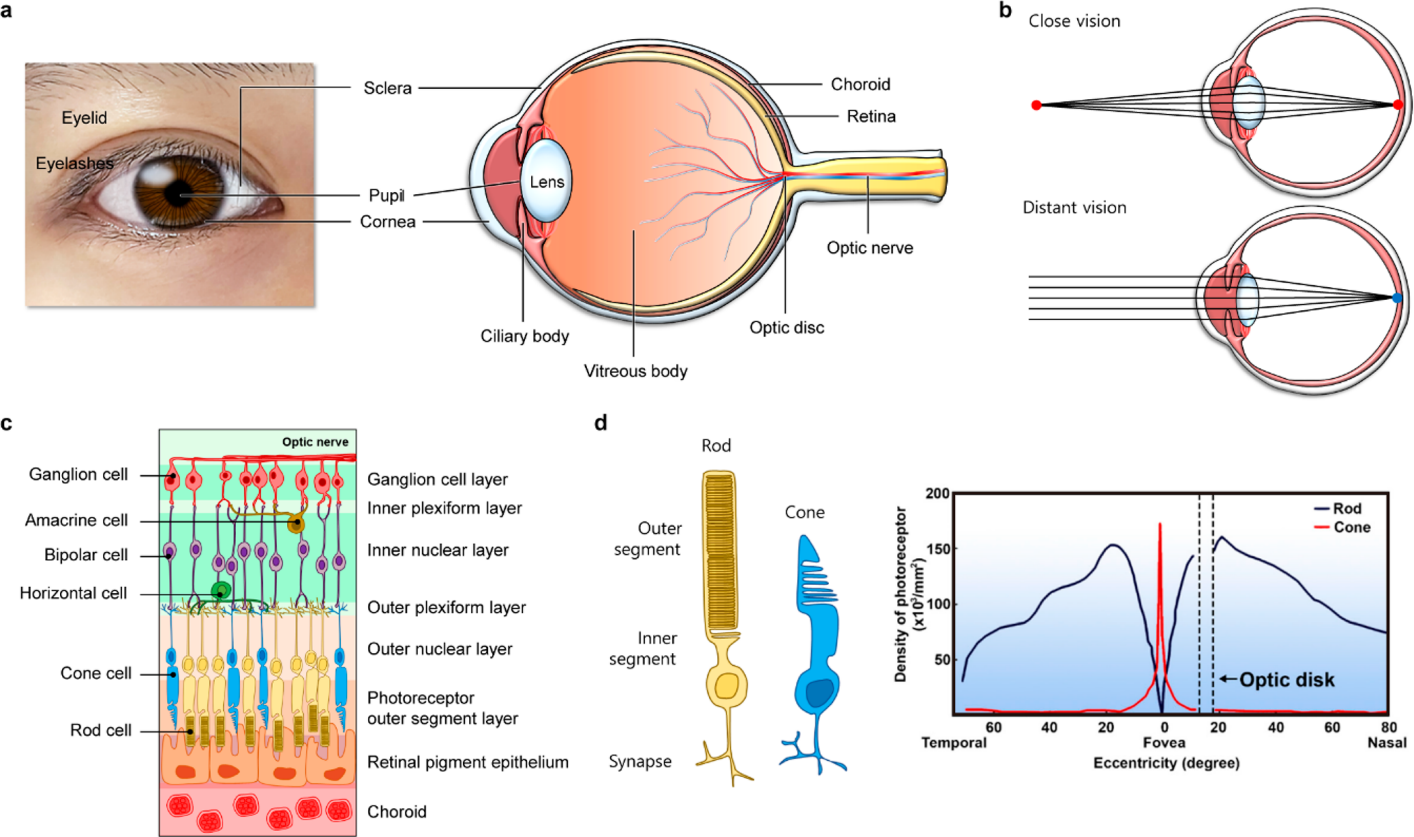
Ocular structure and vision process. (a) Photograph and schematic representation of the ocular structure. (b) The close (top) and distant vision (bottom). (c) Retinal structure and (d) a rod cell and a cone cell (left), and distribution of photoreceptors in the retina (right). Reproduced with permission from ref (38) Copyright 2021 Ptito et al. under CC BY 4.0 (https://creativecommons.org/licenses/by/4.0/).

Figure 2a illustrates the human eye and highlights its intraocular structure. The eyelid and eyelashes function as primary protection against harmful foreign substances. The cornea shields the front portion of the outer layer of the eyeball while the sclera covers the back of the eyeball. The sclera provides secondary protection for the internal components of the eye and maintains the shape of the eye. The choroid, which lies beneath the sclera, supplies oxygen and nutrients to the visual cells of the retina. It takes up the backside area of the middle layer of the eye wall. The front side of this layer is occupied by the iris and ciliary body. Also, the vitreous body is a gel-like substance composed of 99% water and 1% collagen, which makes up the majority of the volume of the eye.^38^ As these components of the eye exhibit different mechanical properties, it is important to consider these properties when designing a device directly interfacing them (Table 1).

**Table 1:** Mechanical Properties of Eye Components

Components	Cornea	Sclera	Choroid	Vitreous humor	Retina
Elastic modulus	0.1–1.5 MPa	1–20 MPa	100 kPa	<1 kPa	<1 kPa

At the back of the eye, the optic nerve is connected directly to the central nervous system (CNS). The optic nerves extend from the optic disk, which is the point at where no visual cells are present. In addition to these components, various other components, such as the lacrimal and meibomian glands, play an important role in the physiology of the eye. In the following section, we will discuss these components of the eye in detail because they are extensively involved in the vision process and the overall physiology of the eye.^39^

### 2.2 Vision

#### 2.2.1 Components of the Vision Process

The sense of vision, regarded as one of the primary senses, is initiated when light stimulates the eye. When light enters the eye and passes through the various ocular components, it eventually reaches the retina at the back of the eye, allowing us to perceive visual information. Vertebrates, including humans, process visual information by utilizing the various components of the eye to protect their bodies and respond appropriately to different situations.^40^

The first components of the eye that encounter incoming light are the cornea and the pupil. The iris determines the amount of light that enters the eye by regulating the size of the pupil. For instance, in bright conditions, the muscles of the iris contract to reduce the size of the pupil, but in dark conditions, they enlarge the pupil by expanding the muscles of the iris. As the light passes through the pupil, the lens adjusts its thickness and refracted the light based on the distance to the object. When viewing a nearby object, the ciliary muscle, which is connected to the lens, contracts, causing the lens to thicken and refract the light at a higher refractive index (Figure 2b, top). It projects a clear image of the nearby object onto the retina. Conversely, to obtain visual information from a distant object, the ciliary muscle relaxes, making the lens thinner (Figure 2b, bottom). As a result, the lens refracts light at a lower refractive index, allowing us to accurately perceive objects at a distance.^41^

#### 2.2.2 Retina Structure

The retina contains photosensitive visual cells, and it is the most essential component for the vision process. It is a thin, multilayered membrane structure that covers the inner layer of the eye wall. Figure 2c shows each layer of the retina structure, which includes five types of retinal neurons, i.e., photoreceptor cells (cones and rods), horizontal cells, bipolar cells, amacrine cells, and ganglion cells.^42^

The retinal pigment epithelium (RPE) layer is adjacent to the choroid that provides nourishment to the visual cells via abundant blood vessels. Initially, visual information is received by the photoreceptor’s outer segment (OS), which contains photosensitive molecules. Then, this information is transmitted progressively to each layer of the retina, reaching in turn the CNS through the optic nerve.

The outer nuclear layer (ONL) contains the cell bodies of photoreceptors whose axonal branches stretch to the outer plexiform layer (OPL), where they communicate with bipolar cells and horizontal cells forming synapses. Similarly, the cell bodies of bipolar cells, horizontal cells, and amacrine cells are located in the inner nuclear layer (INL). The amacrine cells in this layer regulate the transfer of signals between the retinal ganglion cells (RGCs) and the bipolar cells. The bipolar cells form synapses with RGCs and amacrine cells in the inner plexiform layer (IPL) to transmit visual information from the photoreceptors. Finally, the RGCs are connected to the optic nerve, which serves as the messenger that transmits visual information to the CNS.

#### 2.2.3 Photoreceptors

The photoreceptors convert incoming light into an electrical signal to communicate with the nervous system. There are two main types of photoreceptors, i.e., rods and cones. As their names imply, the rod cell has an elongated and slender outer segment, while the cone cell has a short and tapered segment (Figure 2d, left). There are approximately 20 times more rod cells than cone cells in the entire retinal area, however, the fovea, the central region of the retina, has a higher concentration of cone cells (Figure 2d, right). Thereby, cone cells are responsible for high-resolution vision, and they function best in bright light. In contrast, rod cells are more sensitive to low-light conditions and exhibit a low accuracy of visual perception.

The photoreceptors fire with the signal of graded potential, which can be either excitatory or inhibitory. In addition, unlike other sensory receptors that experience depolarization against the stimulation, photoreceptors undergo hyperpolarization when they are stimulated by light. This phenomenon is due to the cascade of phototransduction, which involves the activation of ion channels. The outer segment of the photoreceptor consists of stacked membrane discs. Each membrane contains a cation channel that remains open in dark conditions. The cation channel, combined with cyclic guanosine monophosphate (cGMP), allows the influx of sodium and calcium ions, which bring about an increase in membrane potential and cell depolarization. As a result, the photoreceptor releases a neurotransmitter, i.e., glutamate, to the bipolar cell of the postsynaptic neuron.^43^

When the eye is exposed to the light, the phototransduction process starts with the conformational modification of opsin, which is a photosensitive protein that composes the membrane disc. Specifically, rhodopsin, a type of opsin present in rod cells, is a combination of the opsin protein and retinal molecules.^44^ The retinal molecules are isomeric compounds derived from vitamin A that exist in two different isomeric forms, i.e., 11-cis retinal and all-trans-retinal. When 11-cis retinal is stimulated by light, it experiences a conformational inversion to all-trans-retinal. This transition, in turn, triggers the decomposition of cGMP to GMP, and the resulting decrease in the concentration of cGMP elicits the closure of the cation channel in the membrane disc. Subsequently, the cation influx is diminished, causing the hyperpolarization of the photoreceptor and eventually suppressing the release of glutamate.^45,46^

This process demonstrates that photoreceptors respond to the intensity of the light. However, it does not explain how we distinguish the color of an object. Cone cells are responsible for color vision, as they contain three types of opsins that have different spectral sensitivities, while the rhodopsin of the rod cells detects all ranges of visible wavelengths. The three opsins in the cone cells are named S-opsin, M-opsin, and L-opsin according to the wavelength of their detection range, and they show maximal sensitivity to wavelengths of 445, 535, and 575 nm, respectively. The different combinations of the responses of the three opsins allow us to perceive various colors.^47^

#### 2.2.4 Signal Processing

Once the electrical signal is generated by photoreceptors, it is transmitted sequentially to the bipolar cell, the ganglion cell, and the optic nerve. Those visual cells conduct specific communication to process the graded potential from the photoreceptors. In particular, bipolar cells play a critical role in integrating and processing visual information before transmitting it to the brain.

There are two types of bipolar cells, i.e., ON-bipolar cells and OFF-bipolar cells, which respond differently to changes in the intensity of light. These cells are either depolarized or hyperpolarized depending on the type of glutamate receptor. The OFF-bipolar cell expresses an ionotropic glutamate receptor named α-amino-3-hydroxy-5-methyl-4-isoxazolepropionic acid (AMPA), which opens a transmembrane channel allowing the influx of cations when glutamate is released from the photoreceptor. Consequently, the OFF-bipolar cell is depolarized when decreasing in light intensity and hyperpolarized when increasing in light intensity. However, in the case of an ON-bipolar cell, the tendency for depolarization and hyperpolarization depends on the cascade of a metabotropic glutamate receptor known as mGluR6. When mGluR6 combines with glutamate, it triggers a series of processes that close the cation channel, resulting in the hyperpolarization of the cell.^48,49^

In conclusion, the two types of bipolar cells respond to light in opposite ways. The OFF-bipolar cell is activated by the transition from light to dark, while the ON-bipolar cell is activated by the transition from dark to light. Then they transmit the electrical signals to the off-ganglion cell and the on-ganglion cell, respectively. The RGCs encode the signals by sorting out the valuable information and then delivering the signals to the brain through the optic nerve.

### 2.3 Tear Film

The tear film is a complex and dynamic structure that plays a critical role in maintaining the health and function of the ocular surface. The tear film acts as a protective barrier, lubricant, and nutrient source for the cornea and conjunctiva. In addition, tear fluid contains several biomarkers that can provide information about a person’s health, including dry eye syndrome (DES), glaucoma, diabetes, and cardiovascular disease (CVD). Tears are relatively easy to collect as they always exist on the cornea, and they are not easily contaminated due to the protection of the eyelid. Based on these characteristics, researchers have become increasingly interested in exploring the potential use of tear fluid as a diagnostic tool. For example, SCLs are the most representative tear-based wearable devices which have the potential to provide noninvasive and continuous monitoring of various health parameters in tears.^31,50^ In order to effectively utilize tear-based devices, it is important to have a comprehensive understanding of the properties of the tear film.

#### 2.3.1 Tear Film Layer

**Figure 3:**
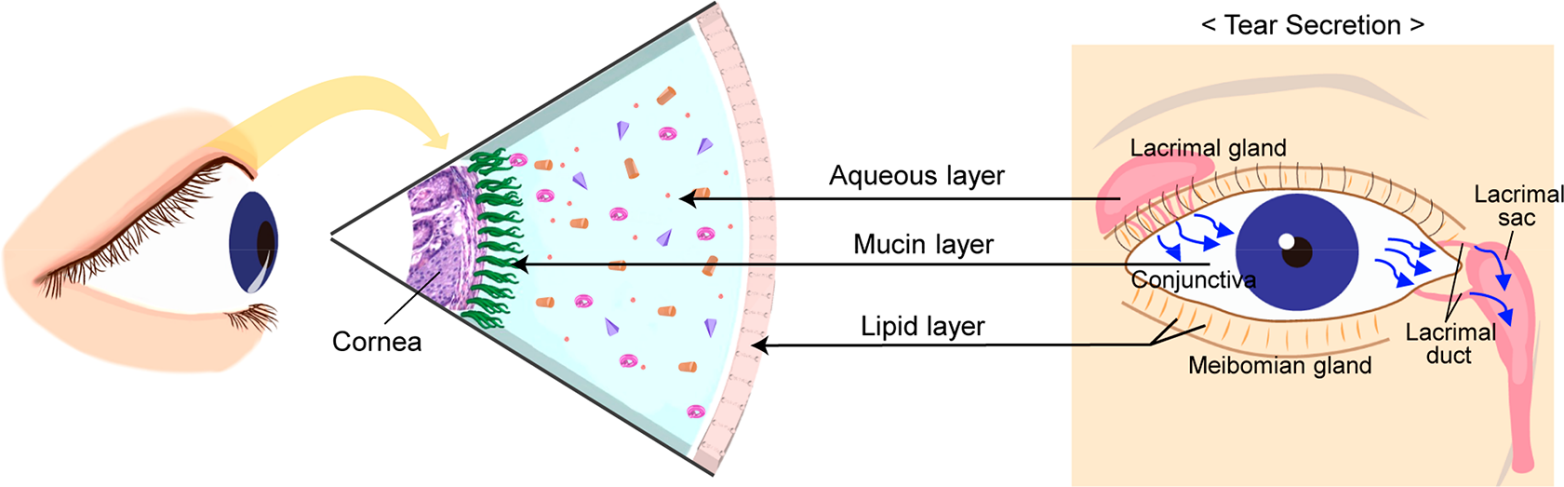
Schematic illustration of the three major tear film layers (left) and the tear secretion pathway (right): inner mucus layer, middle aqueous layer, and outer oil (lipid) layer.

The tear film is composed of the outermost lipid, the middle aqueous, and the inner mucin layers (Figure 3).^51,52^ The outermost layer of the tear film is the lipid layer, which mainly consists of fatty acids, cholesterol, and other lipids. The function of the lipid layer is a form a lipid barrier on the surface to help slow evaporation and keep the eye surface moist and hydrated.^53^ In addition, the lipid layer provides a physical barrier to protect the eyes from external environmental factors.^54^ It helps to prevent damage by introducing dust, debris, and other foreign particles into the eyes. The middle aqueous layer is the second layer, occupies most of the tear film, and nourishes the cornea and conjunctiva.^55^ The layer contains various growth factors and cytokines that promote the growth and repair of the cornea and conjunctiva. One of the main functions of the aqueous layer is to lubricate the surface of the eye. The inner mucin layer is the third and innermost layer of the tear film.^55^ The key role of the mucin layer is to secure the tear film to the surface of the eye and provide a smooth and even distribution of tears over the cornea. In addition to anchoring the tear film, the mucin layer also provides a barrier against harmful pathogens and bacteria, which are highly adhesive and can trap bacteria and other microorganisms, preventing them from penetrating the ocular surface.

#### 2.3.2 Secretion and Circulation of the Tear

The secretion of each tear film layer mentioned above occurs in different parts of the eye, which together form a multilayered tear film structure. The lipid layer is secreted by the meibomian glands, which are modified sebaceous glands located at the edge of the eyelids.^56^

Meibum, the oily substance secreted by the meibomian glands in the eyelids, is a complex mixture of lipids that includes both polar and nonpolar components, and it can remain in a liquid state within the tear film due to its low melting point.^56−58^ The secreted meibum is spread across the surface of the tear film by the blinking of the eyelids. The meibomian glands are innervated by the autonomic nervous system, and they contain several neuropeptides. However, there is currently no direct evidence to support the idea that either the sympathetic nervous system or the parasympathetic nervous system controls the secretion of meibum.^59^ The aqueous layer is produced by the lacrimal glands, which are located above the outer corner of each eye. This gland is composed of small tubes and acinar structures that release tears through tiny ducts into the conjunctival fornices.^55^ The lacrimal gland can be influenced by different factors, including hormonal and neural factors, such as the parasympathetic and sympathetic nervous systems.^60,61^ When the cornea, conjunctiva, optic nerve, or brain are stimulated, they can trigger the secretion of fluid from the lacrimal gland via both parasympathetic and sympathetic efferent pathways.^62^ The secretion of tears can be increased by parasympathetic neuropeptides, which stimulate the lacrimal gland, and by sympathetic stimulation, which affects the vascular supply to the gland.^63^ The mucin layer primarily is secreted by the conjunctival goblet cells, although both the corneal and conjunctival epithelium have roles in its secretion. Goblet cells found in the conjunctiva are a type of secretory apocrine cells. When stimulated, these cells release mucin and glycoproteins by having vesicles located below the cell surface fuse with its outer membrane.^64^ Mucin secretion by goblet cells can be triggered by various stimuli, including histamine, antigens, immune complexes, and mechanical actions, such as blinking.^65^ In addition, both direct and indirect neural controls of mucin secretion have been observed.^66^ Goblet cells located in the conjunctiva are innervated by sensory, sympathetic, and parasympathetic nerves.^67^ As a result, the cornea and conjunctiva can indirectly stimulate mucus secretion from goblet cells by diffusing neuropeptides from these neighboring nerves.^66,68^

Tear films constantly are being produced and circulated over the ocular surface. In detail, tears secreted by the lacrimal gland are distributed to the ocular surface through blinking, and the tear film is drained eventually through the lacrimal duct, which is a small channel that connects the eyes to the nasal cavity.^69,70^ From there, the tear film is eliminated from the ocular surface through the nose or swallowed into the digestive system. Tear clearance also occurs through evaporation. Tear evaporation is influenced by various factors, including humidity, temperature, airflow, and the composition of the tear film.^71^ As mentioned previously, the lipid layer of the tear film is essential in minimizing tear evaporation from the surface of the eye. Since the secretion and distribution of the lipid layer occur due to the blinking of the eye, prolonged periods without blinking can lead to rapid evaporation of the tear film, resulting in a decrease in tear film breakup time^72^ and an increase in tear osmolarity. The increase in tear osmolarity is thought to contribute to the development of various dry eye conditions. Therefore, continuous secretion and circulation of the tear film with proper eye blinking are crucial for preserving the health and optimal functioning of the ocular surface.

The properties of the cornea also affect tear circulation. As mentioned above, the tear film serves as both a physical and chemical barrier to protect the cornea, while also facilitating material exchange with the cornea. When drugs are instilled externally or tears are secreted from the lacrimal gland, various components are exchanged with the ocular tissue through the cornea. Depending on the characteristics of the cornea, the change in tear composition may appear differently, and in order to accurately measure the tear composition, it is necessary to clearly understand the relationship between the cornea and tears.

#### 2.3.3 Three Types of Tears

There are three main types of tears (i.e., basal, reflex, and emotional tears).^73,74^ These types of tears are produced for different reasons and serve different functions. Basal tears are literally tears that always exist to maintain eye function. However, reflex tears, are produced in response to eye irritants. These irritants can stimulate nerve endings in the cornea and the conjunctiva, which send signals to the brain to produce tears. Reflex tears are produced quickly and in large amounts to flush out the irritant and protect the eye from further damage. The basal tear flow rate is 1.2 mL min^–1^ but can be as much as 100 times faster for reflex tearing. Emotional tears are produced in response to various emotions, e.g., sadness, joy, or stress. Although not fully understood, the production of emotional tears is believed to involve the limbic system, which plays a crucial role in regulating emotions. Reflex tears and emotional tears are added to basal tears due to a specific reaction, and their composition is different from that of basal tears. Stuchell et al. reported that the levels of lysozyme and lactoferrin were higher in reflex tears than in basal tears.^75^ Also, in comparison to basal tears, emotional tears have been found to contain increased concentrations of stress hormones, such as cortisol and adrenaline, as well as higher levels of the neurotransmitter leucine enkephalin.^76^

Therefore, in order to obtain accurate information about specific biomarkers in the body through tears, measurements in basal tears, excluding the effects of reflex tears and emotional tears, should be performed. However, obtaining accurate measurements of basal tears is challenging. The mechanical stimulation during the tear collection process can induce the secretion of reflex tears, which changes the concentrations of the biomarkers in tears.^77^ The reason certain blood components are reflected in tears is still unclear. Until now, the most dominant explanation for this is a phenomenon called “plasma leakage”. Plasma leakage refers to the process by which small amounts of certain components of blood pass through the blood-tear barrier and enter the tear fluid.^78−80^ Due to this phenomenon, metabolites, ions, proteins, etc., in the blood can exist in specific concentrations in basal tears (Table 2).^50,81−88^ If there is an apparent correlation between specific biomarkers in both fluids, tear fluid may serve as a substitute for blood composition analysis. Therefore, these points suggest that, in order for tear-based devices to be used clinically, it is necessary to measure the biomarkers in tears without eye irritation and reveal the correlation between biomarkers in tears and blood.

**Table 2:** Comparison of Components in Basal Tears and Blood

Analyte	Tear fluid concentration	Blood concentration	Reference
Glucose	0.1–0.6 mmol L^–1^	3.3–6.5 mmol L^–1^	(50, 81)
Lactate	2–5 mmol L^–1^	0.5–0.8 mmol L^–1^	(81, 82)
Total cholesterol	0.2–1.9 mmol L^–1^	4–8 mmol L^–1^	(83)
Ascorbic acid	0.22–1.31 mmol L^–1^	0.04–0.06 mmol L^–1^	(50, 84)
Uric acid	0.025–0.15 mmol L^–1^	0.09–0.2 mmol L^–1^	(85, 86)
Na^+^	120–165 mmol L^–1^	140 mmol L^–1^	(50, 81, 82)
K^+^	20–42 mmol L^–1^	4.5 mmol L^–1^	(81, 82)
Cl^–^	118–135 mmol L^–1^	100 mmol L^–1^	(81, 82)
Mg^2+^	0.5–0.9 mmol L^–1^	0.9 mmol L^–1^	(81, 82)
Ca^2+^	0.4–1.1 mmol L^–1^	2.5 mmol L^–1^	(81, 82)
Total protein	5–11 mmol L^–1^	65–83 g L^–1^	(87, 88)

Also, tear composition can vary not only depending on the presence or progression of the disease but also due to a variety of factors not related to the disease. Individual variations in tear composition arise from factors such as age, external environment, emotional and physical stimuli, and genetic factors. When developing diagnostic SCLs for diseases, it is crucial to consider and address these diverse aspects in the design.

### 2.4 Aqueous Humor and Vitreous Humor

The interior of the eye is divided into two main chambers centered on the lens. Both of these chambers are filled with fluid, and they are referred to as the AH and the VH, respectively. AH and VH are two types of fluids that are different from tears and that play important roles in maintaining the structure and function of the eye. Like tears, these two types of fluids contain various biomarkers that can convey information about various diseases, such as diabetes and glaucoma. Although relatively more difficult to access than tears, these two types of fluids also have the potential to be used to diagnose diseases.

#### 2.4.1 Functions of Aqueous Humor and Vitreous Humor

AH and VH are important fluids in the eyes. AH is a clear and watery fluid that is located in the anterior chamber of the eye, between the cornea and the lens. The VH is a transparent, colorless, and gel-like fluid in the vitreous chamber of the eye, behind the lens. AH and VH are essential components of the eye, and they contribute to maintaining the health and functioning of the eyes.

The most important role of AH and VH is regulating the pressure inside the eye, known as IOP. IOP refers to the constant pressure that is maintained inside the eyeball. IOP is generated by the flow of AH, and it is important with respect to the progression of glaucoma, which will be discussed later. Also, maintaining a constant IOP in both the AH and the VH is a critical factor in maintaining the shape of the eyeball. The most basic function of the eyes is to transmit visual information to the brain. For light entering the eye to reach the retina without loss, the AH and VH must be clear, and, at the same time, the eyeball must maintain its normal shape.

AH and VH also supply nutrients to the components of the eye. They are secreted into the chamber through blood vessels, and they contain various nutrients. There are blood vessels in the eye, but there are also parts where there is no blood. Therefore, the AH and the VH deliver nutrients, such as glucose and ascorbic acid, to the tissue of the entire eye, including the cornea, the crystalline lenses, and the retina.

Conversely, the AH and VH also serve to remove the waste products that are generated through the metabolism of the ocular tissue. Various waste products produced in the ocular tissue are discharged into the AH and VH and are transferred into the blood. Thus, due to the unique characteristics of the eye that are responsible for vision, the AH and VH act as blood vessels in areas that otherwise could not be reached.

#### 2.4.2 Generations and Outflow

**Figure 4:**
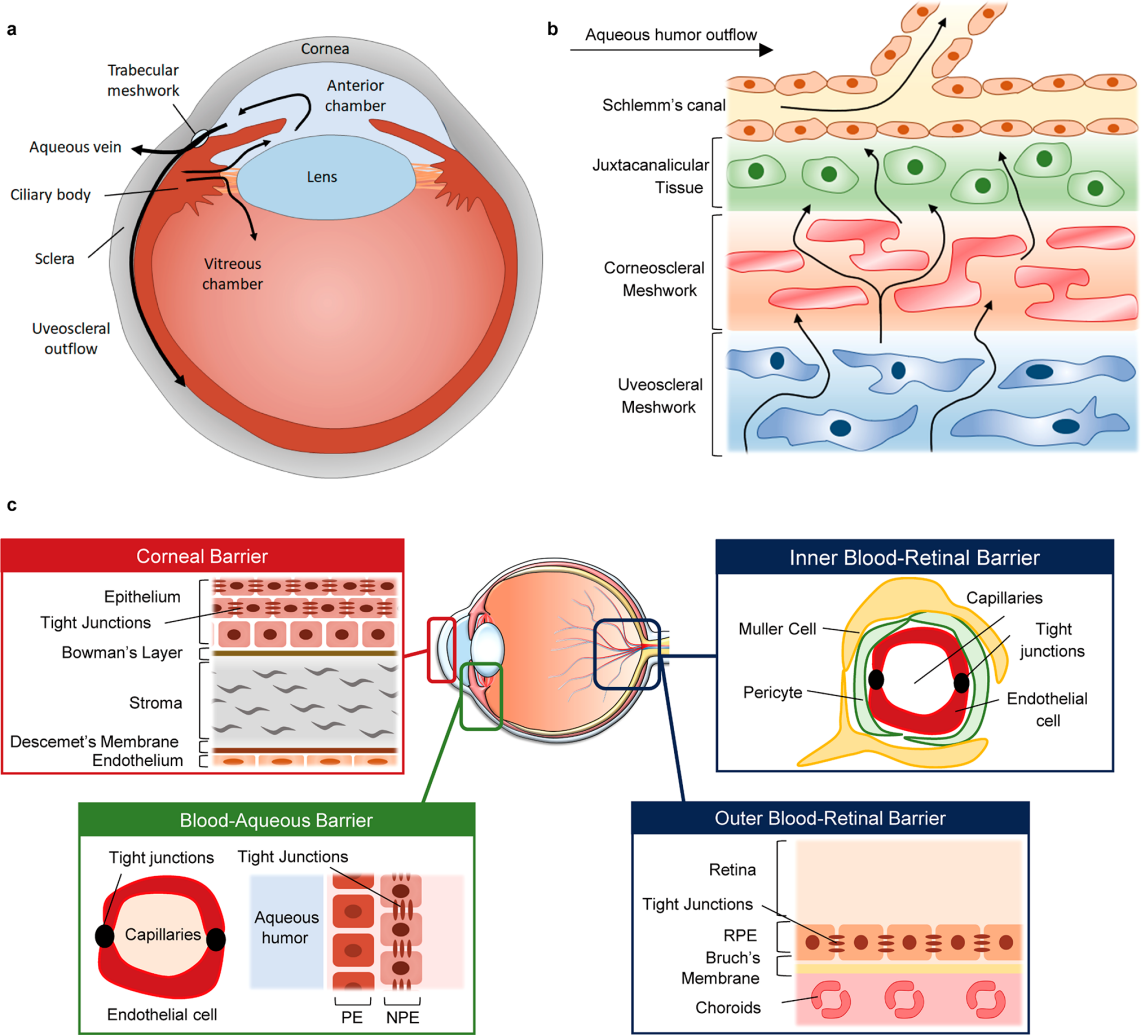
Dynamics of AH and VH. (a) Anatomical features of the human eye and production of eye fluid. (b) Schematic of the flow of aqueous humor from the ciliary body through the anterior angle. (c) Corneal barrier, and two main blood-ocular barriers are proposed: the blood-aqueous barrier (BAB) and the blood-retinal barrier (BRB).

The human eye is filled with two fluid-like substances, i.e., AH and VH, that maintain the IOP and shape of the eye. AH is a water-like liquid in front of the lens, and VH is a gel-like substance that lies behind the lens and in front of the retina. The AH circulates in the eye by being continuously produced and released. AH is produced in the ciliary body of the eye, and 2.5 μL is produced per minute.^89^ The secreted AH passes through the anterior chamber and is discharged through the trabecular meshwork (Figure 4a). Also, part of the AH flows into the vitreous chamber and maintains the composition of the VH. The balance between these two processes regulates the volume and pressure of the eye fluid, and if there is an imbalance in secretion and reabsorption, function changes and disease conditions can occur.

In general, AH is produced through three distinct processes, i.e., diffusion, ultrafiltration, and active secretion.^89,90^ Active secretion is the major contributor to the production of AH, which takes up about 80–90% of the total amount of AH.^91^ Active secretion mainly occurs in nonpigmented epithelial cells of the ciliary body through the trans-cellular movement of ions, sodium, chloride, and bicarbonate. It occurs selectively to various substances through the blood-aqueous barrier (BAB), and it is mediated by protein transporters in the cell membrane. Water follows the movement of ions through osmosis, resulting in the net secretion of AH in the posterior chamber of the eye. Ultrafiltration is a process by which a fluid is filtered through a semipermeable membrane. Ultrafiltration mainly transfers water and soluble substances. In addition, whether to pass is determined according to the size and charge of the substances that are passing through. In the diffusion of AH, mainly lipid-soluble substances are transported through tissues located between the vitreous chamber and the capillaries due to a concentration gradient of substances across the membrane of the tissue.

The secreted AH passes through the anterior chamber and is secreted into the trabecular meshwork.^92^ The trabecular meshwork is located at the angle between the iris and the cornea, where it forms a series of connective tissue beams and spaces that act as a filter to allow AH to drain out of the eye while retaining the ocular tissues. The trabecular meshwork is divided into three regions, i.e., the uveal meshwork, the corneoscleral meshwork, and the juxtacanalicular tissue (Figure 4b).^93^ The uveal meshwork is located closest to the iris and is composed of large, heavily pigmented cells called melanocytes. The corneoscleral meshwork is located closer to the cornea and is composed of cells called endothelial cells, which form a single layer on the surface of the trabecular beams. The juxtacanalicular tissue is located adjacent to the inner wall of Schlemm’s canal, which is the main drainage pathway for the outflow of the AH. The spaces between the beams are lined with specialized cells called trabecular meshwork cells, and they regulate the flow of AH through the meshwork by controlling the size of the spaces and the movement of fluid. The trabecular meshwork is responsible for regulating approximately 80–90% of the total outflow. Dysfunction of the trabecular meshwork can lead to an imbalance in the production and drainage of the AH.

#### 2.4.3 Ocular Barriers

The blood-ocular barrier system is formed by two main barriers: BAB and the blood-retinal barrier (BRB). This prevents the inflow of toxic substances and protects the eyes by maintaining homeostasis. The BAB is created by tight junctions between nonfenestrated endothelial cells of the iris blood vessels. It is the path through which AH secreted from the blood passes, and is the most important factor in determining the composition of AH. BAB changes in a variety of eye conditions, such as eye inflammation, guided surgery, trauma, or vascular disease, and serves to maintain the difference in chemical composition between plasma and aqueous humor. BAB may not function in the eyes if there is inflammation or disease. It does not prevent white blood cells from penetrating into AH to remove infectious substances, and it can cause various problems such as changing the composition of AH.

In the BAB of the iris, it is difficult for large molecules to pass through tight junctions between cells. However, in the cilia’s BAB, plasma proteins and solutes are allowed to pass through the barrier. Therefore, the substances released through this barrier are part of AH and play an important role in determining its composition.^94^

The BRB is the posterior barrier comprised of retinal pigment epithelium and endothelium cells of retinal blood vessels (inner barrier) with nonleaky tight junctions.^95^ The BRB is an optional barrier to prevent the toxic substances flowing with the blood from destroying nerve tissue. It is particularly tight and restrictive and is a physiologic barrier that regulates ion, protein, and water flux into and out of the retina. The BRB is divided into two parts, the inner BRB and the outer BRB. The inner BRB is formed as tight junctions between retinal capillary endothelial cells. The inner BRB creates a physical barrier that prevents the free diffusion of substances between the capillaries in the retina and the retina. Also, the outer BRB is formed as tight junctions between retinal pigment epithelial cells.

Changes in BRB can lead to retinal diseases. In eye diseases such as diabetic retinopathy and premature infant retinopathy, toxic substances present in blood vessels enter the retina, leading to diabetic retinopathy, and changes in internal barriers lead to AMD.^96^ The BRB is essential to maintain the eye as a privileged site and is essential for normal visual function. Treatment of retinal diseases should be handled by bypassing the BRB using a specific delivery mechanism of the BRB or by intracranial injection either by using its specific transport mechanisms or by circumventing it through intravitreal injections.

The corneal barrier is the physical and biochemical separation of the cornea, which is the outer layer of the eye that covers the iris, pupil, and anterior chamber. The cornea consists of five layers, i.e., epithelial cells, Bowman’s layer, stroma, Descemet’s membrane, and endothelial cells. It is the epithelial cell layer of the cornea that determines the permeation of materials between the corneal barriers. There are tight junctions between the cells of the epithelial cell layer, which determines the permeability of substances between the outside and the anterior chamber.

Tight junction proteins play an essential role in transporting substances between vascular endothelial cells and epithelial cells or maintaining the polarity of cells. Occludin is the first tight junction protein discovered, and it is known that its expression decreases during experimentally induced diabetes or damage to the cerebral blood duct induced by neutral leukocytes. In addition, there are zonula occludens-1 (ZO-1), ZO-2, and ZO-3 members of claudins that have membrane-associated guanylate kinases (MAGUK), which appear to play an important role in cell signaling.^97^ Cells can not only form barriers by forming tight joints, but also transport substances between cells and maintain the polarity of cells.

Various conditions are involved in the efficient passage of drugs between tissues or cells. Drugs administered to the eye have various physical, chemical, and electrical properties, and these properties greatly affect the effectiveness of the drug. In general, drug permeability is determined by the size, charge, hydrophilicity, and solubility of the drug molecule in body fluids. The smaller the size of the molecule, the better the permeability, and the rest of the properties have different effects depending on the characteristics of the tissue. Also, depending on the drug, a special receptor or channel may exist, which can greatly affect the efficacy and permeation of the drug. In the selection of drugs, these conditions must be considered.

The ocular barriers mentioned above are selective barriers that control the substances that enter and exit from the eyeball through tight junctions between cells. Therefore, through understanding the ocular barrier and its components, we will be able to select biomarkers that can be used effectively for the diagnosis of disease.

#### 2.4.4 Clinical Significance

Since the AH and the VH exist inside the eyeball, they are less accessible for the management of disease than the tear film. Nevertheless, the AH and VH are significant clinically due to the relation of various diseases and different compositions. The most important aspect of the AH and VH in disease management is their IOP. The IOP that is maintained by the AH and VH inside the eyeball is a significant indicator of eye health. A high IOP can exert mechanical forces on intraocular tissues, including the retina. The force applied to the tissue due to the high IOP especially damages the retinal part among the intraocular tissues. Therefore, the IOP serves as a major biomarker for ocular diseases, such as glaucoma. In addition, to address these ocular diseases, the flow of the AH must be controlled by drugs. Due to these characteristics, the AH and VH are vital ocular fluids in disease management. Also, most of the AH and VH secreted into the eye consist of water containing small amounts of various substances (Table 3).^98,99^ AH and VH contain various ions, as well as various metabolites such as glucose, cholesterol, amino acids, proteins, oxygen, and carbon dioxide. AH, VH, and tears have similar components, but with slight differences. Therefore, AH and VH are less accessible than externally exposed tears, but can serve as target fluids for disease management.

**Table 3:** Analytes of Aqueous Humor and Vitreous Humor

Analyte	Aqueous humor	Vitreous humor	Reference
Glucose	499 mg L^–1^	67.3 mmol L^–1^	(98, 99)
Total protein	32 mg d L^–1^	4.0 g L^–1^	(98, 99)
Albumin	60 mg L^–1^	1.2 g L^–1^	(98, 99)
Globulin	30 mg L^–1^	2.8 g L^–1^	(98, 99)
Sodium	142 mmol L^–1^	135 mmol L^–1^	(98, 99)
Potassium	4.0 mmol L^–1^	6.6 mmol L^–1^	(98, 99)
Chloride	134 mmol L^–1^	120 mmol L^–1^	(98, 99)
Bicarbonate	20 mmol L^–1^	13 mmol L^–1^	(98, 99)
Urea	4.1 mmol L^–1^	8.6 mmol L^–1^	(98, 99)

## 3 Manageable Diseases through Eyes

Vision plays a more important role in all aspects of our lives than any other sense. However, at least 2.2 billion people worldwide have visual impairment or blindness, one billion of which could have been prevented, but they live with poor eyesight because eye care services are not available. There are many types of visual impairment in the eyes. Cataracts and uncorrected refractive errors are presumed to be the main causes of vision impairment, but other causes of vision impairment can be critically important. AMD, glaucoma, diabetic retinopathy, Amblyopia, Strabismus, infectious eye diseases, and eye trauma related to aging are all important causes of vision disorders that should be solved. Wearable ocular devices can be tools that can effectively manage these diseases of the eye.

The eye has the potential to enable the management of visual impairment as well as metabolic and neurodegenerative diseases. Various substances that function as biomarkers of diseases included in ocular fluids.^31,100,101^ Metabolic diseases, such as diabetes or hyperlipidemia, can alter the concentration of these substances that make up the ocular fluid. Ocular fluids also contain potential biomarkers of neurodegenerative disease. Although the exact correlation between various substances, including neurotransmitters and proteins in the ocular fluids, and neurodegenerative disease is still largely unknown, monitoring of neurodegenerative disease potentially can be demonstrated.

Here, we introduce the representative diseases that can be managed with SCL and retinal prosthesis in detail. Vision-related diseases include glaucoma, RP, AMD, and various other metabolic and neurodegenerative diseases that will be described.

### 3.1 Glaucoma

Glaucoma is an optic neuropathy in which the optic nerve, which carries visual information from the eye to the brain, is damaged, resulting in progressively narrower vision and eventually irreversible blindness.^102−104^ The cause of glaucoma is associated with chronic RGC degeneration, damage of retinal axons, and optic disc excavation (rearrangement of lamina cribrosa), but the pathological mechanism for it is not clear. IOP is a major risk factor for glaucomatous eyes, therefore, reduction of IOP is the only effective strategy clinically for the progression of disease in glaucoma.

In the 1850s, von Graefe described for the first time a condition that low-tension or glaucoma without high pressure can lead to blindness.^105^ In addition to IOP, there are some experimental patients with glaucoma who have demonstrated that advanced age,^106^ race,^107^ high myopia,^108^ and glaucoma-positive family history may be involved in RGC degeneration. Increased IOP may cause ocular ischemia, because the vascular perfusion pressure is decreased by elevated IOP. Glaucoma is the second leading cause of blindness in the world. More than 76 million people worldwide have glaucoma, and the number is expected to reach 112 million by 2040.^109^ Many forms of glaucoma have no warning signs, and once the optic nerve is damaged, it is difficult to repair. Therefore, it is important to have regular eye exams that include measurements of eye pressure.

#### 3.1.1 Types of Glaucoma

As mentioned above, eyes produce a fluid called AH that maintains the IOP and nourishes them. The AH is secreted from the ciliary epithelium and passes through the trabecular meshwork, which is a drain after coming out in front of the iris through the pupil. The AH leaves the eye through the trabecular meshwork into Schlemm’s canals located between the iris and cornea.^110^ The fluid drains out through an area called the drainage angle, which is located where the cornea and the iris meet. As much as the amount of AH that has flowed out, the ciliary body is regenerated, and the rate of production must be balanced by an equal rate of outflow of AH. When the eye makes too much AH or the drainage system does not work properly, extra fluid increases the pressure in the eye, and this pressure is transmitted by way of the vitreous to the optic nerve head and retina. Abnormally IOP blocks axoplasmic flow in the optic nerve, resulting in a lack of neurotrophic factors such as brain-driven neurotrophic factor (BDNF) or nerve growth factor (NGF) and oxygen supply becomes unwanted ischemia. These abnormal manifestations cause RGC dendrites to form dendritic arborization, resulting in potential visual field defects.^111^ IOP is the pressure exerted by AH and is determined by the balance between the production of AH and excretion from the eye. A number of studies have reported that a chronic elevation in IOP induces axonal degeneration and the apoptosis of RGCs.^112,113^ In addition, RGSs exhibit a dendritic response as well as synaptic changes following chronic IOP elevation.^114^ As such, IOP is the main cause of glaucoma and serves as an important biomarker for glaucoma diagnosis. Therefore, for the management of glaucoma, it is important to accurately measure IOP and manage its value.

Glaucoma can be categorized into three groups, i.e., primary angle-closure glaucoma (PACG), primary open-angle glaucoma (POAG), and normal tension glaucoma (NTG). PACG is the rapidly progressive glaucoma, and it occurs when the iris is very close to the drainage angle in the eye.^115^ The iris can end up blocking the drainage angle. When the drainage angle gets completely blocked, eye pressure rises very quickly, and this is called an acute attack. Acute PACG is a medical emergency and requires immediate attention. POAG is the most common type of glaucoma, and the drainage angle of the eye remains open. Despite the drainage angle being open, AH does not drain through the TM. It is a particularly dangerous eye disease because there are no pains, no symptoms, and no vision changes occur at first. POAG develops slowly, so many people are not aware of the problem until significant vision loss has already occurred. NTG is subtype of primary, open-angle glaucoma with IOP measurements always being 21 mmHg or less. Despite the IOP in the normal range, NTG is a type of progressive death of RGCs and glaucomatous visual field loss. No one knows the exact reason why the optic nerve becomes damaged when the eye pressure is normal.

#### 3.1.2 Pathophysiological Mechanism of Glaucoma

Degeneration of RGCs, loss of their axons, and damage and remodeling of the lamina cribrosa are the main events of glaucoma pathogenesis.^102−104^ The glial cells of the retina have a self-defense system that consists of microglia and astrocyte that support and protect neurons by the necessary substances to them and maintaining homeostasis for a suitable chemical environment.^116,117^ Glia cells, which play a major role in the structural and functional stabilization of retinal neurons, are involved in RGC degeneration in experimental glaucoma animal models, and the condition is generated by elevated IOP.^116,117^ Three main types of glial cells are found in the mammalian retina, i.e., astrocytes, Müller cells, and resident microglia. Müller cells are the major type of glial cells in the retina, and the number of Müller cells in the retina accounts for 90% of total glial cells.^118^ Müller cells are characterized by a high hyperpolarized resting membrane potential (about −80 mV) and express different subtypes of inwardly rectifying K^+^ (K_ir_) channels in different membrane domains, which is the basis for the formation of resting potentials.^118^

Müller serves an important role in supporting neurons and the modulation of retina metabolism, water homeostasis, and the regulation of retinal vascular permeability.^118,119^ Müller cells are distributed in a funnel shape in the retinal ganglion cell layer (endfoot), which is essential for light transmission in the retina. When retinal cells are damaged, these cells develop gliosis.^120^ One of the major functions of Müller cells is the recycling of neurotransmitters by their transporters^118^ and the production of neurotrophic factors, such as the BDNF,^121^ the ciliary neurotrophic factor (CNTF),^122^ and the pigment epithelium-derived factor (PEDF).^123,124^ Previous studies on neuronal generation in the retina of zebrafish have been analyzed in Müller cells,^125^ but this kind of regenerative capacity is completely lost in mammalian retina.^126^

Multiple changes occur in Müller cells under pathological conditions, such as the release of various stress signals, disruption of the retinal architecture, changes in the excitatory synaptic transmission, and changes in the synthesis and release of both neuroprotective and detrimental factors.^127,128^ Risk factors of RGC damage include glutamate excitotoxicity,^129^ oxidative stress, neuroinflammation, altered mitochondrial dynamics, and other mechanisms that can lead to changes in retinal homeostasis in the microenvironment.

##### 3.1.2.1 Glutamate Toxicity

Glutamate is a major excitatory neurotransmitter in the CNS of vertebrates^130^ and it plays an important role in the transmission of visual information between bipolar cells, photoreceptors, and RGCs.^131^ Numerous studies have reported that glutamate excitotoxicity is involved in glaucoma.^132,133^ When glutamate is released from presynaptic neurons, it specifically binds to *N*-methyl-d-aspartic acid (NMDA) subtype receptors to postsynaptic neurons, and calcium influx the glutamate-gated Ca^2+^ channels. The increase in intracellular calcium acts as a second messenger that serves as various intracellular signaling cascades.^134^ Glutamate secreted by presynaptic neurons is taken up by excitatory amino acid transporter-1 (GLAST, EAAT-1) in Müller cells and converted to glutamine by glutamine synthase (GS).^135,136^ Glutamine is transported back into the retinal neuron, and the glutamine is converted to glutamate by the glutaminase enzyme in the neuron.^135−137^ When these glutamate-glutamate cycling systems are disrupted, the persistence of glutamate at neuronal junctions leads to a constant influx of Ca^2+^ into the cell through channels, which causes the neuron to become overexcited and die (excitotoxicity).

##### 3.1.2.2 Inflammatory Factors

There are two types of immune system in humans, i.e., innate immunity and adaptive immunity. The innate immune system is activated immediately after infection and is responsible for the early stages of infection so that the microorganisms do not proliferate. Microorganisms have unique pathogen-associated molecular patterns (PAMPs), and the cells responsible for innate immunity (natural killer cells, dendritic cells) have pattern recognition receptors (PRRs) that can recognize these PAMPs. In mammals, PRRs consist of Toll-like receptors (TLRs), NOD-like receptors (nucleotide-binding and oligomerization domain, LLRs), C-type lectin receptors (CLRs), and RIG-like receptors (retinoic acid-inducible gene-I-like receptors, RLRs).^138−140^ They are crucial for recognizing foreign pathogens and activating the immune response to defend against them.

TLRs are membrane receptor proteins, and subtypes from TLR1 to TLR13 have been found. In humans, TLR1–10 are functional, TLR11 is present but not functional, and TLR12–13 are not expressed.^141^ Among them, TLR2, TLR4, and TLR5 present in the plasma membrane are known to recognize lipoprotein, lipopolysaccharide (LPS), and flagellin.^142−144^ TLR3, 7, 8, and 9 are located in the endosome and recognize dsDNA and ssRNA of the virus and play an important role in the defense mechanism against viral infection.^145−147^ TLR2 is expressed in microglia, TLR3 in astrocyte, and TLR4 in trabecular meshwork.^148^ Immunohistochemical analysis and proteomic assay in glaucoma patient samples showed that various TLRs, including TLR4, were expressed in microglia and glial cells, and it has been reported that the expression of heat shock protein (HSP) and oxidative stress was increased through TLR signaling.^148^ These results also were confirmed in glaucoma animal models. Rapid and continuous increase in IOP activates TLR4 and promotes the activation of caspase-8, leading to retinal cell apoptosis due to the production of inflammatory proteins.^139^ When TLR ligands bind to TLRs, mechanisms such as intracellular pro-inflammatory genes and nuclear factor-kappa beta (NF-κB) are activated, and the expression of pro-inflammatory factors is induced. However, overexpression of TLRs can cause an autoimmune disease^149^ that attacks the body by increasing the immune response excessively.

Retinal inflammatory response is implicated in the pathogenesis of glaucoma and in glaucomatous conditions, and Müller cells undergo reactivation (gliosis).^118,150^ Under pathological conditions, activated retinal glial cells fail to play their neuroprotective roles and promote widespread inflammatory factors, including tumor necrosis factor-alpha (TNF-α) and nitric oxide (NO) and interleukins.^127^ TNF-α is a major inflammatory factor and release from activated Müller cells and microglia glia. These factors aggravate cytotoxic reaction and RGC degeneration. In glaucomatous retina, TNF-α could activate death signals, such as caspase-8, and oxidative stress.

##### 3.1.2.3 Oxidative Stress

Oxidative stress is a state of excessive production of endogenous and exogenous reactive oxygen species (ROS) in the body.^151^ Activated ROS cause toxicity, affect the function of the mitochondria, affect an organelle in the cell, and shorten the length of the telomere in the nucleus.^152^ It is known that astrocytes produce protein protease, matrix metalloproteinase (MMP) to remodel the extracellular matrix,^153^ thereby inducing optic disc depression and retinal ganglion cell axon deformation. In particular, chronic oxidative stress activates microglia in the retina and increases the production of inflammatory cytokines (IFNγ, IL-6, IL-10, IL-1β) through unregulated inflammatory reactions.^154^

As mentioned above, the main cause is IOP, but various substances are expressed due to various problems induced by IOP. Therefore, based on the pathophysiological mechanism, IOP plays a key role in diagnosing and managing glaucoma. However, there are cases that cannot be judged by IOP, such as NTG. In this situation, it is expected that glaucoma can be accurately diagnosed using substances such as inflammatory cytokines and TNF-α as biomarkers.

### 3.2 Photoreceptor Degeneration

In glaucoma, ganglion cells are damaged by mechanical pressure, which causes irreversible loss of vision. Similarly, RP and AMD also are diseases that can cause the loss of vision, but the mechanisms are different from glaucoma. These are diseases caused by problems with photoreceptors, not ganglion cells. Any dysfunction of ganglion cells blocks the transmission of signals to the brain, while degeneration of photoreceptors blocks the reception of light. Understanding these mechanisms are significant in diagnosing the disease or possibly restoring vision. Therefore, in this section, we will introduce the mechanisms of RP and AMD along with the conventional diagnosis methods.

#### 3.2.1 Retinitis Pigmentosa

RP is an inherited retinal disease that is characterized by progressive degeneration of photoreceptors, leading to the loss of vision.^155^ Most of the degeneration of the photoreceptors is caused by gene mutations that affect the function of the photoreceptors. RP can be divided into 3 stages according to the progress of the disease. In the early stage of RP, the first symptom is the loss of night vision. Patients do not notice any discomfort in vision during the daytime with sufficient light, while the peripheral visual fields start to diminish at night with dim light. In the midstage, peripheral vision loss in the dim light condition is apparent, and patients perceive the diminution of peripheral visual fields even in the daytime along with the decrease in visual acuity. Moreover, dyschromatopsia, which refers to dystrophy in the recognition of colors, has occurred in several patients with severe degeneration in cone cells. If the RP progresses further to reach the end stage, the patients could not autonomously move by themselves due to their loss of peripheral, i.e., they have tunnel vision. In addition, patients can undergo loss in the central visual field, which causes enormous hindrances in normal daily life.

RP is initiated by the degeneration of photoreceptors, especially rod cells.^156^ The major cause of the degeneration is over 60 gene mutation that is inherited from the parents, and most of the gene mutations related to the RP affect the metabolic system of rod cells.^157^ Otherwise, mutated genes are expressed in RPE or in phagocytosis in the outer segments of photoreceptors, which is crucial for photoreceptors to be functionalized normally. In short, mutated genes are expressed and cause degeneration or cell death in rod cells in the first place, and the subsequent degeneration of cone cells proceeds in the end stage of RP, leading to the overall degeneration of photoreceptors.

The mutation of genes regarding the degeneration of rod cells triggers several pathways of cell death mechanisms, such as apoptosis, regulated necrosis, and autophagy. The representative pathways include oxidative stress, endoplasmic reticulum (ER) stress with Ca^2+^ regulation, and an imbalance between autophagy and the ubiquitin-proteasome system (UPS).^158^ Also, damaged rod cells bring about inflammatory response and innate immune response, resulting in the further death of rod cells.

**Figure 5:**
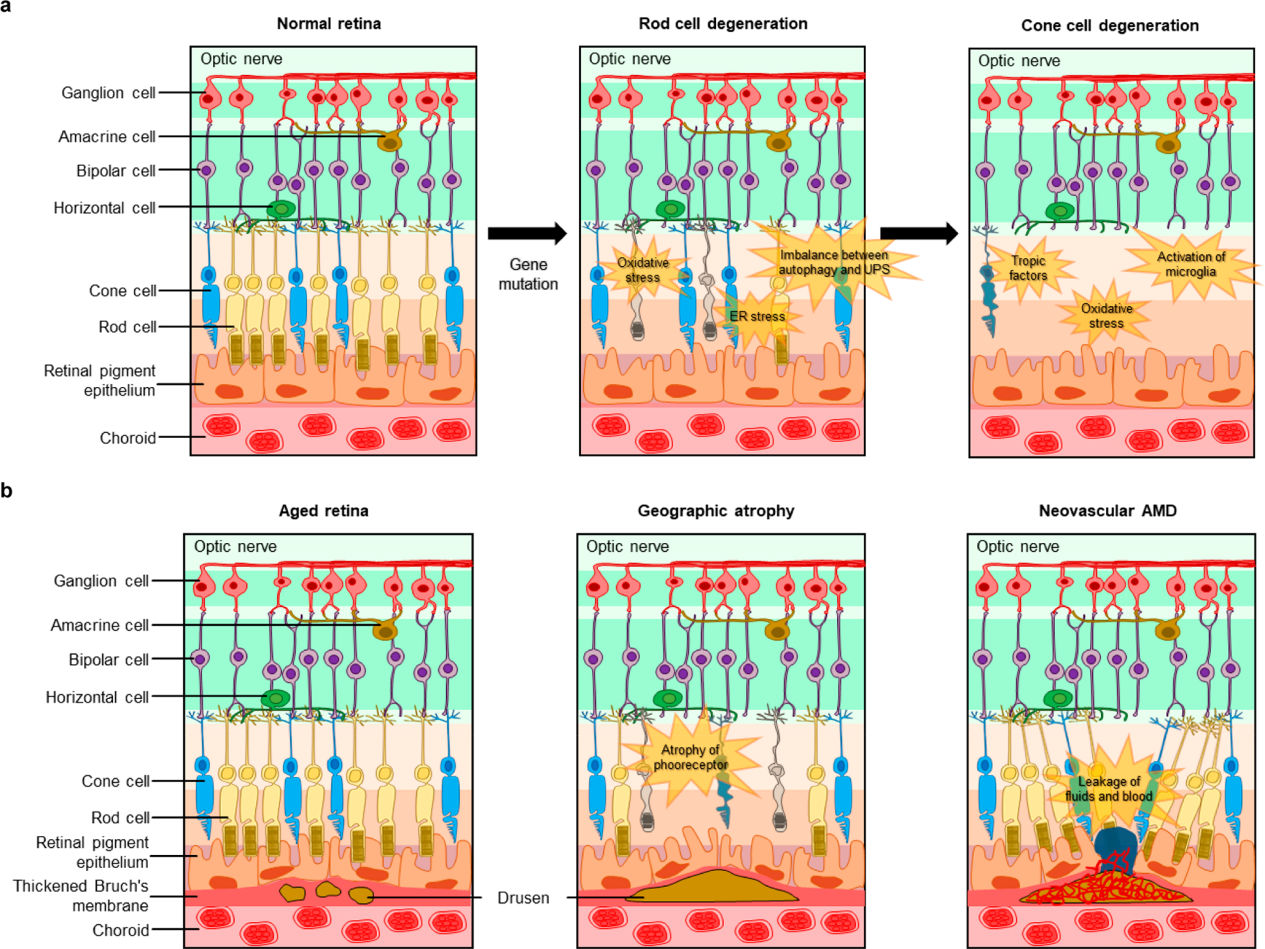
Photoreceptor degeneration. (a) Schematic illustrations describing pathogenesis of retinitis pigmentosa and (b) age-related macular degeneration.

Sequentially, the degeneration of cone cells occurs in the later stages of RP, and several theories explain how the degeneration of rod cells affects the degeneration of cone cells or how cone cells are protected until the late stage of the RP.^159,160^ One of the theories is related to the trophic factors that regulate the survival of cells. Some discoveries have revealed that healthy rod cells release neurotrophic factors that are necessary for the survival of healthy cone cells. Thus, the presence of healthy rod cells prevents the degeneration of cone cells, which can explain the late degeneration of cone cells. Another hypothesis suggests that oxidative stress triggers the degeneration of cone cells since the reduced level of oxygen consumption by the degeneration of rod cell leads to an increment in oxygen level for cone cells. Also, several studies have demonstrated that the degeneration of rod cells induces the activation of microglia and the release of cytokines, such as TNF-α, leading to apoptosis of the cone cells. The overall pathogenesis of RP is described in Figure 5a.

The exact mechanisms that explain the reasons the cone cells deteriorate in the later stage of the RP are still unknown. Clarification of the complex mechanism of secondary degeneration of cone cells will pave the way to discovering specific treatments for retinal diseases.

#### 3.2.2 Age-Related Macular Degeneration

AMD is a progressive impairment of vision, which is caused by chronic degradation of the macula, which is the part of the retina that is related to the central vision and exhibits a high concentration of photoreceptors.^161^ As the term indicates, this disease occurs as people get older and the functions of individual retinal layers, especially RPE, Bruch’s membrane, and choroid, get weaker. AMD defects the central field of vision and eliminates the detection of fine details.

AMD is a condition that is characterized by the formation of drusen due to the aging of the retina.^162,163^ Drusen are formed by an accumulation of basal deposits called lipofuscin in Bruch’s membrane. These deposits are produced by the photoreceptors that convert optical information into electrical signals. RPE is responsible for phagocytosis, which is the process of removing cellular waste and debris, to eliminate the deposits from the outer segments of the photoreceptor. However, as aging progresses, the phagocytic function of RPE gets enfeebled, and lipofuscin remains in Bruch’s membrane. The aging also affects the density and thickness of the capillary vessel, as well as the bloodstream in the choroid, which supplies oxygen and nutrients to the RPE for normal phagocytosis. This degradation in choroidal function results in insufficient removal of the deposits. Moreover, the thickening of Bruch’s membrane obstructs the diffusion of oxygen and nutrients from the choroid to RPE, which further hinders the overall removal of deposits from the photoreceptors, leading to the formation of drusen.

The size and the number of drusen is the indicator to diagnose the stages of AMD.^164^ Advanced AMD is characterized by the presence of drusen and shows geographic atrophy or neovascularization (Figure 5b). In geographic atrophy, there is a progressive atrophy of photoreceptors, RPE, and choriocapillaris, which results in a slow loss of vision. In neovascular AMD, choroidal vessels are created in the space developed by the large drusen, and they are formed from the accumulation of small drusen. These abnormal vessels damage the weakened retinal layers, leading to the leakage of fluids and blood. This is the why neovascular AMD is referred to as wet AMD. Neovascular AMD causes rapid loss of vision, and the patients may undergo sudden loss of central visual fields or scotoma where the partial visual fields appear as blind spots. Moreover, the recruitment of activated macrophage and microglia at the neovascularization lesion causes cellular damage by secreting chemokines and cytokines. This further exacerbates the degradation of retinal layers, leading to vision loss that is even more severe.

#### 3.2.3 Diagnosis of Retinitis Pigmentosa and Age-Related Macular Degeneration

**Figure 6:**
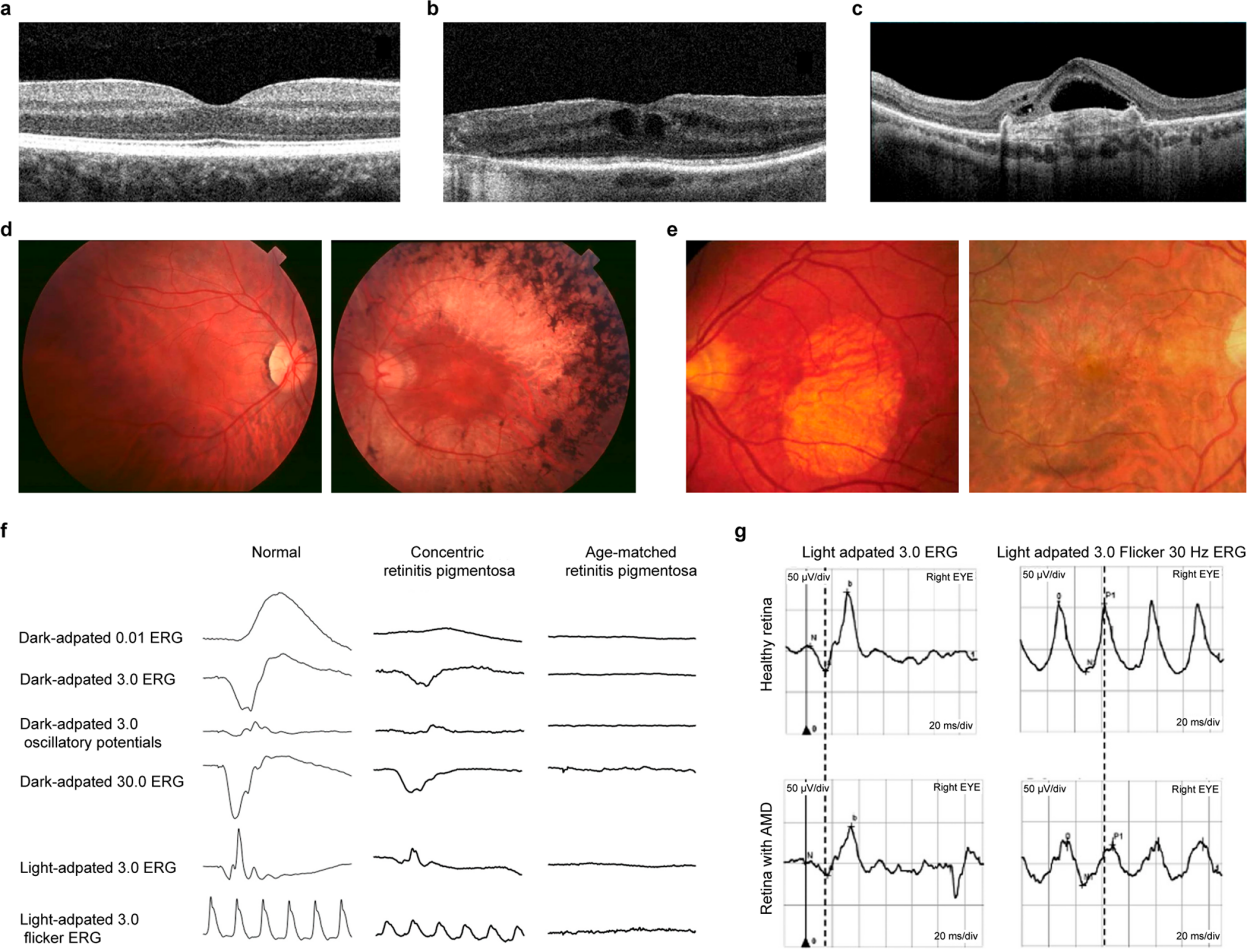
Diagnostic methods of retinal diseases. (a) OCT image of normal retina. (b) OCT image of RP patient. (a, b) Reproduced with permission from ref (166). Copyright 2022 MDPI under CC BY 4.0 (https://creativecommons.org/licenses/by/4.0/). (c) OCT image of AMD patient. Reproduced with permission from ref (169). Copyright 2021 MDPI under CC BY 4.0 (https://creativecommons.org/licenses/by/4.0/). (d) FAF images of RP patient at early stage (left) and midstage (right). Reproduced with permission from ref (171). Copyright 2006 Springer Nature under CC BY 2.0 (https://creativecommons.org/licenses/by/2.0/). (e) FAF images of AMD patient with atrophy (left) and neovascularization (right). Reproduced with permission from ref (174). Copyright 2009 Elsevier. (f) Representative changes in ERG for RP patient. Reproduced with permission from ref (177). Copyright 2021 MDPI under CC BY 4.0 (https://creativecommons.org/licenses/by/4.0/). (g) Representative changes in ERG for AMD patient. Reproduced with permission from ref (178). Copyright 2020 John Wiley and Sons.

Diagnosis of retina-related diseases, i.e., RP and AMD, can be accomplished by regular eye examinations that include dilated eye examination with a visual field test. These basic tests are essential to detect the disease in the early stage because this could lead to the application of agile and effective treatments. Along with this regular examination, imaging techniques can be applied to detect the symptoms and progress of the retinal degenerative disease. Optical coherence tomography (OCT) is the most common technique that noninvasively captures the retinal layers, including epiretinal membranes, macula, and swelling of the macular (Figure 6a).^165,166^ This observation method can measure the thickness of the retina and examine the integrity of the retinal layers, which can change due to the degeneration of the photoreceptors. OCT scan images of RP patients show abnormal lines between the photoreceptors’ inner and outer segments (Figure 6b).^166,167^ For AMD patients, OCT scan images present the existence of the drusen, which can be a clue for the development of AMD (Figure 6c).^168,169^ Another imaging technology is fundus autofluorescence (FAF), that images the blood vessels of the eye by detecting the ocular fluorophore, lipofuscin, without injection of the fluorescein dye.^170−174^ The abnormality of the retina due to the diseases appear as a hyperautofluorescence region. The representative FAF images of RP and AMD patients are presented in Figures 6d and 6e.

As an ophthalmic electrophysiology test, an ERG test is conducted to examine the electrophysiological responses of the retina to light. Ophthalmologists observe amplitudes and the implicit time of a- and b-waves of ERG. The ERG of early stage RP patients has decreased amplitudes of waves and prolonged implicit time. Moreover, the ERG of RP patients in the advanced stage is not detectable due to the excessive degeneration of the photoreceptors (Figure 6f).^175−177^ In the case of AMD, light-adapted 3.0 a-wave implicit time and 30-Hz flicker peak time are prolonged compared to healthy control (Figure 6g).^178^ Since ERG is a noninvasive method that shows distinguishable features depending on the disease, ERG tests are implemented universally to diagnose and examine retinal degenerative diseases.

We have introduced retinal degenerative diseases, which eventually leads to vision loss and cause discomfort in daily life without an assistant. Unfortunately, there is no ultimate treatment to restore the impaired vision due to the degeneration of retinal cells. Moreover, the treatment for delaying the progress of disease is intravitreal injection of chemicals; while this procedure is minimally invasive, it can evoke repulsion from patients who are undergoing vision loss. With the advances in technology of bioelectronics, retinal prosthesis has arisen as a therapeutic assistant system that electrically stimulates the vision process to produce a synthetic vision for RP or AMD patients. This will be discussed later in section 7.

### 3.3 Metabolic Diseases

#### 3.3.1 Various Types of Metabolic Diseases

Metabolic diseases are conditions that disrupt normal metabolism in the body, including the breakdown of carbohydrates, proteins, vitamins, and lipids. These abnormal metabolisms can occur for several reasons, including generic factors (heredity, disease of the internal secretory organs, etc.) and environmental factors (poor eating habits or overeating, lack of exercise, etc.). These disorders can result in serious health consequences, such as diabetes, hyperlipidemia, gout, and hyperkalemia. The prevalence of metabolic diseases has been increasing globally, making it a significant public health concern. Effective prevention and management strategies are needed to address these problems and improve the overall health and well-being of individuals affected by metabolic disease. Biomarkers are factors that can be used to diagnose and monitor the progression of the disease. Metabolic diseases arise due to dysregulation in metabolic pathways, resulting in the abnormal presence of metabolic molecules that can serve as biomarkers. By managing these biomarkers, personalized healthcare for patients with metabolic diseases can be improved.

Diabetes is one of the carbohydrate metabolism disorders. Diabetes affects the body’s ability to use or store glucose. Therefore, glucose cannot be used as an energy source, because it would result in high levels of glucose in the blood, which can cause serious damage to other organs, such as the eyes, the heart, and nerves.^179^ An important thing to note is that there is a possibility of expansion for complications (retinopathy, nephropathy, cardiovascular comorbidities).^180^ Type 2 diabetes (T2D) is a disease in which the blood glucose level rises due to a disorder in insulin secretion and is the most common presentation of diabetes, representing 90% of patients.^181^ Type 1 diabetes (T1D) is a chronic disease in which insulin is not secreted or very little is secreted because of the destruction of β-cells in the pancreas. T1D typically develops before the age of 30 and is commonly referred to as juvenile diabetes. Diabetic macular edema (DME) and diabetic retinopathy are common complications of diabetes, which is a leading cause of vision loss.^182^ More than 500 million people suffer from diabetes, and the number is predicted to rise to 783 million by 2045.^183^ The concentration of glucose can be measured as a biomarker for diagnosing diabetes using two methods, i.e., (1) fasting plasma glucose (FPG), which measures glucose concentration after an 8-h fast, and (2) oral glucose tolerance test (OGTT), which measures blood glucose levels 2 h after drinking a sugary solution.^184^ A diagnosis of diabetes is made when FPG levels are 126 mg dL^–1^ or higher, and when OGTT levels are 200 mg dL^–1^ or higher. Furthermore, hemoglobin A1c (HbA1c), formation to the amino-terminal group when glucose attaches, is another biomarker to diagnose diabetes. An HbA1c level of 6.5% or higher is considered to indicate diabetes.^185^ Many substances such as insulin, C-peptide, fructosamine, and glycated albumin can be utilized as biomarkers for diabetes.

Hyperlipidemia is a condition of lipid metabolism, which is characterized by abnormally high levels of lipids in the blood. This condition can interfere with the body’s ability to properly break down and utilize lipids. This elevated lipid level can cause atherosclerosis where plaque builds up on the wall of arteries, making the arteries narrow. This process can lead to decreased blood flow to various organs and can ultimately lead to CVD such as cardiac failure, myocardial infarction, and stroke.^186^ The biomarkers used to diagnose hyperlipidemia include total cholesterol (TC), low-density lipoprotein cholesterol (LDL-C), and triglycerides (TG). Hyperlipidemia is defined by having a fasting TC concentration of more than 200 mg dL^–1^, an LDL-C level of more than 130 mg dL^–1^, and TG levels of more than 150 mg dL^–1^.^187^ In addition, apolipoprotein B (ApoB), a protein found in LDL-C, also can be measured as a biomarker. Elevated levels of ApoB may indicate an increased risk of CVD.^188^

Gout is another metabolic disease that is based on purine metabolism. Gout passes 4 stages, i.e., asymptomatic hyperuricemia, acute gout, intercritical gout, and chronic tophaceous gout.^189^ Purine metabolism is the process by which the body produces and breaks down purines, which are nitrogen-containing compounds that are used in the synthesis of DNA and RNA, as well as in the production of energy and other cellular processes. The final product of this metabolism is uric acid. A high uric acid level is defined as hyperuricemia, which occurs due to the overproduction of uric acid, a high purine diet, or impaired renal excretion of uric acid. In the acute gout stage, an unexpected attack might occur. Monosodium urate (MSU), a salt derived from uric acid, refers to crystals that form in the joints and cause severe inflammation and acute gout attacks,^190^ which are accompanied by severe pain, swelling, and redness in the joints. The third stage, intercritical gout, is defined as the period between acute attacks.^191^ Failure to manage uric acid levels at this stage can result in a second attacks, which invades multiple joints and can be painful for longer. Chronic tophaceous gout is the final stage which is the severe stage of gout. Tophi, a visible collection of MSU, can form in the joints and tissues and can cause chronic pain and damage, causing complications.^192^ This stage typically is the result of years of uncontrolled hyperuricemia. Proper management and treatment can prevent the progression of gout. As such, the primary biomarker of hyperuricemia and gout is uric acid, which is considered as hyperuricemia when the concentration is above 7.0 mg dL^–1^. Furthermore, several studies have shown a positive association between uric acid levels and inflammatory proteins, such as including C-reactive protein (CRP) and IL-6.^193^ This association suggests that inflammation may be a contributing factor in the progression of gout.

Hyperkalemia is a metabolic problem that refers to an abnormally high level of potassium in the blood, which is caused by decreased kidney function and impaired circulation of potassium into the cells.^194,188^ Potassium is an essential electrolyte that is involved in the electrical activation of the body, including the function of muscles, acid–base balance control, and others. However, if the potassium level is too high (more than 5.5 mequiv L^–1^),^195^ it can result in headaches, irregular heartbeat, and further serious electrocardiographic abnormalities.^196^ In addition to potassium, blood pH is another biomarker for hyperkalemia, as disruptions in acid–base balance control can lead to changes in pH levels.

Furthermore, there are numerous metabolic disorders that can be classified into metabolic components. First, carbohydrate metabolic disorders include diabetes, glycogen storage diseases that cannot store or break down glycogen and galactosemia and lactose intolerance that cannot metabolize galactose and lactose in dairy products.^197^ Moreover, amino acid metabolism disorders are diseases that affect the body’s ability to utilize proteins properly, such as phenylketonuria, maple syrup urine disease, and homocystinuria.^198^

Metabolic diseases often are interconnected and can lead to the development of other conditions. As many of these diseases are chronic, early diagnosis and consistent treatment with biomarkers are crucial for managing and preventing complications.

#### 3.3.2 Biomarkers for Metabolic Diseases in Ocular Fluids

Tears contain complex mixtures of proteins, lipids, electrolytes, and small molecule metabolites that are possible candidates for biomarkers of metabolic disease. First, proteomic studies have identified over 2,000 different proteins in tears that maintain ocular surface homeostasis,^199^ with abundant lysozyme, lipocalin, albumin, immunoglobulins A (IgA), lactoferrin, and lipophilin. In addition, lipids (cholesterol, cholesteryl ester, phospholipids), small molecules (uric acid, ascorbic acid, lactate, glucose), electrolytes (sodium, potassium, calcium, magnesium) are contained in tears.^81−83^ Among these substances, several constituents have been found to correlate with blood levels and can serve as biomarkers for metabolic diseases. For instance, the correlation between blood and tear glucose levels has been studied, with tear glucose levels reflecting blood glucose levels with a time delay of around 10 min.^200−202^ Furthermore, Song et al. conducted tests on rabbits that had been fed a cholesterol-rich chow diet, and the results of the tests revealed that cholesterol levels had increased for several weeks in both tears and blood, indicating that cholesterol in tears can be used as a biomarker to reflect blood cholesterol levels.^203^ These findings suggest that tears have the potential to serve as a noninvasive tool for personalized medicine and healthcare in the context of metabolic diseases.

AH is a transparent fluid located between the lens and the cornea,^199^ and it is composed of water, proteins (cytokines), electrolytes (sodium, potassium, chloride, calcium), and metabolites (glucose, uric acid, urea, lactate). Similar to tears, AH also contains various biomarkers that can provide information about the status of one’s health. Some substances in AH have similar concentrations in the blood due to plasma leakage. For instance, the level of sodium ions in plasma and AH is similar and glucose in the AH is about 80% of plasma levels.^89^ And the concentration of uric acid is increased in the AH of retinoblastoma patients. Furthermore, intercellular adhesion molecule-1, a cytokine in AH, has been identified as a biomarker for DME.^182,204^ Additionally, this tendency suggests the possibility that molecules in the AH can be used as a biomarker for metabolic disease. However, since AH is located inside the eye, the current method of obtaining it is invasive and can be dangerous for patients. Therefore, noninvasive methods for measuring biomarkers in AH are needed to improve the accuracy of diagnosing metabolic diseases.

VH is a gelatinous mass that fills the space between the lens and the retina. It is composed of proteins (more than 1000 kinds, including prealbumin, transferrin, and collagen),^205^ electrolytes (sodium, potassium, chloride), and metabolites (glucose, creatine, urea).^206^ Because of the blood-vitreous barrier, numerous biomolecules that can serve as biomarkers for metabolic diseases can be found in the VH.^206^ While there are no blood vessels present in the VH, biomolecules are nourished by vessels of the retina and ciliary epithelium through diffusion, transport, osmotic pressure and hydrostatic pressure.^207^ Due to this barrier, some biomolecules can correlate with molecules in the blood and can serve as biomarkers.^205,207^ For instance, cytokine (vascular endothelial growth factor and IL-6) levels are increased in proliferative diabetic retinopathy eyes.^208^ Moreover, glucose in VH is converted to lactate during the post-mortem period. The total value of glucose and lactate can be used as a biomarker for analyzing diabetes death.^209^ As such, VH is used mainly to clarify the post-mortem diagnosis of metabolic diseases.^210^

There is a correlation between tears, AH, and VH with blood, suggesting that ocular fluids could be valuable in diagnosing metabolic diseases. However, further research is needed to develop standardized methods for analysis and interpretation.

### 3.4 Neurodegenerative Disease and Mental Illness

Neurodegenerative diseases are a group of disorders that involve the progressive loss of function and death of neurons in the brain and nervous system. These diseases typically are chronic and can lead to a range of symptoms, including memory loss, difficulty with movement, and changes in mood and behavior. Various substances contained in ocular fluids can be potential candidates for biomarkers for the management of neurodegenerative diseases.

#### 3.4.1 Biomarkers of Neurodegenerative Diseases

Conventionally, diagnosis and progression monitoring of neurodegenerative diseases, such as Parkinson’s disease (PD), Alzheimer’s disease (AD), and multiple sclerosis (MS), are implemented by imaging or electrodiagnostic tests, including magnetic resonance imaging (MRI), computed tomography (CT), and electroencephalography (EEG) assay. However, these diagnostic methods have limitations in terms of speed, accessibility, and temporal restriction, so they possess the possibility of incorrect diagnosis due to the mismatch of time point and limits on the monitoring of the dynamic progression of the diseases. Therefore, it is crucial to discover biomarkers for continuous monitoring of neurodegenerative diseases to assess the progression of the disease, to predict the symptoms before occurrence, and to examine the responses to the therapies.^211,212^ In addition, it would be possible to predict the risk of developing a disease, which would enable us to deal with the diseases more effectively.

Neurodegenerative diseases bring about a dysfunction of the lacrimal gland since it is innervated by parasympathetic and sympathetic neurons.^213^ The degeneration of these neurons can alter the secretion or the composition of tears. These alterations contribute to discrimination of tear composition from the patients and the healthy controls, leading to identification of biomarkers of neurodegenerative diseases. To date, several studies have discovered biomarkers from tears for neurodegenerative diseases, and the following section introduces the specific neurodegenerative diseases and their corresponding biomarkers.

##### 3.4.1.1 Parkinson’s Disease

PD is one of the most common neurodegenerative diseases that affects the motor function of the body, including tremor, bradykinesia, stiffening muscles, and loss of automatic movements. The main cause of PD is the reduction in the release of dopamine from the substantia nigra, which leads to the accumulation of Lewy bodies, an abnormally deposited protein in neural tissues. The major component of the Lewy body is alpha-Synuclein (α-Syn), which is a well-known biomarker for PD and is also present in tears. α-Syn can be categorized into two categories, i.e., oligomeric and total α-Syn, existing in two different types of tears, i.e., basal and reflex tears. The studies revealed that the level of oligomeric α-Syn in the tears of PD patient increases, while the level of total α-Syn in tears of PD patients decreases compared to the healthy controls for both types of tears.^214,215^ These discriminations of the α-Syn level between PD patients and healthy controls originate from dysfunction of the lacrimal gland due to the degeneration of neurons caused by PD. The difference in level of α-Syn between patients and healthy controls appears larger in reflex tears than in basal tears. The authors presumed that this is due to the larger contribution of the lacrimal gland to reflex tears than basal tears, resulting in a larger difference in the composition of reflex tears from the healthy controls. The other study investigated the content of the oligomeric α-Syn in tears depending on the stage of the PD.^213^ In comparison with healthy control tears, the oligomeric α-Syn increased by 8.6-fold (4.43 ± 1.26 ng mg^–1^ tear protein), 4.5-fold (2.3 ± 0.54 ng mg^–1^ tear protein), 3.7-fold (1.9 ± 0.59 ng mg^–1^ tear protein) in tears of the early, intermediate, and late stage of PD, respectively. In addition to α-Syn, CCL2 and DJ-1 are potential biomarkers in tears for PD. CCL2, a chemokine which activates the inflammatory cells, acts as an effector of progression of PD and is elevated in the content of tears from PD patients. DJ-1, which is a common mutated protein of PD, functions as a redox-sensitive chaperone that prevents the aggregation of α-Syn. The level of DJ-1 in tears also is elevated in the PD patients.

Moreover, physical biomarkers for PD that appeared in the ocular system include decrease in eye blinking and the secretion of tears, which is characterized into dry eye sensation.^216^ Also, as another physical biomarker, visual dysfunction also appears, caused by accumulation of Lewy bodies in the retina and depletion of dopamine as a pathological process of PD.

##### 3.4.1.2 Alzheimer’s Disease

AD is the most common type of dementia; it is a neurodegenerative disease and causes impaired memory, language, and thinking. The main cause of AD is the accumulation of abnormal misfolded proteins, β-amyloid (Aβ), and tau in the brain, leading to the reduction of the release of neurotransmitters, especially acetylcholine. The degeneration of the autonomic nervous system due to AD deteriorates the function of the lacrimal gland, which causes changes in the contents of tear protein. Studies have identified a decline in the levels of lipocalin-1, lacotransferrine, lysozyme-C, extracellular glycoprotein lacritin, and prolactin-inducible protein in the tears of AD patients, while an elevation in the level of dermcidin was noted. Further analysis found that a combination of 4 proteins, i.e., lipocaline-1, lysozyme-C, lacritin, and dermcidin, are effective biomarkers for AD that can be discriminated from the healthy controls.^217^

Furthermore, the pathological progression of AD induces structural changes in the retina, degeneration of retinal neurons, and alternation of blood flow within the retina. These changes in the retina impair visual processing, which can be a physical biomarker for AD.^218,219^ Also, lacrimal gland dysfunction due to AD leads to increase in tear flow rate and teat protein concentration by 6 ± 2 μL min^–1^ to 12 ± 2 μL min^–1^ and 4.4 ± 1.4 μg μL^–1^ to 8.8 ± 2.9 μg μL^–1^, respectively.

##### 3.4.1.3 Multiple Sclerosis

MS is a demyelinating disease that occurs when the insulating covers of cells, known as the myelin sheath, become damaged, resulting in the interruption of neural signal transmission. The damage to the nervous system can cause a wide range of symptoms, i.e., from muscle weakness to vision impairments. While the causes of MS have not been defined clearly, it is believed that the malfunction of the immune system attacking cells in the brain is the most powerful mechanism of neuron degeneration for MS.

Although the discovery of biomarkers in tears for MS has not been implemented actively compared to other diseases, Salvisberg et al. analyzed the tears from patients to find the potential biomarkers of MS.^220^ The authors examined a large number of candidate proteins using three types of analysis, i.e., quantitative proteomic study using tandem mass tag, Western blot, and immunoassay. As a result, the authors suggested alpha-1-antichymotrypsin as a biomarker in tears for MS, which increases in tears from the MS patients with the ratio in the range of 1.6 to 2.5.

#### 3.4.2 Potential Biomarkers in Tears

##### 3.4.2.1 Neurotransmitters

Neurotransmitters are biochemicals that transmit signals between neurons. There are two types of neurotransmitters, i.e., excitatory and inhibitory, and balanced release of both neurotransmitters enables communication among the neurons and control of the biological system. A disorder of the nervous system due to various causes, such as degeneration of neurons or mental illness, can affect the release of neurotransmitters, leading to alterations in normal neurological operation.^221^ In addition, environmental and physiological factors that cause mental illness can lead to abnormal function of the nervous system by disrupting the normal secretion of neurotransmitters.^222^ However, neurotransmitters cannot be considered as biomarkers since they do not exhibit distinguishable concentration levels according to specific diseases and are involved in various biological processes. Nevertheless, monitoring the level of certain neurotransmitters related to the neurodegenerative disease or mental illness can provide mediate indicators regarding the progress of the disease or response to the treatment.

In the same manner as monitoring biomarkers for neurodegenerative disease in tears, continuous detection of neurotransmitters in tears can help to examine dynamic changes in the level of neurotransmitters and to predict forthcoming symptoms. Among the various types of neurotransmitters, dopamine, serotonin, and acetylcholine are found in tears.^223^ Dopamine, which is involved in motor activity and in the reward system of brain, increases in the tears of PD and depression patients compared to healthy controls. However, the levels of dopamine in tears from mania and schizophrenia patients decrease. Serotonin, which regulates mood, emotions, and digestion, shows an increment in content of tears from mania, schizophrenia, and anxiety, while it decreases in tears from the patients of PD and depression. In addition, acetylcholine is present in tears and is involved in memory and muscle functions. The level of acetylcholine in tears increases for patients with depression and decreases for patients with PD and AD.

##### 3.4.2.2 Hormones

Similar to neurotransmitters, hormones are one of the biochemicals that regulate the biological systems of animals. However, hormones are produced in the endocrine gland and released into the bloodstream, whereas neurotransmitters are produced within the cells and released into the interspace of the neurons, synapse. When hormones present in bloodstream pass through the blood vessels in the conjunctiva, they can enter tears by plasma leakage. Thus, monitoring of hormones in tears can be implemented to examine the biological conditions of individuals. Hormones, as well as neurotransmitters, are not definitive biomarkers since they involve various ranges of biological processes, from homeostasis to immune systems. However, alternation in the level of specific hormones reflects the certain state of the biological system. For example, cortisol is one of the hormones that regulates metabolism and formulates memories. Numerous studies have demonstrated that the level of cortisol reflects the stress level.^224,225^ As the stress level increases the risk of developing mental illness, monitoring of the cortisol level can immediately indicate the status of mental health, such as anxiety.

Still, there are certain limitations for using tears for the diagnosis of neurodegenerative diseases or mental illness. First, the discovery of biomarkers for those diseases in tears is still in progress. Thus, biomarkers in tears cannot be used as definite tools for providing reliable information about the pathogenesis. Also, the small content of biomarkers in tears requires sensitive sensors to detect the minuscule amount of the biomarkers. Nevertheless, along with the enormous advancements in the SCL, monitoring of biomarkers related to the neurodegenerative diseases or mental illness in real-time is conceivable, which can provide an opportunity to create a new platform for delicate management in the progress of disease.

In addition to the biomarkers mentioned here, there are various biomarkers depending on the disease (Table 4).^186,193,214,217,226−239^ The SCL is expected to be able to accurately diagnose diseases through a complex analysis of these biomarkers.

**Table 4:** Various Types of Diseases That Can Be Diagnosed through Ocular Biomarkers

Type of disease	Diseases	Biomarker	Reference
Eye diseases	Glaucoma	Intraocular pressure	(226)
	Keratoconus	IL-6, TNF-α, MMP-1	(227)
	Allergic conjunctivitis	Ig gamma-2, leukocyte elastase inhibitor, sPLA2-IIa	(228)
	Blepharitis	Serum albumin, α-1-antitrypsin, lacritin, lysozyme, Ig-k chain VIII, prolactin inducible protein, cystatin-SA III, pyruvate kinase, uncharacterized protein	(229)
	Diabetic retinopathy	Nerve growth factor, apolipoprotein A-I, lipocalin 1, heat shock protein 27, beta-2 microglobulin, endothelin, neuron-specific enolase	(228)
	Dry eye	Lysosome, lactoferrin, calgranulin A(S100A8), MMP-9	(229)
	Retinitis pigmentosa (RP)	Outer segment thickness (OST), choroidal thickness, monocyte chemoattractant protein 1 (MCP-1)	(230, 231)
	Age-related macular degeneration (AMD)	Retinal pigment epithelium-drusen complex (RPEDC) volume, endothelin-1 (ET-1), Nitric oxide (NO) plasmatic levels	(232, 233)
Metabolic disease	Diabetes	Glucose, insulin, uric acid, glycated albumin	(186, 234)
	Hyperlipidemia	Total cholesterol (TC), low-density lipoprotein cholesterol, high-density lipoprotein cholesterol, apolipoprotein B-100	(186)
	Hyperkalemia	Potassium, albumin	(235)
	Lactic acidosis/hyperlactatemia	Lactate	(236)
	Gout/Hyperuricemia	Uric acid, interleukin-6	(193)
	Nonalcoholic fatty liver disease	TC, insulin	(237)
Neurodegenerative disease	Parkinson’s disease	alpha-Synuclein (α-Syn), CCL2, DJ-1	(214)
	Alzheimer’s disease	Combination of lipocaline-1, lysozyme-C, lacritin, and dermcidin	(217)
	Multiple sclerosis	alpha-1 antichymotrypsin	(220)
Others	Cancer	Complement C1q subcomponent subunit C, protein S100A8, aldehyde dehydrogenase 3A, triosephosphate isomerase	(228)
	AIDS	IgA	(238)
	Concussion	Eye movement	(239)

## 4 Technologies Overview

Overall, wearable medical devices designed for the eye, such as SCLs, use a range of technologies to collect and analyze health data related to the eye. Wearable devices are equipped with electronic parts and wireless communication technology, and various materials and fabrication technologies are applied. Since different elements are required depending on the desired function and target disease, it is necessary to be able to apply an appropriate method.

### 4.1 Materials

**Figure 7:**
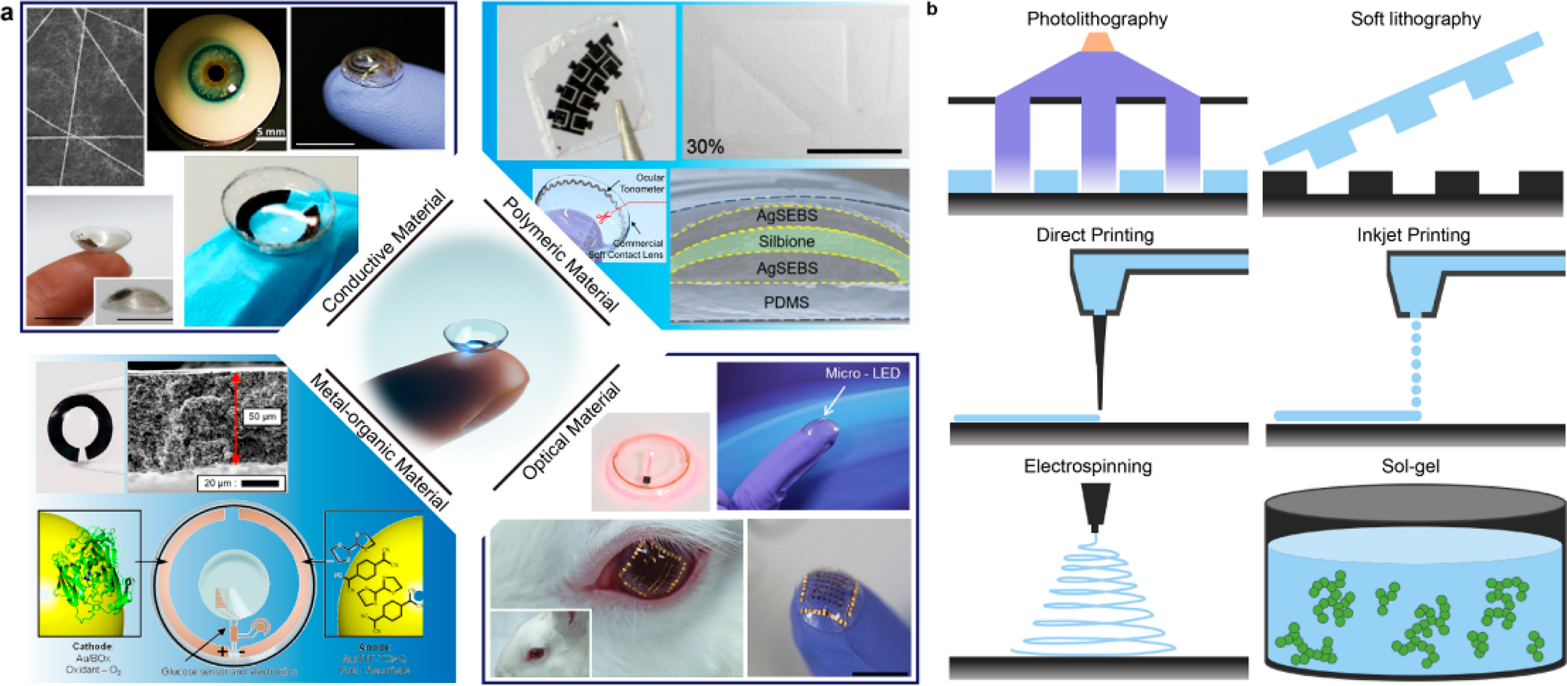
Preparation of SCLs: materials and fabrication. (a) Types of materials used to fabricate SCLs. Reproduced with permission from ref (240). Copyright 2021 American Association for the Advancement of Science under CC BY 4.0 (https://creativecommons.org/licenses/by/4.0/). Reproduced with permission from ref (241). Copyright 2020 American Chemical Society. Reproduced with permission from ref (242). Copyright 2019 American Association for the Advancement of Science under CC BY 4.0 (https://creativecommons.org/licenses/by/4.0/). Reproduced with permission from ref (243). Copyright 2020 American Association for the Advancement of Science under CC BY-NC 4.0 (https://creativecommons.org/licenses/by-nc/4.0/). Reproduced with permission from ref (244). Copyright 2022 American Chemical Society. Reproduced with permission from ref (245). Copyright 2020 American Association for the Advancement of Science under CC BY-NC 4.0 (https://creativecommons.org/licenses/by-nc/4.0/). Reproduced with permission from ref (246). Copyright 2018 American Association for the Advancement of Science under CC BY-NC 4.0 (https://creativecommons.org/licenses/by-nc/4.0/). Reproduced with permission from ref (247). Copyright 2022 Springer Nature. Reproduced with permission from ref (248). Copyright 2021 American Chemical Society. Reproduced with permission from ref (249). Copyright 2013 American Chemical Society. Reproduced with permission from ref (250). Copyright 2022 John Wiley and Sons under CC BY 4.0 (https://creativecommons.org/licenses/by/4.0/). Reproduced with permission from ref (251). Copyright 2017 American Chemical Society. Reproduced with permission from ref (252). Copyright 2021 John Wiley and Sons. (b) The schematic illustration of various SCL fabrication methods.

The choice of materials for an SCL depends on several factors, including their intended purpose (such as sensing or drug delivery), and the intrinsic material properties that are compatible with the design and application of the SCL. For example, for biosensing of the different properties of the eye, different materials are chosen to enhance the efficiency and accuracy of the sensing material. Other materials are chosen to address the structural requirements needed for a functional SCL, such as biocompatibility, permeability, and durability. Additionally, some materials can be designed to respond to external stimuli, such as light or electromagnetic fields, to enable advanced functionality. We have introduced some of the unique materials chosen for the fabrication of an SCL and their intended applications below (Figure 7a).^239−251^

#### 4.1.1 Conductive Materials

Conductive materials are an essential component of an SCL as they enable the sensing, transmission of data, and protection from electromagnetic waves. In addition to these functions, conductive materials also act as interconnections for different components of the lens. In the case of a conductive material used for an SCL, it must have excellent electrical properties, and in addition, physical and chemical stability.^253,254^ Also, in order to apply it to the soft contact lens, stretchability is required.^255,256^ Some examples of conductive materials used in an SCL include graphene,^251,256−258^ carbon nanotubes,^244^ metal nanowires,^240,259−262^ and metal nanoparticles.^263−265^ These materials can be used to create electrodes and sensors that can detect changes in electrical potential or conductivity.

Lee et al. demonstrates a graphene-based contact lens platform that protects eyes from electromagnetic waves that can cause eye diseases such as cataracts, while also reducing dehydration. The sheet resistance of the graphene is low even in a wet environment, and the EM wave shielding function of the graphene-coated lens was tested successfully on egg whites exposed to strong EM waves, suggesting potential for future wearable technologies in healthcare and bionics.

Kang et al. report on the development of an SCL with embedded glucose fuel cells that can provide stable power for microelectronic devices.^244^ The researchers optimized the fuel cell components using carbon nanotubes, resulting in a maximum power density of 4.4 μW cm^–2^ and maintained performance even after bending the lenses in half 100 times. The fuel cells were also able to distinguish between tear glucose levels under normal and diabetic conditions.

Kim et al. have developed an SCL with an IOP sensor, flexible drug delivery system, wireless power and communication, and an IC chip for monitoring and controlling IOP in glaucoma.^259^ The contact lens uses a gold hollow nanowire-based sensor and a flexible drug delivery system to provide on-demand drug delivery, and it could serve as a futuristic healthcare platform for glaucoma and other ocular diseases.

Jeon et al. have introduced a camera-based optical monitoring system (OMS) using nanoparticle-embedded contact lenses that changes color according to the level of glucose in tears without the need for complicated electronics.^263^ An image processing algorithm has been proposed that optimizes measurement accuracy even with image blurring, and tests on mice and human tear samples show that the OMS accurately measures glucose concentration and has potential to simplify glucose monitoring for diabetes patients.

#### 4.1.2 Polymeric Materials

Polymeric materials play a critical role in the fabrication of an SCL, serving as the base structural support and matrix for drug delivery. The selection of polymeric materials is based on several factors, including biocompatibility, mechanical stability, and the ability to release drugs. Some examples of polymeric materials used in SCLs include hydrogels,^266^ chitosan,^266,261^ and polymers^245,246^ (Table 5).^34,267−270^ In addition to serving as a drug delivery matrix, polymeric materials also act as a barrier to protect the eye from harmful substances, and they can be designed to have specific properties such as permeability or electrical conductivity. Furthermore, polymeric materials can be functionalized with various functional groups to enhance the sensing capabilities of the contact lens. Overall, the properties of the chosen polymeric materials must be compatible with the design and application of the SCL to ensure that it functions properly and is safe for use.

**Table 5:** Other Materials for SCL

Material	Molecular formula	Advantages	Disadvantages	Reference
PMMA	(C_5_H_8_O_2_)_n_	High optical transparency, low cost, easy fabrication	Low oxygen permeability, high toughness,	(34)
PET	(C_10_H_8_O_4_)_n_	Good chemical and heat resistance, easy to shape	Hydrophobic surface	(266)
PHEMA	(C_6_H_10_O_3_)_n_	Great optical transparency, gas permeability, biocompatibility	No antimicrobial properties	(267)
PDMS	(C_2_H_6_OSi)_n_	Good transparency, flexibility, air-permeability, biocompatibility	Hydrophobic surface	(268)
MPC	C_11_H_22_NO_6_P	Good protein adsorption resistance, cell adhesion, blood coagulation	Mechanical weakness	(269)

Kim et al. reported SCLs for continuous glucose monitoring (CGM) have huge clinical potential, but their development has been limited by challenges in accurately detecting glucose levels without hysteresis.^270^ However, using bimetallic nanocatalysts immobilized in nanoporous hydrogels, a long-term robust CGM has been demonstrated in diabetic rabbits with high sensitivity, fast response time, low detection limit, and low hysteresis, showing promise for future clinical applications.

Mehta et al. used electrohydrodynamic atomization to engineer novel coatings for ocular contact lenses capable of releasing timolol maleate (TM) for the treatment of glaucoma.^271^ The approach utilized chitosan, borneol, polyvinylpyrrolidone, and poly(*N*-isopropylacrylamide) to create highly stable nanomatrices with advantageous morphology and size, and it showed biphasic and triphasic release depending on the composition, with high TM encapsulation and excellent ocular biocompatibility, offering an alternative dosage form to improve patient compliance.

Zhang et al. reports a class of smart soft contact lenses that can continuously monitor IOP, even during sleep, to aid in glaucoma care.^247^ The lenses are built upon various commercial brands of soft contact lenses without altering their intrinsic properties, and they have been shown to accurately measure IOP under ambulatory conditions.

#### 4.1.3 Metal–Organic Materials

Metal–organic materials are hybrid materials that contain both metal and organic components. In SCL fabrication, these materials are used as building blocks to create functional materials for sensing, drug delivery, and energy storage applications.^272,273^ For example, metal–organic frameworks and covalent organic frameworks can be used as porous materials for drug delivery, gas storage, and separation applications. These materials can also be used as coatings for SCL to enhance their sensing capabilities or to provide protection against environmental factors. Additionally, metal–organic complexes can be used as electrocatalysts for energy storage applications, such as in rechargeable batteries for the contact lens.

Yun et al. have developed flexible, aqueous batteries that can safely power an SCL, using nanocomposite flexible electrodes of carbon nanotubes and Prussian blue analogue nanoparticles embedded in an UV-polymerized hydrogel.^274^ The battery has a discharging capacity of 155 μAh in an aqueous electrolyte with an ionic concentration equivalent to tears and is mechanically stable, biocompatible, and compatible with contact lens cleaning solutions, providing a safe power supply without risk of injury due to battery leakage or breakage.

Falk et al. developed a microscale biofuel cell (BFC) that can generate electrical energy from human tears, using the ascorbate and oxygen naturally present in tears as fuel and oxidant.^249^ The biodevice features three-dimensional nanostructured gold electrodes modified with abiotic and biological materials functioning as efficient anodic and cathodic catalysts, respectively, and could be a potential power source for glucose-sensing contact lenses used in continuous health monitoring for diabetes patients.

#### 4.1.4 Optical Materials

Optical materials are essential components of SCLs, allowing for the detection and modulation of light. Light-emitting diodes (LEDs) and perovskite nanocrystals are among the optical materials commonly used in SCLs.^250,274^ These materials enable the creation of various optical components, such as polarizers, filters, and displays, which can enhance and modify visual perception. Additionally, LEDs can have therapeutic applications in SCLs. Optical materials can also facilitate the measurement of physiological parameters,^275^ through the incorporation of optical sensors that detect changes in light absorption or emission.^276^

Lee et al. reported a noninvasive smart wireless contact lens has been developed for the treatment of diabetic retinopathy with significantly improved compliance.^250^ The contact lens contains a far red/NIR light emitting diode that reduces retinal vascular hyper-permeability induced by diabetic retinopathy in rabbits by simply repeated wearing of the lens for 8 weeks with 120 μW light irradiation for 15 min thrice a week.

Jang et al. presented a novel method for transferring perovskite patterns to planar or nonplanar surfaces using a removable polymer, which enables the formation of a perovskite image sensor array on a soft contact lens.^252^*In vivo* tests with rabbits showed the wearability of the contact lens, and the transfer method also demonstrated the formation of a multiplexed sensing platform detecting light and tactile pressure simultaneously.

In the field of perovskite-based SCLs, remarkably, researchers commonly utilize biocompatible polymer coatings, like parylene-C, to safeguard against direct exposure of the perovskite to the eyes. The application of this protective coating serves to enhance safety by mitigating the potential risks associated with perovskite exposure, thereby ensuring a higher level of safety and reducing any associated hazards.

### 4.2 Fabrication Technologies

Fabrication technologies in SCLs refer to the diverse techniques utilized to manufacture and assemble the different functional components of the lens. The importance of fabrication technologies in SCLs lies in their capability to produce lenses with precise and intricate structures that are necessary for the integration of various functional components. Advanced fabrication technologies enable mass production of SCLs at lower costs, thereby making them more accessible. In addition, these technologies can be utilized to create personalized SCLs customized to individuals’ specific needs, including visual correction or monitoring of health parameters. Therefore, we will introduce some of the common fabrication methods utilized in SCLs (Figure 7b).

#### 4.2.1 Photolithography

Photolithography is an extensively used fabrication technique in SCLs, and it enables the creation of precise patterns and structures on the surface of a lens.^247,259^ The process involves transferring a pattern onto a light-sensitive substrate using light, followed by removing the soluble areas with a chemical developer.

The significance of photolithography lies in its ability to create intricate and precise patterns for integrating various functional components into the lens, such as sensors and displays, while maintaining transparency and thickness for optimal vision. Moreover, photolithography facilitates the mass production of SCLs at a lower cost, making them more accessible to a broader range of individuals.

Despite its advantages, photolithography has several limitations in SCL fabrication. The primary constraint is that it is a planar process, and this restricts the complexity and functionality of the lens to two-dimensional structures. Moreover, the technique requires expensive equipment and specialized skills, which limits its accessibility for small-scale production or individualized customization. In addition, the use of chemical developers may limit the use of functional materials for more intricate functionality.

#### 4.2.2 Soft Lithography

Soft lithography is a microfabrication technique used in SCL manufacturing to create precise patterns and intricate structures on the surfaces of a lens.^143,245^ The technique involves using elastomeric stamps to transfer a pattern onto the substrate. The importance of soft lithography in SCLs lies in its ability to create complex 3D structures with high resolution and accuracy. It is a versatile and cost-effective technique that allows for the integration of various functional components, such as sensors and displays, into the lens. In addition, soft lithography enables the use of functional materials, such as hydrogels and conductive polymers, which may be challenging to incorporate using other fabrication techniques.

However, one limitation of soft lithography is that it can be difficult to achieve high-resolution patterns with fine features, particularly when compared to techniques such as photolithography. In addition, the flexibility of the soft materials used in soft lithography can lead to deformation or instability in the final product, potentially affecting the functionality of the SCL. Finally, the cost and complexity of soft lithography may limit its accessibility for small-scale production or individualized customization, similar to other fabrication techniques.

Using soft lithography, microfluidic components that manipulate fluids in channels with dimensions on the micrometer scale can be fabricated. These components can be used for various applications, including chemical and biological analysis, the delivery of drugs, and sensing. In the context of SCLs, microfluidics can be used to create microchannels and reservoirs for drug delivery or to integrate sensors for the real-time monitoring of various physiological parameters.

One example of microfluidics in SCLs is the development of a glucose biosensor for the noninvasive monitoring of blood glucose levels in diabetic patients. The biosensor is integrated into the contact lens, and it consists of a microfluidic channel that contains an enzyme-based glucose sensor. The channel is designed to allow for the flow of tear fluid over the sensor, which can detect glucose levels in real-time. The biosensor can transmit the glucose level data wirelessly to a mobile device or other monitoring system. Another example is the use of microfluidics to create drug delivery systems (DDSs) that can be integrated into the contact lens. Microfluidic channels can be used to deliver drugs to the eye in a controlled and sustained manner, avoiding the need for frequent application of eye drops. The drug can be stored in a reservoir in the contact lens and released through the microchannels in response to stimuli, such as pH or temperature changes.

In addition, microfluidic devices can be used to create micropumps that can generate fluid flow within the contact lens. This can be used to enhance the oxygen permeability of the lens, reduce the risk of dry eye syndrome, and improve comfort for the wearer. Overall, microfluidics offers a promising approach for the development of SCLs with advanced functionalities. However, the integration of microfluidic devices into contact lenses requires careful design and optimization to ensure the safety, biocompatibility, and performance of the device.

#### 4.2.3 Printing

Direct printing and inkjet printing are two popular printing methods used in various applications, such as bioelectronics and SCL manufacturing.^242,277−280^ Direct printing involves depositing a material directly onto a substrate in a controlled pattern, which can be categorized into two types, i.e., contact printing and noncontact printing. In contact printing, the printing material is transferred to the substrate by direct contact, while in noncontact printing, the material is deposited onto the substrate without physical contact. However, Inkjet printing uses a printhead with tiny nozzles to deposit droplets of ink or other materials onto a substrate with high precision, allowing for the creation of fine features and intricate patterns. Inkjet printing can be used to deposit various materials, including conductive inks, insulators, organic semiconductors, and biomolecules. Additionally, the utilization of 3D printing methods for SCL manufacturing has been extensively studied in that they can build 3D structures for electronic components depending on the required functions. For example, Kim et al. introduced a high-resolution 3D printing of liquid metal to form stretchable free-standing 3D patterns of interconnects essential to electrically connect individual device components for their integration.^32^ Also, Park et al. developed a 3D printing method for fabricating a wirelessly chargeable 3D solid-state supercapacitor that serves as a power source for SCLs.^236^

Using these methods, SCLs can be fabricated by depositing sensors, electronics, conductive materials, drug-loaded nanoparticles, and protective coatings onto the lens. The flexibility and precision offered by these methods enable the creation of custom molds and components on different substrates, including flexible and stretchable materials. However, to ensure safety and biocompatibility for the wearer, careful selection of printing materials and optimization of printing parameters are crucial. Inkjet printing, in particular, has several advantages over traditional printing methods, such as greater flexibility and customization, lower cost, and higher throughput. It also can be used to print on various substrates, making it ideal for manufacturing electronics and devices with unconventional form factors.

Another printing technique is electrohydrodynamic (EHD) engineering, which is used in SCL fabrication technology that involves the manipulation of fluids and particles using electric fields. This technique enables the precise control of the size, shape, and position of materials within the contact lens, allowing for the integration of various components, such as sensors, electronics, and DDSs.^281−283^ EHD engineering also can be used to create surface textures and patterns that improve the performance and functionality of the contact lens.

#### 4.2.4 Electrospinning

Electrospinning is a powerful fabrication technique that uses an electric field to produce ultrafine fibers from nanoparticles, nanorods, nanowires, nanotubes, and nanosheets into a polymer solution or melt.^284−288^ The process involves applying a high voltage to a liquid polymer solution or melt, which creates an electrostatic field that pulls the polymer into thin fibers. The fibers are then collected onto a substrate to form a nonwoven mat of nanofibers with high surface area and porosity. Recently, metal-based nanowires, nanofibers, and their hybrid structures have been extensively researched in SCLs for their enhanced electrical, mechanical, and optical characteristics, such as silver nanowire (AgNW), silver nanofiber (AgNF), and their hybrid structures. Kim et al. introduced graphene-AgNW hybrid electrodes for FET sensors that detect glucose levels with enhanced electrical and mechanical properties.^284^ Park et al. presented AgNF-based antenna and interconnects for stretchable and transparent electrodes with high transparency and low haze.^240^ Jang et al. showed AgNF-AgNW hybrid antenna, exhibiting resistance of 55 Ω and inductance of 0.7 μH.^234^

This technique has significant potential for the manufacturing of SCLs, where electrospun nanofibers can be integrated onto the lens surface using techniques such as spin coating or inkjet printing.^246,289−293^ One application of electrospinning is the fabrication of drug-loaded nanofibers, which can be incorporated into the contact lens material to provide sustained drug release for the treatment of eye diseases such as glaucoma. The porous structure of the electrospun fibers allows for a high drug-loading capacity and controlled release kinetics.

Moreover, electrospinning can also be used to produce nanofiber-based sensors that can be integrated into the contact lens for real-time monitoring of various physiological parameters. For example, nanofiber sensors can be designed to detect glucose levels in tears, which can be useful for patients with diabetes. The high surface area and porosity of electrospun fibers allow for a high sensitivity and selectivity of the sensors.

In addition, electrospinning can be used to create porous and breathable membranes that can enhance the oxygen permeability and comfort of the contact lens. By improving the oxygen supply to the cornea, the use of electrospun membranes can reduce the risk of corneal hypoxia and increase the wearer’s comfort.

#### 4.2.5 Sol–Gel

Sol–gel is a versatile and widely used material synthesis technique that involves the conversion of a colloidal suspension (sol) into a solid network (gel).^289^ It has potential applications in the manufacturing of SCLs.^288,289^ Sol–gel can be used to create thin films or coatings on the surface of the lens to impart various functional properties, such as antifog, antireflective, and scratch-resistant properties. These coatings can be tailored to the specific needs of the wearer, such as improving vision clarity or reducing glare in bright light conditions. Sol–gel can also be used to encapsulate drugs or other bioactive molecules within the lens material, enabling sustained release over an extended period of time. This can be particularly useful in the treatment of chronic eye diseases, such as glaucoma or dry eye syndrome. Additionally, sol–gel can be used to create hybrid materials that combine the advantages of inorganic and organic materials, such as improved mechanical strength and optical properties. However, careful selection of sol–gel precursors and optimization of processing conditions are necessary to ensure the safety and biocompatibility of the contact lens for the wearer.

### 4.3 Powering

A continuous power supply is required to drive an SCL, but it has been challenging to integrate a bulky battery in the SCL due to its limited area. To overcome this limitation, various methods of supplying power to the SCL have been developed in different ways.^294^

The powering source used in SCLs must adhere to several requirements. It should be flexible, lightweight, and not generate heat because of its direct contact with the cornea. It should be able to operate for a long time to enable real-time or continuous monitoring. Above all, it must be made of biocompatible materials, devoid of toxic substances that could pose a risk of harming the eyes.^295,296^ Based on the prevailing problems, it is not practical to employ traditional batteries that use electrolytes, and the current powering approach utilizing wearable devices has restrictions when it is inserted into the small area of a contact lens, measuring between 14.2 and 14.5 mm in diameter.^297^ Therefore, innovative powering protocols are necessary, and this section explains the principle of a new wireless power method used in SCLs.

#### 4.3.1 Wireless Power Transmission

Wireless power transmission (WPT) is a technology that enables the wireless transfer of power by utilizing electromagnetic waves generated from an external transmitter antenna.^298^ The power is then transmitted to electronic devices and sensors. In this technology, a power transmitter, such as a smartphone or glasses, is located outside the lens, while an antenna capable of receiving electromagnetic waves from the transmitter is present on the lens. The technology uses various frequencies ranging from MHz to GHz and diverse antenna formats. Due to its wireless and compact value, WPT is a popular method in the field of lens technology.^299^

**Figure 8:**
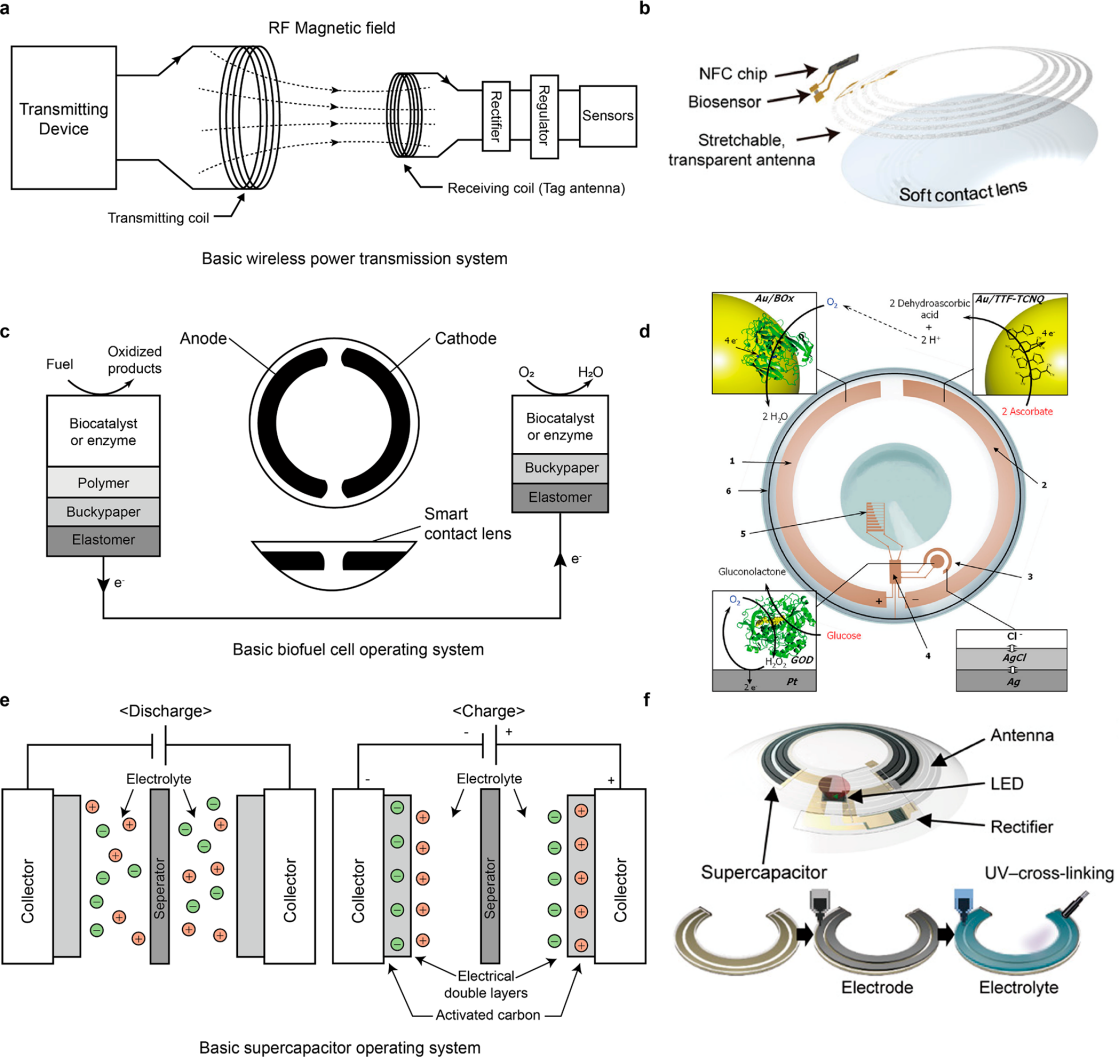
Principles and examples of various power supply methods of SCLs. (a) Schematic of the WPT system of the two antennas. (b) SCL powered by the WPT method using a transparent antenna. Reproduced with permission from ref (240). Copyright 2021 The American Association for the Advancement of Science under CC BY 4.0 (https://creativecommons.org/licenses/by/4.0/). (c) Schematic of the BFC battery system that drives the SCL. (d) SCL with a built-in BFC battery system that measures glucose concentration in tears. Reproduced with permission from ref (249). Copyright 2013 American Chemical Society. (e) Schematic of the discharging and charging system of supercapacitor. (f) SCL powered by supercapacitor for LED. Reproduced with permission from ref (242). Copyright 2021 The American Association for the Advancement of Science under CC BY 4.0 (https://creativecommons.org/licenses/by/4.0/).

WPT is divided into radio frequency identification (RFID) and near field communication (NFC) according to frequency. NFC is a technology for wireless communication at a very short distance within 10 cm with a band of 13.56 MHz of radio frequency (RF).^300^ RFID uses up to a frequency of GHz, and it is possible to communicate over a long distance. In order to meet the conditions of maintaining the acquisition of biosignals for extended periods using low RF power, it is imperative to minimize the consumption of power. The power absorbed by the RFID tag chip among the power transmitted by the corresponding reader can be expressed as follows:^301^
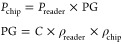
where *C* represents the antenna coupling coefficient, and PG represents the energy transmission obtained by the RFID tag chip from the reader. The impedance matching coefficients at the reader and the tag are denoted as ρ_reader_ and ρ_chip_, respectively. The energy transfer between the reader and the antenna with rectifier and regulator can be modeled as a two-port network, as illustrated in Figure 8a. Among the various variables, the relative positions of the power transmitter and receiver can impact power consumption. This is because coupling with the antenna in the near field affects not only the impedance, but also the distribution of the electrical or magnetic field. During such instances, this coupling is similar to the principle of an inductively coupled transformer, and *C* can be expressed as follows:^301^
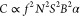
where *f* is the frequency, *N* is the number of coil turns, *S* is the overlapped cross-section area of the coils, *B* is the magnetic field at the tag location created by the reader antenna, and α is the misalignment loss. In summary, the communication range is proportional to the operating frequency. Depending on the desired range, either NFC or RFID can be selected and utilized. The shape of the antenna shape utilizes a ring-type geometry, as depicted in Figure 8b, to avoid obstructing the line of sight.^240^ Jang et al. fabricated an antenna using transparent AgNFs to minimize visual obstruction. The antenna, made with AgNFs, exhibited high transparency of over 70% in the visible wavelength range. In addition, the wireless powering systems for implantable devices, including eye contact devices, heart pacemakers, and nerve stimulators, necessitate meticulous consideration of various factors to ensure optimal performance and safety. These factors include receiver size, WPT distance, power transfer efficiency, and tissue safety. Given the restricted area of each receiver antenna, achieving high power transfer efficiency according to the WPT distance is crucial. For example, eye-related devices, which operate within relatively short WPT distances, commonly use NFC-based power transfer methods. On the other hand, deep implantable devices, such as brain stimulators, heart pacemakers, and nerve stimulators require power transfer efficiency to be maximized while ensuring tissue safety through the regulation of frequency parameters.

#### 4.3.2 Biofuel Cells

A biofuel cell (BFC) typically consists of an anode and a cathode, with power being supplied through an electrochemical reaction across the electrolyte between the anode and cathode.^249^
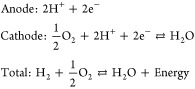


BFCs operate according to the same general principles as fuel cells. Electric energy is generated through the flow of electric current that occurs when electrons are reduced at the anode and oxidized at the cathode. Unlike WPT, BFCs do not require external equipment and can autonomously generate power for a limited period. BFCs can be divided into enzymatic fuel cells (EFCs), microbial fuel cells (MFCs), and organelle-based BFCs. Among BFCs, EFC is emerging as a power source for a new SCL because it has a high potential to obtain enzymes from tears and does not require the use of external materials.^302^

EFCs are BFCs that use an enzyme as a catalyst when oxidizing. Most fuel cells use metals such as platinum and nickel as catalysts, but EFCs use enzymes derived from living cells. The BFC in the contact lens uses biomolecular species such as glucose and lactate in tear fluid to generate electrical energy by causing an electrochemical redox reaction.^249^ As shown in Figure 8c, the reduction reaction of the enzyme occurs at the anode and the current flow, where the oxidation reaction occurs at the cathode, is connected to the biosensor part. Falk et al. showed that it can be driven by using 3D nanostructured gold-wire-supported glucose/O_2_ EBFC as a power source for SCL, and as a follow-up study, bilirubin oxidase (BOx) confirmed to reduce the oxygen concentration of actual tears -based biocathode was used to drive the lens.^249^ As shown in Figure 8d, after supplying power through the EFC of the anode and cathode, the change in glucose concentration was measured in the biosensor. However, EFC-based biofuel cells have a limited lifespan and cannot be used for a long time as its property that easily lose their activity due to environmental changes such as external shocks, temperature, and pH.

#### 4.3.3 Supercapacitor

A supercapacitor is a high-capacity capacitor with a much higher capacitance value than other capacitors, but it has a lower voltage limit. It stores the physical energy of ions that draw electricity and store it. An electrolyte is present between the anode and the cathode, and an active electrode for adsorption and desorption of ions is combined with the electrode.^295^ When a voltage is applied between the two electrodes, positive ions are attracted to the negative electrode, negative ions are attracted to the positive electrode, and energy is stored, as depicted in Figure 8e. As ions from the electrolyte solution diffuse into the pores of an electrode with an opposite charge, they accumulate at the interface between the electrode and the electrolyte, resulting in the formation of a large electrical double layer. The capacitance value (*C*) of this double layer is proportional to the surface area (*A*) of the electrode-electrolyte interface and can be expressed as the reciprocal of the distance (*d*) between the two layers.^303,304^
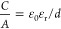
where ε_r_ represents the dielectric constant, ε_0_ is the constant of the permittivity of free space and *d* represents the thickness of the double layer for a given surface area, *A*. The thickness of the double layer is influenced by the concentration of electrolytes and the size of the ions. The total energy density (*E*) of a supercapacitor when voltage of *V* is applied is
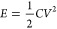


In an SCL, supercapacitors can be made of nontoxic materials and have long cycling stability and high power density, making them attractive as power supplies.^294,305^ Furthermore, when combined with a solar cell or WPT, they have the advantage of enabling a 24-h cycle that can be charged. Park et al. introduced WPT to address the shortcoming of supercapacitors, which have a limited operating time for long-term sensing. As shown in Figure 8f, the SCL in their study consists of an antenna, supercapacitor, LED, and rectifier, and they confirmed that the lens could effectively perform tasks such as heat and LED driving through the supercapacitor.^242^

Besides the WPT, BFC, and supercapacitors, various battery sources, such as solar cells and flexible batteries, are being researched for their potential application in SCL (Table 6).^306−310^ Developing a self-sustainable and nontoxic power source that can withstand the high elasticity required to respond to the curvature of the eye presents an opportunity to advance 24-h biosensing monitoring and establish a dependable biosensing platform. Currently, silicon-based solar cells utilized for powering SCLs use ambient light to change solar energy into electrical energy.^306^ When photons from sunlight are absorbed by the semiconductor material, electron-hole pairs are created. Owing to the presence of a built-in electric field, electron-hole pairs are effectively separated from the charged electrodes. By connecting an external circuit, the flow of electrons powers the SCLs. During daytime, solar cells harvest energy from ambient light, simultaneously providing power to SCLs. At night, harvested energy at storage device delivers constant power for continuous operation of SCLs. In this way, solar cells-based SCLs enables 24-h continuous monitoring. Another energy generator used in SCLs is flexible batteries. These batteries, designed to be lightweight and conformable, provide a compact and flexible power source for the devices embedded in SCLs. Commonly, flexible batteries are composed of flexible electrodes (anode and cathode), electrolytes, separator, and encapsulation. In recent studies, due to the fatal danger of a liquid electrolyte, solid-state electrolytes-based flexible batteries are used, ensuring biocompatibility of SCLs.^307^ However, for the long-term operation, additional advancements are essential to improve the energy storage capacity and longevity of flexible batteries for SCLs.

**Table 6:** Powering Systems

	Power or energy density	Operation lifetime	Advantage/Disadvantage	Reference
WPT	1 μW cm^–2^	–	High output power, heating issue	(307)
BFC	3.5 μW cm^–2^	20 h	Fuels from body, limited lifetime	(308)
Supercapacitor	333 μWh cm^–2^	80% after 10000 cycles	Cycling stability, high energy density, lower energy density	(309)
Solar cells	1.24 μW cm^–2^	–	Power generation from ambient light, stability issue	(310)
Flexible battery	71.42 μWh cm^–2^	11.7 h	High energy density, limited operation lifetime	(306)

### 4.4 Data Transmission

As described previously, the SCLs employ ICs and biosensors to conduct biosensing wireless data communication from tears. These innovative systems allow for the wireless transmission of data to electronic devices, such as smartphones, and facilitate continuous monitoring of the health condition.^239^ It is imperative that these lenses facilitate real-time measurement while ensuring optimal comfort for the wearer. There are currently both wired and wireless methods for receiving data from the SCL. However, it is generally recommended to use a wireless data transmission method due to its convenience and compactness. It eliminates the need for physical connectors and wires, which can reduce complexity and improve flexibility in certain applications. Therefore, the wireless method is employed for data transmission method.

#### 4.4.1 Various Types of Wireless Data Transmission

**Figure 9:**
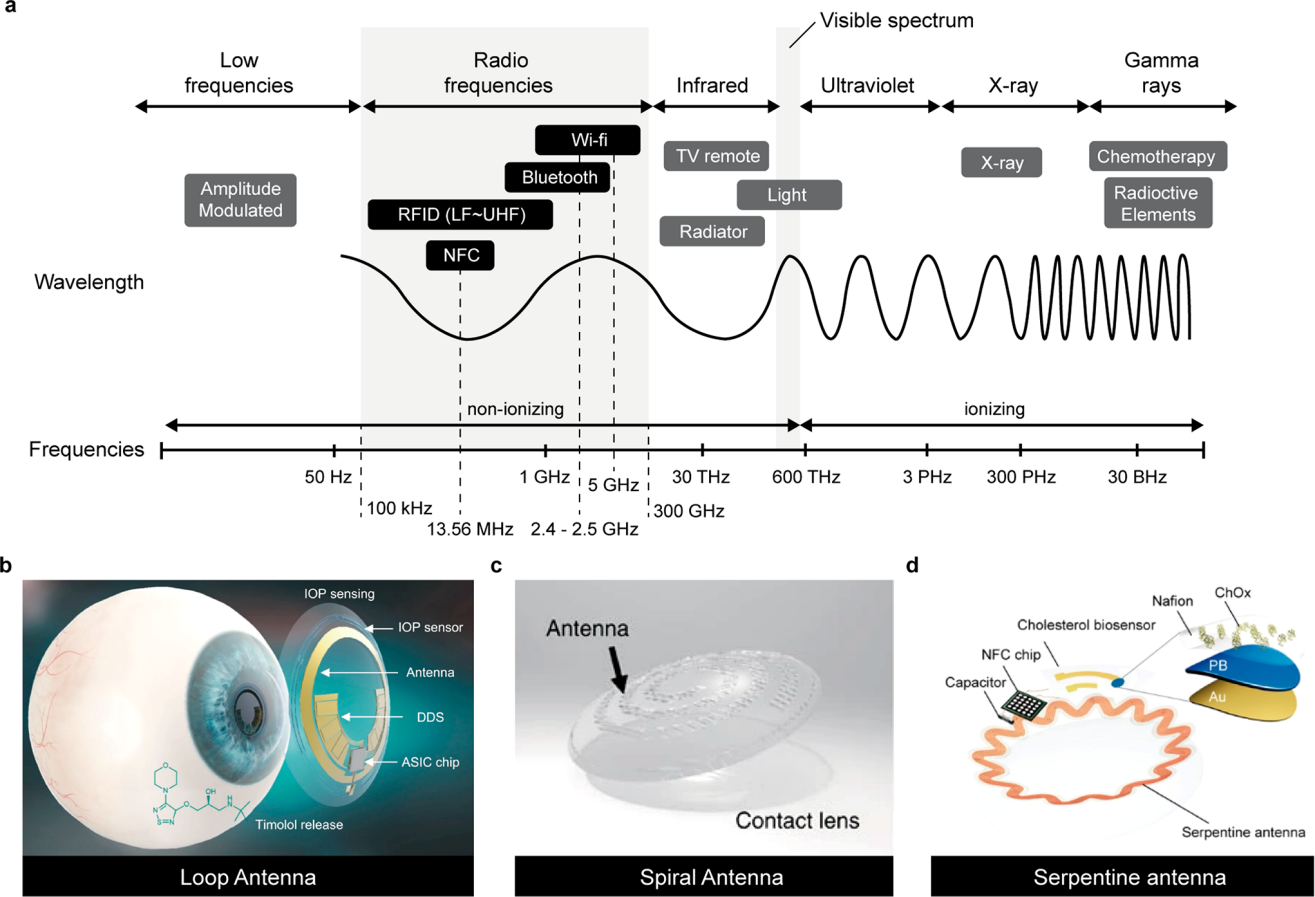
SCL wireless data transmission method. (a) Types of data transmission applications according to the frequency of electromagnetic waves. (b) SCL using a loop-shaped antenna. Reproduced with permission from ref (259). Copyright 2022 Springer Nature under CC BY 4.0 (https://creativecommons.org/licenses/by/4.0/). (c) SCL using a spiral-shaped antenna. Reproduced with permission from ref (320). Copyright 2017 Springer Nature under CC BY 4.0 (https://creativecommons.org/licenses/by/4.0/). (d) SCL using a serpentine-shaped antenna. Reproduced with permission from ref (203). Copyright 2021 Wiley under CC BY 4.0 (https://creativecommons.org/licenses/by/4.0/).

The WPT method is a technology that employs various radio spectra to transmit analog or digital signals through the atmosphere.^311^ Wireless data communication applications can transmit signals ranging from 30 Hz to 300 GHz, and they are categorized based on their frequency width and usage. The frequency utilized in medical devices is the industrial, scientific, and medical (ISM) frequency, which operates within the range of 6.7 MHz to 244 GHz. The WPT method for transmitting data involves the use of Wireless Fidelity (Wi-Fi) and Bluetooth, which operate at frequencies of 2.4 and 5 GHz, respectively. In addition, RFID uses a frequency range from kHz to MHz, and can be classified into four types based on the frequency that is used. These include low frequency identification (LFID) that operates at 125 or 134 kHz and can function within a range of 10 cm, and high frequency identification (HFID) and ultrahigh frequency identification (UFID) that operate in the 860 to 960 MHz band and can identify objects up to 15 m away. NFC uses a frequency of 13.56 MHz with 10 cm distance (Figure 9a).^312^ Even though Bluetooth and Wi-Fi use similar frequencies, they differ in purpose and function. Bluetooth has low bandwidth as it communicates with only a few specific devices, whereas Wi-Fi has high bandwidth. Bandwidth refers to the amount of information that can pass through a data connection in a given amount of time. Likewise, NFC uses a frequency of 13.56 MHz, which also is used by RFID. However, unlike RFID, NFC allows for two-way communication and can transmit data only within a short distance of 0.1 m. The table summarizing the WPT data transmission method is as follows (Table 7).^311,313−317^

**Table 7:** Types of Wireless Data Transmission

	NFC	RFID	Bluetooth	Wi-Fi
Network type	Point-to-point	Point-to-point	Wireless Personal Area Network (WPAN)	Wireless Local Area Network (WLAN)
Set-up time	<0.1 s	<0.1 s	<6 s	<6 s
Frequency	13.56 MHz	LF/HF/UHF/Microwave	2.4–2.5 GHz	2.4–2.5 GHz, 5 GHz
Distance	<0.1 m	∼100 m	∼10 m	∼100 m
Continuous sampling	No	No	Yes	Yes
Power consumption	Low	Varies with frequency	Medium	High

In terms of energy efficiency and wireless power transfer capabilities, NFC is the optimal technology for use in electronic contact lenses. This is because utilizing higher frequencies results in greater heat generation and places a larger burden on the body due to the use of RF, making high frequencies unsuitable for wireless data communication. However, when it comes to transmitting large amounts of data at high rates, midfield RFID systems based on low GHz bands, which can effectively manage power loss, may be more suitable for data communication.^317^

#### 4.4.2 SCL Data Transmission

SCL utilizes a built-in antenna for the transmission of WPT data. The antenna integrated in SCL functions as a receiver and captures electromagnetic waves emitted by the transmission terminal of an external device.^318^ In addition, the antenna for data transmission acts not only as a receiver but also as a transmitter to transfer the data (detected by biosensors) to an external device. The most common method of recognizing data involves connecting an antenna to a biosensor and associating biosensing information with characteristic changes, such as alterations in antenna inductance and capacitance.^319^ The resonance frequency of the antenna is impacted by changes in the resistance or capacitance of the sensor, leading to a related reflection value.^320^ Various types of antennas, including loop antennas, spiral antennas, and those made with nanomaterials, such as graphene and hybrid silver nanofibers, are utilized for this purpose.^321^ Especially, antennas are recognized as a key element in the sensing of IOP, where they are utilized in conjunction with capacitive sensors to identify changes in frequency that result from pressure.

##### 4.4.2.1 Loop Antenna

The loop-shaped antenna is a widely used antenna design that avoids the obstruction of vision. It can be connected easily to the sensing circuit at the beginning and end of a single loop, resulting in a compact structure. The shape of the loop can be varied, and an inner semicircle design, for example, can increase the parasitic inductance and capacitance of the antenna, resulting in a reduction in frequency.^322^ Kim et al. conducted IOP sensing using an SCL integrated with the loop-shaped antenna shown in Figure 9b. The lens is capable of monitoring IOP levels to prevent glaucoma and facilitate drug delivery based on the IOP measurements. The authors have developed a theragnostic SCL that integrates a wireless power and communication system to monitor the IOP of glaucoma patients.^259^

##### 4.4.2.2 Spiral Antenna

Kim et al. developed an SCL utilizing a spiral-shaped antenna. The researchers used the antenna shown in Figure 9c to reflect the resistance change of the glucose sensor and alter the reflection value of the antenna for data transmission.^320^ In addition, they measured the change in resonance frequency due to the alteration in capacitance with varying IOP, finding that the resonance frequency of the antenna, which resonated at 4.1 GHz, increased as the IOP decreased. The researchers utilized a network analyzer and impedance analyzer to receive the two-sensing information of the corresponding lens.^320^ The antenna was manufactured by forming hybrid nanostructures between silver nanowires (AgNWs) and graphene, and it showed an optical transmittance of over 80% and a high transmittance of less than 10% for haze spectra at a wavelength of 400 nm.

##### 4.4.2.3 Serpentine Antenna

Soontornpipit et al. compared a spiral antenna with an antenna featuring a serpentine structure. The comparison showed that the spiral antenna had a strong coupling response only at the center of the antenna, whereas the serpentine antenna showed a coupling response with adjacent arms as well. As a result, the serpentine structured antenna, which can maintain a closer electrical distance, has a higher resonant frequency than a spiral antenna with the same physical length, resulting in higher efficiency.^323^ Song et al. fabricated an SCL utilizing an antenna with serpentine geometry, as shown in Figure 9d. Although this antenna is geometrically serpentine, it has only the basic characteristics of a spiral antenna. Here, instead of the above-mentioned case for the purpose of increasing efficiency, a curved structure was used to achieve high stretchability through structural transformation. The SCL is capable of measuring cholesterol levels from tears and is combined with a capacitor and NFC chip.^203^

Therefore, WPT data transmission of the smart contact can be adjusted by changing the shape and size of the antenna and combined with biosensing to link changes in capacitance or inductance. Also, by utilizing various nanomaterials, the physical properties can be altered to produce an SCL with diverse characteristics, such as LED and transparency. To ensure high stability and a wide field of vision in the future, the size of the antenna will have to be reduced. One promising method to achieve this is by integrating an RFID circuit and antenna into the SCL. As explained in the Powering section, the RFID method allows for operation by receiving energy and converting the acquired sensor information into a bit-stream method to store and receive the information when near the receiver.^324,325^ The wireless data transmission method of the SCL needs to be updated to a method that can operate for 24 h, maintain body stability, and enable further reception in the future.

## 5 Diagnostic SCLs

Disease diagnostic SCLs have emerged as a new generation of wearable biosensor platforms that can monitor various physiological and chemical parameters of disease in real-time, noninvasively, and in a continuous manner. In terms of the operating principle, SCLs for disease diagnosis can be categorized into four main groups: electrochemical, physical, electrophysiological, and optical sensing systems. The method of measuring the concentration of chemical biomarkers using electrochemical reactions was classified as an electrochemical diagnosis. In addition, diagnostic SCLs for measuring physical biomarkers and electrophysiological signals were classified into physical diagnosis and electrophysiological diagnosis, respectively. In the case of optical diagnosis, although it is based on physical or chemical principles, it is classified separately because the information is finally obtained as an optical signal.

### 5.1 Electrochemical Diagnosis

Most biosensors utilize an electrochemical detection method for the transducer due to its cost-effectiveness, ease of use, and simple construction. This method typically involves monitoring a reaction electrochemically, resulting in a measurable current (i.e., amperometry), charge accumulation or potential (i.e., potentiometry), or changes in the conductive properties of the medium between electrodes (i.e., conductometry).^326^ In electrochemical sensors, a biological sensing material (e.g., enzyme, protein, antibody) selectively reacts with the target analytes, producing an output domain. Then this output domain is converted into the electrical domain by the electrochemical transducer.^327,328^ The electrochemical method is not heavily dependent on the volume of the reaction, and this allows the use of very small sample volumes during measurements.^329^

#### 5.1.1 Electrochemical Sensing System in SCL

The electrochemical sensing mechanism of SCLs involves the detection of changes in electrochemical properties, such as current, voltage, or resistance, caused by the reaction between the target analytes and the sensing materials.^330^ Electrochemical sensing methods that are used often in SCLs are resistive type and amperometric biosensors.

**Figure 10:**
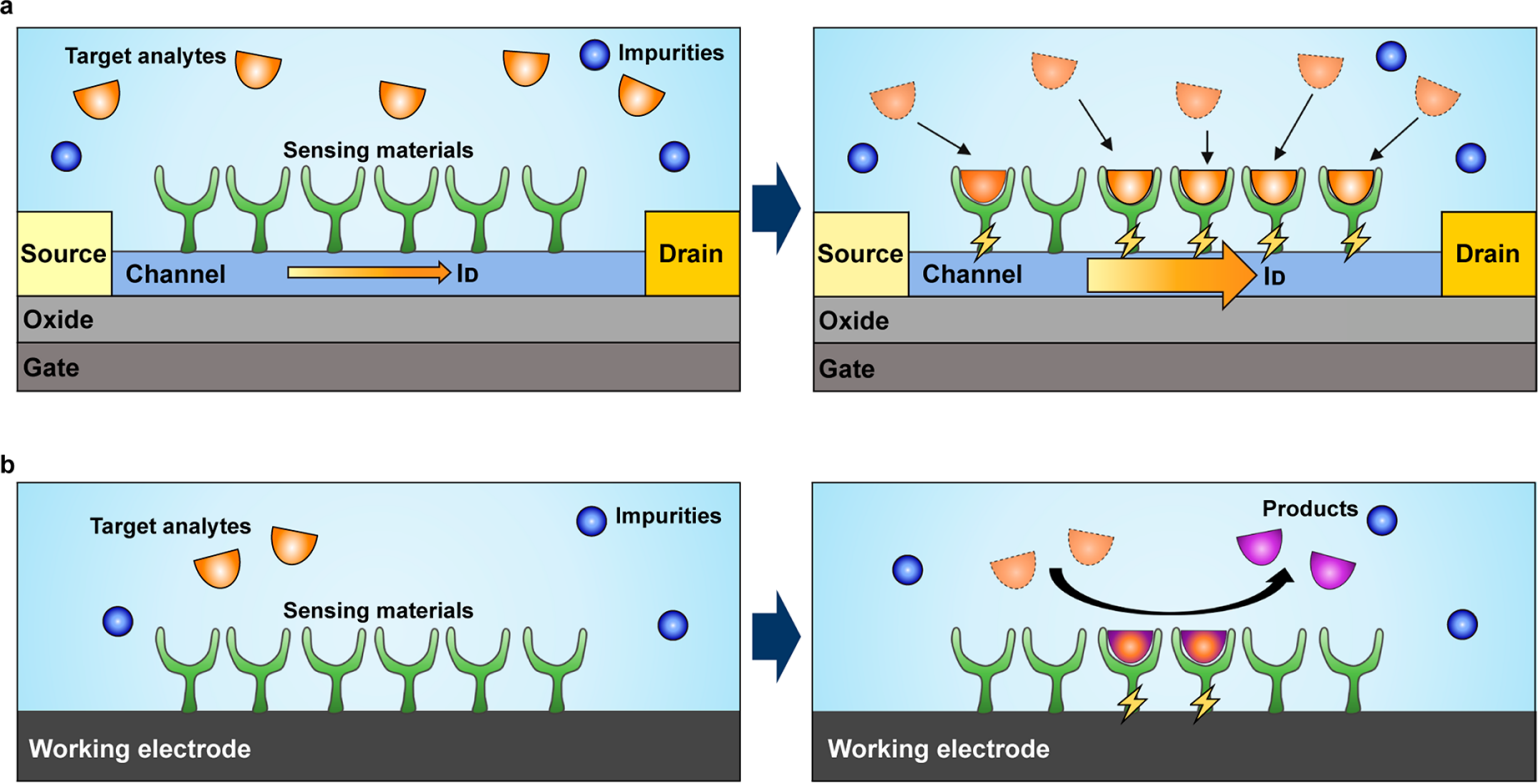
Electrochemical sensing systems. (a) Schematic illustration of field-effect transistor (FET)-based sensor. Before (left) and after (right) the reaction of the target analytes and the sensing materials. *I*_D_: Drain current. (b) Schematic illustration of the amperometry sensor. Before (left) and after (right) the reaction of the target analytes and the sensing materials.

The resistive type biosensor is based on the three electrode structures of the FET, such as the source, drain, and gate electrodes.^331^ The conducting channel is formed between the source and drain electrodes, and the gate is separated from this channel by a thin oxide layer (Figure 10a). This type of sensor operates based on the principle of changing the electrical properties of a sensing layer that is formed in the channel region. When a voltage is applied between the source and drain electrodes (drain voltage, *V*_D_), a drain current (*I*_D_) flows through the conducting channel.^332^ The sensing materials, which are immobilized on the channel, control the conductivity of this channel by modulating the gate voltage and serve as the sensing element. The electrical properties of the FET can be altered to signal when the target analytes attach to the surface of the sensing materials on the channel. This interaction of the target analytes and sensing materials modifies the surface charge and leads to a change in the gate voltage. Furthermore, this change can be detected by measuring the degree of modulation of *I*_D_ and the concentration of the target analytes.

An amperometric biosensor is a sensing method that is composed of a working electrode (WE), a reference electrode (RE), or a counter electrode (CE).^330^ Like the channel in the resistive type biosensors, the WE typically is immobilized with sensing materials that react with the target analytes (Figure 10b). For example, when glucose oxidase (GOx) is immobilized on the WE, the glucose reacts to GOx. For this reaction, a constant oxidation or reduction potential between the WE and the RE is applied to the WE. The potential can be adjusted to optimize the sensitivity and selectivity of the biosensor for the target analytes. As the concentration of the target analyte changes, the sensing material undergoes a reaction that generates a change in the current level through the WE. Therefore, measuring this change in this electrical property can be calculated to the signal of the concentration of the target analytes. Electrochemical diagnostic methods are effective diagnostic methods in that they provide high sensitivity and rapid screening of various diseases.^333^ Currently, research is being conducted to develop noninvasive and continuous monitoring of diseases through SCL devices.

#### 5.1.2 Resistive Type

In the case of diabetes, blood glucose levels must be checked several times a day with the finger-prick method. Noninvasive SCLs can be an effective diagnostic method that replaces the painful finger-prick method. Therefore, SCLs for glucose are currently being actively studied as described below.

**Figure 11:**
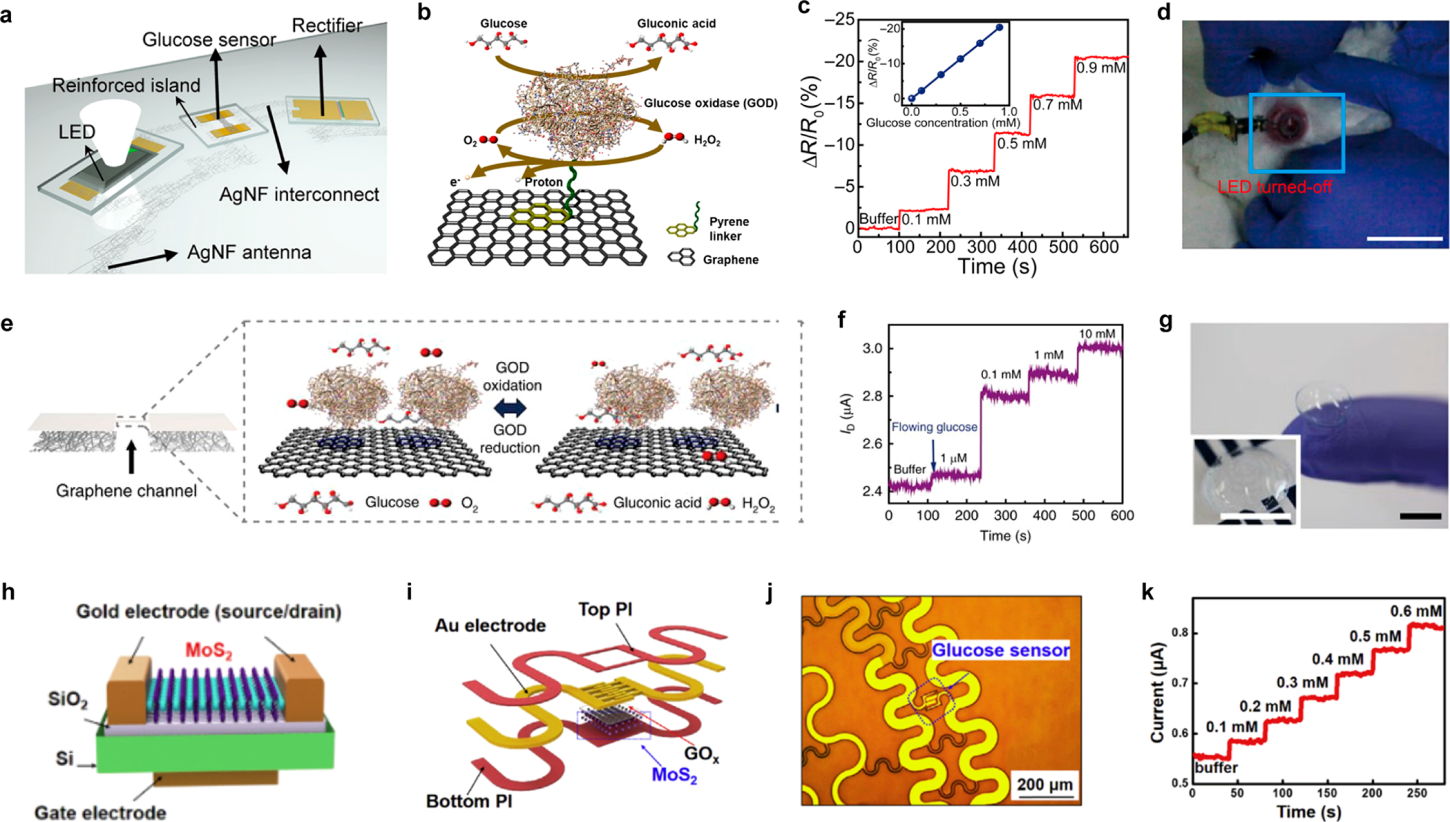
SCLs using resistive type sensor for glucose measurement. (a) Schematic illustration of the SCL consists of the rectifier, the LED, the glucose sensor on reinforced island, and the silver nanofiber (AgNF) antenna/interconnect. (b) Schematic illustration of glucose sensing mechanism on graphene channel. c, The real-time electrical response of the glucose sensor in the range of 0.1 to 0.9 mM glucose levels. Inset, Calibration curve of the glucose sensor. (d) Photograph of a rabbit wearing SCL for glucose measurement. Scale bar, 1 cm. (a–d) Reproduced with permission from ref (247). Copyright 2018 Park et al. under CC BY-NC 4.0 (https://creativecommons.org/licenses/by-nc/4.0/). (e) Schematic illustration of glucose sensing mechanism on graphene channel in resistive type sensor. (f) The real-time electrical response of the glucose sensor in the range of 0.01 to 10 mM glucose levels. (g) Photograph of the SCL. Inset, close-up image. Scale bars, 1 cm. (e–g) Reproduced with permission from ref (320). Copyright 2017 Kim et al. under CC BY 4.0 (https://creativecommons.org/licenses/by/4.0/). (h) Schematic illustration of the MoS_2_ FET device on the SiO_2_/Si substrate. (i) Schematic illustration of the serpentine MoS_2_ photodetector. (j) Photograph of the MoS_2_ glucose sensor. Scale bar, 200 μm. (k) The real-time electrical response of the MoS_2_ glucose sensor in the range of 0.1 to 0.6 mM glucose levels. (h–k) Reproduced with permission from ref (338). Copyright 2021 Elsevier.

Park’s group developed an SCL with a resistive type glucose sensor, stretchable AgNF antenna, and LED display (Figure 11a).^247^ The fabricated SCL exhibited soft and transparent properties that provide a clear view without the inconvenience of having to wear it. To enable selective and sensitive glucose detection, they immobilized GOx on the graphene surface using a pyrene linker through π–π stacking interaction (Figure 11b).^334^

When glucose was present on this sensor, the GOx oxidized the glucose, producing hydrogen peroxide (H_2_O_2_). The sensor works by allowing glucose to pass through the graphene channel where it undergoes oxidation by GOx. The reaction produces a byproduct of H_2_O_2_, which is further decomposed to oxygen, protons, and electrons.^335^ The positive charge transfer effect of the graphene channel (p-type) is caused by the protons. As the density of the major carrier is proportional to the concentration of glucose, the sensor detects the relative change in resistance (Δ*R*/*R*_0_) as a function of glucose concentration, and this led to an increase in the I_D_ of the FET.^336^ The sensing test of the fabricated glucose biosensor was conducted in a glucose solution, the concentration of which ranged from 0.1 to 0.9 mM (Figure 11c). The response time of this sensor was measured as ∼1.3 s. The signal-to-noise ratio (SNR) was measured to be 23.87 at a concentration of 0.1 mM. Using an SNR threshold of ∼3, the minimum detectable concentration was estimated to be approximately 12.57 μM. This SCL is operated by wirelessly receiving RF signals through the antenna, which then are converted into direct current (DC) by the rectifier. The LED and glucose sensor were powered by this DC. When the glucose sensor detected a glucose concentration above the threshold level, the resistance of the sensor decreased, causing a reduction in the bias applied to the LED pixel, which could turn it off. Moreover, the reliable operation of the device was demonstrated through *in vivo* tests conducted on a live rabbit, with no noticeable adverse effects (Figure 11d). Furthermore, Park’s group presented another SCL that was capable of simultaneously diagnosing IOP and glucose levels.^320^ For glucose monitoring, an FET sensor was fabricated with a graphene-AgNW hybrid, and GOx was immobilized on the graphene channel (Figure 11e). Similar to the above-mentioned research regarding resistive type biosensors, the oxidation of glucose to gluconic acid and the reduction of water to H_2_O_2_, which are facilitated by GOx, result in an increase in the concentration of charge carriers in the channel. As a consequence, the ID changes as the concentration of glucose increases. The drain current was measured across a range of glucose concentrations from 1 μM to 10 mM at zero gate bias (*V*_G_ = 0 V) (Figure 11f). At 1 μM, the SNR was found to be 7.34. The limit of detection, defined as the concentration at which the SNR was 3, was determined to be 0.4 μM. Notably, the sensor exhibited a strong response to glucose concentrations in the range of 0.1 to 0.6 mM, which is the typical range of glucose concentrations found in human tear fluids.^337^ With the advantages of the graphene-AgNW hybrid structure, a stretchable and transparent SCL was fabricated (Figure 11g). Guo et al. developed an SCL capable of real-time monitoring of glucose levels based on the MoS_2_-FET glucose sensor.^338^ The donut-shaped sensor layer of the lens consisted of a MoS_2_-FET glucose sensor, a MoS_2_-FET photodetector, and a thin Au temperature sensor connected by serpentine metal electrodes. Using a standard nanofabrication process, MoS_2_-FET with a basic back-gate configuration was fabricated directly on a SiO_2_/Si substrate, without any surface functionalization or dielectric coating process (Figure 11h). The MoS_2_-FET glucose sensor component featured interdigital source/drain electrodes with an optimized channel length of 30 μm and immobilized GOx for glucose sensing (Figure 11i). The facile fabrication process of the integrated ultrathin MoS_2_ transistor and the gold wire-based sensor system allowed for high detection sensitivity with direct eye contact (Figure 11j). Similar to the mechanism of a general FET sensor, the charge transfer process in the sensor occurs as a reaction in which glucose is oxidized by GOx, resulting in the production of H_2_O_2_. Then, the H_2_O_2_ reacts with oxygen to generate electrons and hydrogen ions. The free electrons contribute to the increase in device’s current due to the n-type FET behavior. The real-time response of glucose concentration ranging from 0.1 to 0.6 mM is demonstrated in Figure 11k, indicating a high level of sensitivity proportional to the glucose levels (|*R*|/*R*_0_). For example, at a glucose concentration of 0.6 mM, the sensitivity was 48%.

In addition to measuring the glucose levels in tears, the measurement of other molecules, such as enzymes, hormones, and metabolites using the SCL platform, also has become a promising field.

**Figure 12:**
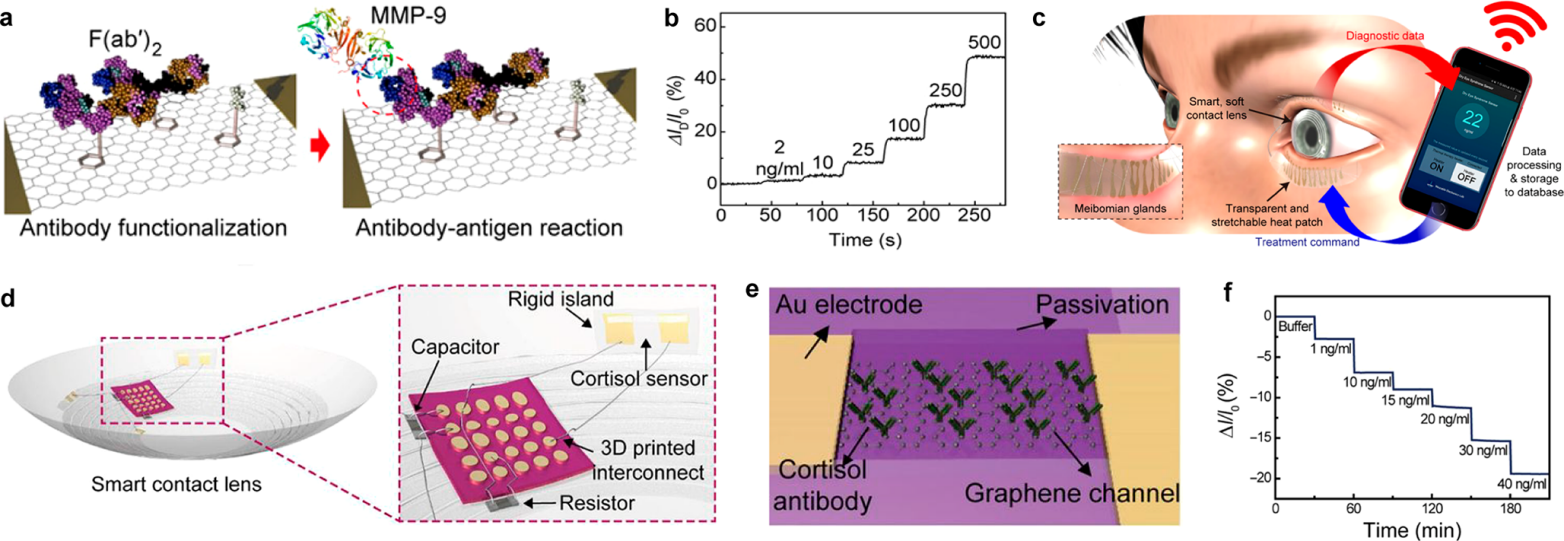
SCL using resistive sensor for other molecules. (a) Schematic illustration of matrix metalloproteinase-9 (MMP-9) sensing mechanism by Fab functionalization on graphene channel. (b) The real-time electrical response of the MMP-9 sensor in the range of 2 to 500 ng mL^–1^ MMP-9 levels. (c) Schematic illustration of the transmission system with the SCL and a smartphone. (a–c) Reproduced with permission from ref (240). Copyright 2021 Jang et al. under CC BY-NC 4.0 (https://creativecommons.org/licenses/by-nc/4.0/). (d) Schematic illustration of the integrated SCL with a capacitor, resistor, and cortisol sensor on rigid island by liquid metal interconnect. (e) Schematic illustration of the graphene FET sensor for cortisol detection. (f) The real-time electrical response of the graphene FET sensor in the range of 1 to 40 ng mL^–1^ cortisol levels. (d–f) Reproduced with permission from ref (243). Copyright 2020 Ku et al. under CC BY-NC 4.0 (https://creativecommons.org/licenses/by-nc/4.0/).

For diagnosis of chronic ocular surface inflammation (OSI), such as DES, matrix metalloproteinase-9 (MMP-9) was utilized.^339,340^ The conventional diagnostic methods for chronic OSI, such as evaluating epithelial abnormalities, tear film quality, and the degree of conjunctival injection, were found to have issues with accuracy in their diagnostic criteria, leading to potential misdiagnoses.^341,342^ MMP-9 is a crucial endopeptidase used for pathological analysis, and it has been utilized as a biomarker in the diagnosis of chronic ocular surface disease due to its strong correlation with the inflammatory status of the ocular surface. Jang et al. developed a wireless SCL for monitoring and digitizing chronic OSI levels with a resistive type biosensor.^240^ The system relies on quantitative analysis of MMP-9 (Figure 12a) and provides schematic illustrations of the functionalization process of Fab and the subsequent antigen-antibody reactions. To achieve specific detection of MMP-9, the F(ab′)2 fragment was immobilized onto the graphene channel using a pyrene linker from 1-pyrenebutanoic acid succinimidyl ester through π–π stacking. Then, the succinimidyl ester components were combined with the amino base of the F(ab′)2 fragment. Then, the antigen-binding site could attach to MMP-9. Real-time measurement of I_D_ was conducted at *V*_G_ = 0 V to evaluate the transfer characteristics of the sensor for various MMP-9 concentrations ranging from 1 to 500 ng mL^–1^, as shown in Figure 12b. The sensor exhibited high sensitivity to pathological levels of MMP-9 concentration in tear fluids, ranging from 1 to 500 ng mL^–1^. The sensor achieved an SNR of 8.14 at 2 ng mL^–1^, and the limit of detection (LOD) (at the SNR = 3) was 0.74 ng mL^–1^. The sensitivity of this sensor was calculated to be 11.1 ng mL^–1^ per 1% change in its drain current. The MMP-9 level data in tears measured through the sensor of the SCL is transmitted wirelessly to a smartphone for processing and storage (Figure 12c).

Cortisol is an example of another molecule that can be measured using a resistive type transistor. Cortisol, a steroid hormone that commonly is known as a stress hormone, is secreted by the adrenal gland in response to psychological or physical stress.^343^ However, an abnormal accumulation of cortisol due to its excessive secretion can lead to several severe diseases, such as Cushing’s disease, autoimmune disease, cardiovascular complications, and T2D, as well as neurodegenerative disorders, i.e., depression and anxiety disorders.^224,344^ On the other hand, abnormally low levels of cortisol can result in Addison’s disease, causing hypercholesterolemia, weight loss, and chronic fatigue.^345^ Therefore, developing real-time healthcare systems to monitor cortisol levels has been researched extensively over the past decade as a means of quantifying stress levels.^346,347^ Although cortisol detection systems have been developed for various samples, such as blood, sweat, and interstitial fluid, the accuracy of their measurements often was hindered by biological instability caused by temperature and mechanical stress. With the rapid advancements in smart bioelectronics, tear fluid has emerged as a promising sampling area for cortisol measurement.^50^ Ku et al. presented a wireless SCL device capable of monitoring cortisol levels in tears.^243^ The SCL is composed of a resistive type biosensor, a transparent NFC antenna, and an IC chip (Figure 12d). All of the components were integrated electrically by utilizing a printing technique with liquid metal, which resulted in a fine line width of less than 10 μm. The sensor was developed using a graphene FET that can detect cortisol levels by binding cortisol monoclonal antibodies (C-Mab) to the surface of the graphene (Figure 12e). The immobilization process involves coupling the C-Mab to the carboxyl group of the graphene surface using the EDC/NHS coupling reaction. The sensor was able to detect cortisol concentrations ranging from 1 to 40 ng mL^–1^ in real-time at zero gate bias (*V*_G_ = 0 V) and a *V*_D_ of 0.1 V, with a LOD of 10 pg mL^–1^ at the SNR of 3 (Figure 12f). This LOD is low enough to detect typical cortisol concentrations found in human tears. The sensor showed a linear decrease in current with increasing cortisol concentration, and the sensitivity was calculated to be 1.84 ng mL^–1^ per 1% change in resistance. In addition, the researchers conducted a pilot trial with human subjects and an *in vivo* test on live rabbits, demonstrating the SCL’s good biocompatibility and potential for clinical use.

#### 5.1.3 Amperometric Type

**Figure 13:**
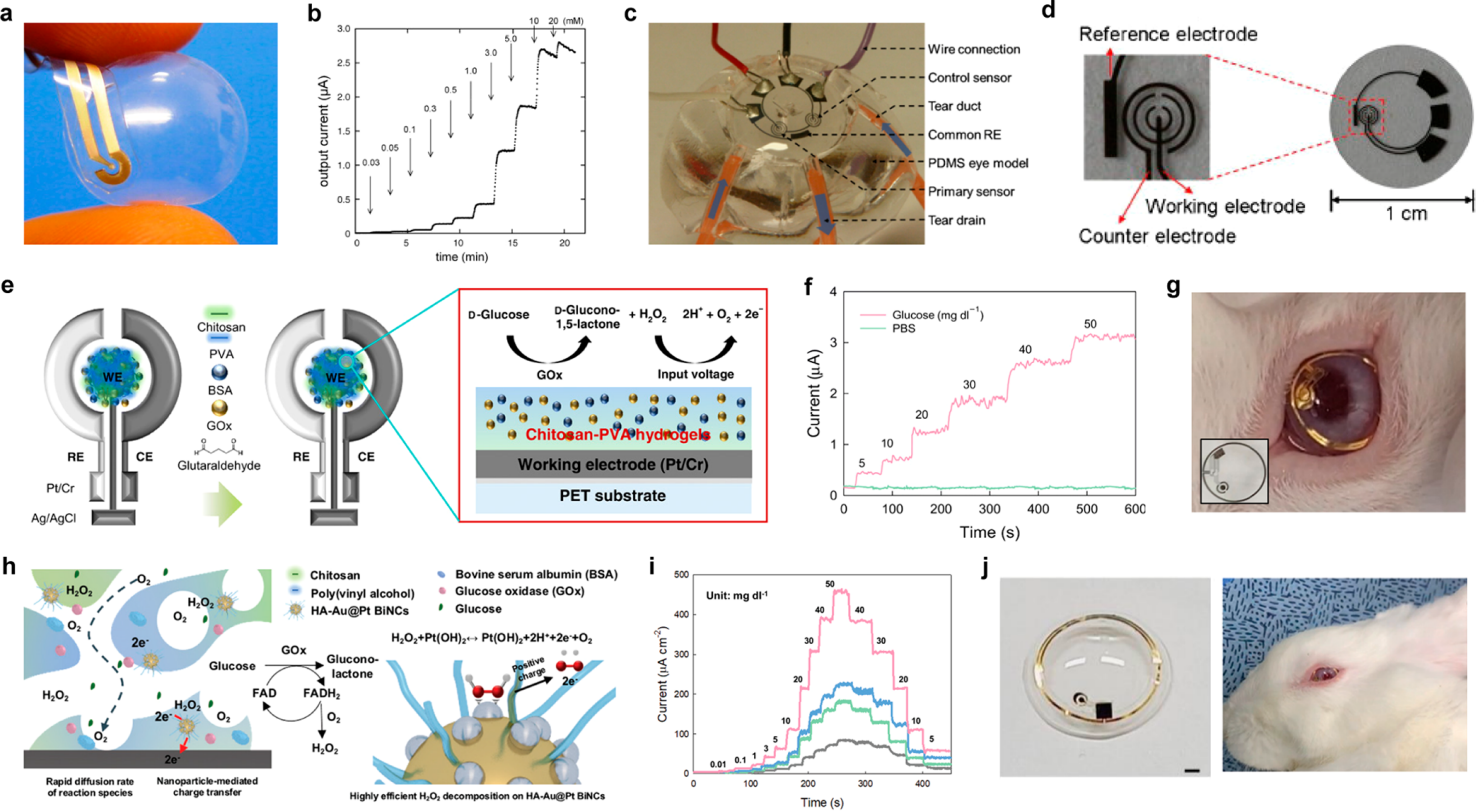
SCLs using amperometric sensor for glucose measurement. (a) Photograph of the amperometric glucose sensor formed on the contact lens. (b) The real-time electrical response of the glucose sensor in the range of 0.03 to 20 mM glucose levels. (a, b) Reproduced with permission from ref (348). Copyright 2011 Elsevier. (c) Photograph of the glucose sensing contact lens using a dual-sensor structure. Reproduced with permission from ref (349). Copyright 2012 IOP Publishing Ltd. (d) Photograph of the amperometric glucose sensor. Scale bar, 1 cm. Reproduced with permission from ref (337). Copyright 2011 Elsevier. (e) Schematic illustration of the glucose sensor with working electrode (WE), reference electrode (RE), and counter electrode (CE) (left) and sensing mechanism of glucose oxidase in Chitosan-poly(vinyl alcohol) hydrogel (right). (f) The real-time electrical response of the glucose sensor in the range of 5 to 50 mg dL^–1^ glucose levels (pink) and in the PBS solution (green). (g) Photograph of a rabbit wearing the integrated SCL. (e–g) Reproduced with permission from ref (245). Copyright 2020 Keum et al. under CC BY-NC 4.0 (https://creativecommons.org/licenses/by-nc/4.0/). (h) Schematic illustration of the glucose sensing mechanism of bimetallic nanocatalysts in nanoporous hydrogels. (i) The real-time electrical response of the bimetallic nanocatalysts sensor in the range of 0.01 to 50 mg dL^–1^ glucose levels (pink). (j) Photograph of the SCL with the bimetallic nanocatalysts sensor (left) and a rabbit wearing the SCL (right). Scale bar, 150 μm. (h–j) Reproduced with permission from ref (270). Copyright 2022 John Wiley and Sons.

An amperometric sensor for glucose measurement utilizing the enzymatic reaction mediated by GOx has also been proposed. Chu et al. developed an electrochemical glucose sensor integrated into a contact lens by affixing the sensor onto the outer surface of the lens Figure 13a.^348^ The sensor utilized an amperometric sensor consisting of a Pt WE on a flexible polydimethylsiloxane (PDMS) substrate. The researchers immobilized GOx on the sensing electrode using a solution made by mixing 2-methacryloyloxyethyl phosphorylcholine (MPC) and 2-ethylhexyl methacrylate (EHMA) at a ratio of 3:7. Furthermore, they coated this solution over the enzyme to prevent its leakage during sensing. The sensor performance evaluation test confirmed the effective sensing of glucose concentrations in the range of 0.03–5.0 mM (Figure 13b). *In vivo* testing showed that the sensor could detect 0.49 mM glucose when 50 μL of 0.5 mM glucose was introduced into the eye of a rabbit wearing the contact lens. Parviz’s group presented a glucose sensing contact lens using a dual-sensor structure that can accurately detect glucose without being affected by other chemical species (Figure 13c).^349^ The dual sensor consists of two identical single sensors, a primary sensor, and a control sensor, both of which were fabricated using Ti/Pd/Pt electrodes. Two types of enzymes, an active GOx and a deactivated GOx, were applied to the primary sensor and control sensor, respectively. By measuring the noise current value using the control sensor, any interference caused by other chemical species can be corrected in the current value measured by the primary sensor, allowing for accurate glucose sensing. The researchers demonstrated that the glucose concentration was measured linearly in a test solution containing three proteins (lysozyme, albumin, and mucin). Parviz’s group also fabricated a sensor that had three circular electrodes made of Ti/Pd/Pt (10/10/100 nm) arranged in concentric rings, as shown in Figure 13d.^335^ The electrodes were connected to wires after the film was molded into a contact lens. The sensor was coated with a GOx/titania sol–gel membrane, and a Nafion solution was added. To measure the concentration of the glucose, solutions ranging from 0.1 to 0.6 mM were used, and the current level increased linearly with the concentration of glucose. The response time was less than 20 s, and the sensitivity was 240 μA cm^–2^ mm^–2^. The detection limit was 0.01 mM in the absence of interfering species, which was ten times smaller than the minimum glucose concentration in human tears. The sensor was stored in a buffer solution at 4 °C and maintained its performance for a week. Hahn’s group developed an SCL with two different types of amperometric sensors. The first contact lens was capable of detecting glucose levels and delivering drugs for the treatment of diabetic retinopathy.^245^ The glucose sensor of this SCL has three electrodes with low chemical resistance that were incorporated to ensure an effective electrochemical glucose reaction. The WE and CE were made of Pt to ensure optimal efficiency. The RE was coated with an Ag/AgCl mixture to achieve accurate amperometric readings in the electrochemical glucose sensor. The WE of the sensor was coated with a mixture of GOx, bovine serum albumin (BSA) and poly(vinyl alcohol) (PVA) (Figure 13e). To evaluate the performance of the sensor, the current was measured while the glucose concentration was varied from 5 to 50 mg dL^–1^ (Figure 13f), and a linear relationship was observed. In addition, the researchers conducted successful *in vivo* experiments on diabetic rabbits, using the sensor to monitor real-time glucose levels (Figure 13g). The other SCL used a reversible glucose monitoring system using nanoporous hydrogels in the SCL (Figure 13h).^270^ The contact lens contained the glucose biosensor using the nanoporous hydrogel that contained bimetallic nanocatalysts (BiNCs) of Au and Pt decorated with hyaluronic acid (HA-Au@Pt BiNCs) and GOx. The presence of nanopores in the hydrogel and HA-Au@Pt BiNCs enhanced the sensitivity (180.18 μA cm^–2^ mmol^–1^), the response time (3.6 s), and limit of detection (LOD) (0.01 mg dL^–1^) by facilitating the diffusion and improving the decomposition efficiency of H_2_O_2_ (Figure 13i). The fabricated SCL was verified through live diabetic rabbits and was validated successfully (Figure 13j).

**Figure 14:**
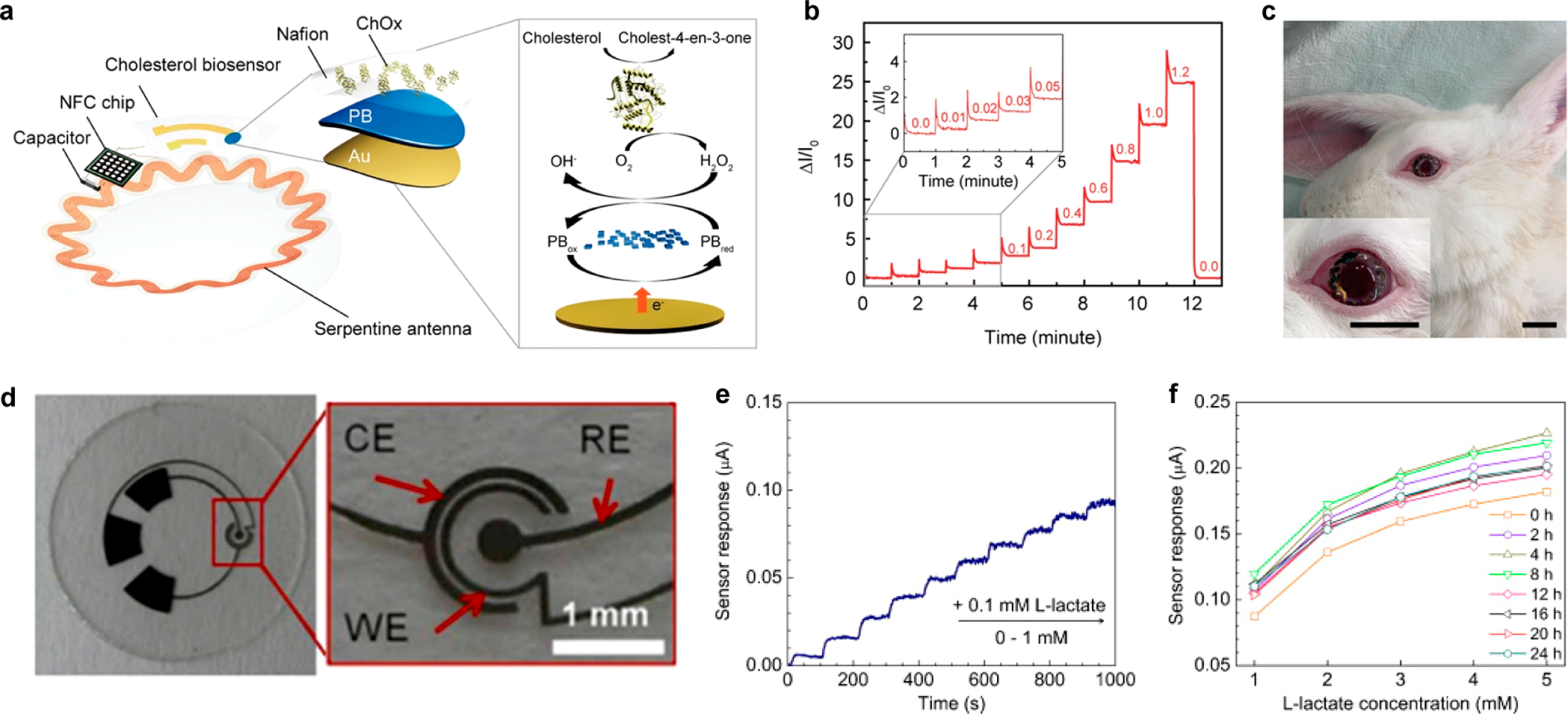
SCLs using amperometric sensor for other molecules. (a) Schematic illustration of the SCL for cholesterol detection (left) and a sensing mechanism of the cholesterol sensor (right). (b) The real-time electrical response of the cholesterol sensor in the range of 0 to 1.2 mM cholesterol levels. (c) Photographs of a rabbit wearing the SCL for cholesterol measurement. Scale bars, 2 cm. (a–c) Reproduced with permission from ref (203). Copyright 2022 John Wiley and Sons under CC BY 4.0 (https://creativecommons.org/licenses/by/4.0/). (d) Photographs of the SCL for l-lactate detection (left) and the l-lactate sensor with working electrode (WE), reference electrode (RE) and counter electrode (CE) (right). Scale bar, 1 mm. (e) The real-time electrical response of the l-lactate sensor in the range of 0 to 1 mM. (f) Stability test of the l-lactate sensor over 24 h. (d–f) Reproduced with permission from ref (353). Copyright 2011 Elsevier.

There also are metabolites in tears that can be detected by the amperometric biosensor. Song et al. developed an SCL that can monitor the cholesterol level in tears continuously.^203^ Cholesterol is a type of lipid that is structurally classified as a modified steroid. It plays several important physiological roles, such as serving as a component of cell membranes, a precursor for steroid hormones and vitamin D, and being present in tear fluid.^83,350^ The concentration of cholesterol is maintained consistently in the body through a complex homeostatic mechanism that regulates the production, absorption, and excretion of cholesterol.^351^ Cholesterol levels can be affected significantly by dietary and lifestyle factors, making continuous monitoring of cholesterol levels a crucial aspect of preventive healthcare. The cholesterol-monitoring SCL developed by Song et al. was composed of an electrochemical cholesterol biosensor, a stretchable NFC antenna, and an IC chip (Figure 14a).^203^ The WE of this cholesterol amperometric sensor was composed of cholesterol oxidase (ChOx), Nafion, Prussian blue (PB), and an Au electrode. When free cholesterol reacts with immobilized ChOx, it is oxidized to generate cholest-4-en-3-one and H_2_O_2_. And the H_2_O_2_ that was generated was reduced PB, which acted as an artificial peroxidase.^352^ The amperometric responses of the sensor in the cholesterol-concentrated PBS solution range from 0 to 1.2 mM showed high sensitivity of 1% change in current per 0.043 mM of cholesterol (Figure 14b). The researchers also conducted an *in vivo* test of the SCL using hyperlipidemia-induced rabbits, and the results showed a good correlation between cholesterol levels in tears and blood (Figure 14c). Furthermore, subsequent human experiments have provided evidence of the feasibility of using the SCL for clinical applications.

Lactate is an example of another tear molecule that can be measured using amperometric sensing. Thomas et al. presented an l-lactate biosensor-integrated contact lens platform.^352^l-Lactate is a crucial metabolite in the human body’s anaerobic glycolytic pathway, and its elevated levels may indicate insufficient clearance or excessive production due to oxygen deficiency or metabolic conditions. This can cause an imbalance in the body’s acid–base equilibrium, leading to lactic acidosis. Since tear fluid can provide a more easily accessible sample for lactate measurements, Thomas and co-workers fabricated the tear-targeted sensor. For the selective detection of l-lactate, lactate oxidase (LOx) was used. This amperometric sensor was a three-electrode system, and LOx was immobilized on the sensing area by glutaraldehyde (GTA) and coated with medical-grade and biocompatible PU. The LOx induced the conversion of l-lactate to pyruvate and H_2_O_2_ in the presence of oxygen.^353^ By applying enough electrical potential, it was possible to oxidize H_2_O_2_ at a WE surface made of Pt. The researchers developed the amperometric sensor composed of a Pt WE, an RE, and an auxiliary Pt CE to ensure a stable reference voltage between RE and WE (Figure 14d). The sensor demonstrated a response time of 35 s, good resolution in the physiological range of lactic acid concentration, and an average sensitivity of approximately 53 μA mM^–1^ cm^–2^ for measuring the concentration of L-lactate in tear fluid within the linear range from 0 to 1 mM (Figure 14e). To assess the long-term stability of the sensors, we periodically measured their current response to l-lactate concentrations within the biological range every 2–4 h over 24 h, while storing them in PBS at room temperature between measurements. As shown in Figure 14f, the current response of a single sensor to various l-lactate concentrations remained fully functional throughout the 24 h.

Electrochemical biosensors, which are commonly used in enzyme-based systems, face the challenge of long-term stability. Enzymes, being the basis of these sensors, can exhibit decreased stability and even degradation under the reaction conditions or due to external factors. To address these drawbacks, various strategies have been adopted in enzyme-based biosensors, such as coating the sensor interface with protective layers or immobilizing the enzymes by mixing them with immobilization materials. However, despite these approaches, enzyme-based biosensors still pose challenging issues. Overcoming these challenges requires the development of enzymes for biomarker detection, along with the design of coating materials or immobilization materials capable of significantly enhancing the physical and chemical stability of enzymes. Once these issues are resolved, enzyme-based biosensors can play a broader and more impactful role in diagnostic SCLs.

Furthermore, it is necessary to consider the biocompatibility and safety of the electrochemical biosensor. While most of the components of an SCL are embedded within the soft contact lens, the electrochemical biosensor must possess biocompatibility as it needs to come into direct contact with tears for measurement purposes. As mentioned earlier, enzymes such as GOx, ChOx, immobilization layers like Nafion, and mediators such as Prussian blue are typical components of an electrochemical sensor. It is essential for these components to exhibit biocompatibility. Moreover, the electrochemical reactions and the safety of the byproducts resulting from the enzyme and biomarker interactions are also crucial. The byproducts, including H_2_O_2_, of the reactions introduced here are harmless to the living body and have negligible effects on the eyes because the amount is very small. Indeed, when developing enzymes for diagnosing a wider range of diseases, it is crucial to consider the biocompatibility of not only the enzymes themselves but also the immobilization materials, mediators, and other substances involved in the biosensing process. Additionally, it is important to ensure that the byproducts resulting from the enzymatic reactions are harmless to the body.

### 5.2 Physical Diagnosis

Various advantages of SCL, ranging from continuous usability to invasiveness, enable the adoption of this platform for detecting biomarkers to diagnose specific diseases or to check the condition of patients. Among the biomarkers from an ocular system that can be detected by the SCL platform, physical factors including IOP, temperature, and eye movement will be introduced in this section. In addition, related diseases or features of the ocular system, along with their detection mechanisms, are discussed.

#### 5.2.1 Intraocular Pressure

Monitoring IOP is essential for monitoring the condition of the ocular system and preventing the occurrence of glaucoma. The conventional method for detecting IOP is using tonometry by ophthalmologists. Unfortunately, this method has limitations because patients have to visit the hospital, and data collection is restricted to specific times. Moreover, IOP is affected by individual postures and diurnal variations, and the degree of fluctuation can be different between healthy subjects and patients with glaucoma. Therefore, continuous monitoring of IOP is crucial for diagnosing glaucoma or observing a patient’s condition. This can be accomplished by the adoption of the SCL platform to continuously monitor the IOP of an individual. As the IOP increases, deformations are induced on an SCL, causing the radius of curvature of the cornea to increase. Specifically, a 1 mmHg change of IOP results in about a 3 μm change in the radius of curvature of the cornea in the human ocular system. The degree of this deformation can be correlated to the level of IOP, and various methods have been used to detect how much the deformation develops on an SCL depending on the IOP. In this section, we will discuss SCLs with IOP sensors focusing on detection methods.

##### 5.2.1.1 Piezoresistive

**Figure 15:**
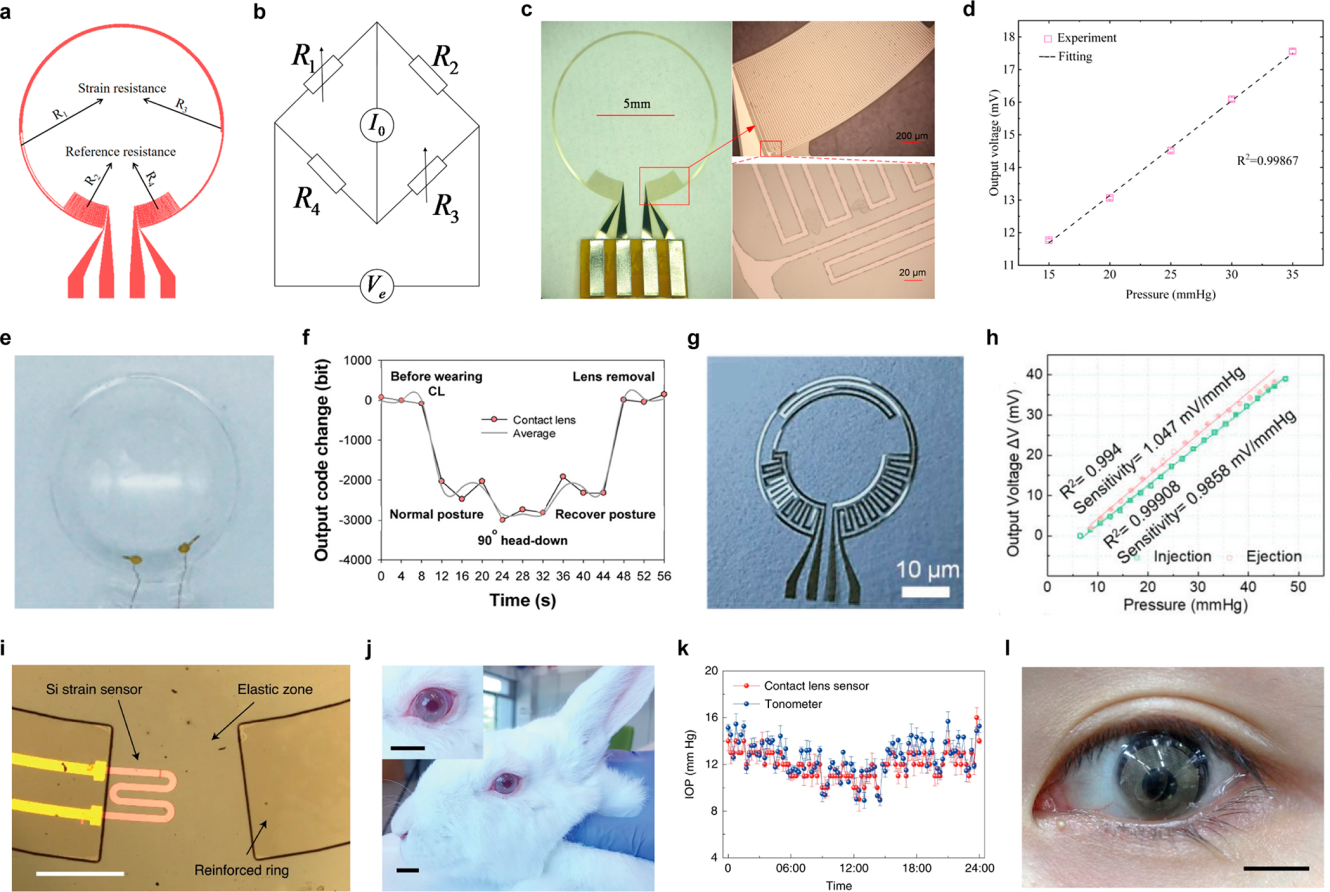
Piezoresistive sensor for IOP monitoring in SCL platform. (a) Schematic illustration of strain gauge sensor in IOP monitoring SCL platform. (b) Wheatstone bridge circuit diagram. (c) Photograph of 2D strain gauge sensor for IOP monitoring SCL platform (left) and zoom-in images (right). (d) Output voltage response to variations in pressure of eyeball model. (a–d) Reproduced with permission from ref (354). Copyright 2021 MDPI under CC BY 4.0 (https://creativecommons.org/licenses/by/4.0/). (e) Photograph of IOP monitoring SCL with AgNW-based piezo-resistive sensor. (f) Output code change with different postures of rabbit. (e, f) Reproduced with permission from ref (260). Copyright 2021 American Chemical Society. (g) Photograph of self-assembly graphene-based stain gauge. (h) Output voltage change along with the increase and decrease of pressure. (g, h) Reproduced with permission from ref (356). Copyright 2021 John Wiley and Sons. (i) Optical micrograph of IOP sensor on an elastic zone of the rigid-soft hybrid layer. Scale bar, 200 μm. (j) Photographs of a rabbit wearing IOP monitoring SCL. Inset: Magnified photograph of rabbit’s eye wearing IOP monitoring SCL. Scale bars, 1 cm. (k) 24-h monitoring of IOP from rabbit using an SCL and tonometer. (l) Photograph of human eye with IOP monitoring SCL. Scale bar, 1 cm. (i–l) Reproduced with permission from ref (32). Copyright 2021 Springer Nature.

As the strain is applied to an SCL when the IOP increases, the application of a strain sensor to an SCL facilitates monitoring of IOP through the lens platform. A strain gauge is a conventional method to detect the strain, using a Wheatstone bridge circuit (Figures 15a and 15b). Wheatstone bridge circuits detect the minute changes in strain by conversion into the change in resistance. This method is advantageous for the compensation of external disturbances, such as temperature, leading to accurate measurement of strain. As previous studies indicated, the Wheatstone bridge circuits-based strain gauge measures the strain applied on an SCL due to the increased radius of cornea curvature through detecting changes in output voltages or relative resistance. Dou et al. fabricated a strain gauge with platinum and titanium on a 2D substrate as an IOP sensor (Figure 15c).^354^ The strain gauges are transferred to the PDMS-based lens platform and connected with a source meter for power and recording of changes in output voltages. The PDMS-based lens, including strain gauges, was attached to a eyeball simulation system to identify the feasibility of the strain gauge as an IOP sensor. As a result, the output voltages increase as the pressure of the eyeball model increases with the correlation regression coefficient of 0.99867 (Figure 15d). In addition, this IOP sensing lens showed a sensitivity of 289.5 μV mmHg^–1^ within the range of normal IOP fluctuation.

Another method to detect IOP is the use of piezo-resistive material because its resistivity changes depending on the mechanical strain. When one subject wears an SCL that includes piezo-resistive material and the IOP of the subject increases, the deformations on the lens create microcracks on piezo-resistive material resulting in a decrease in the resistivity of the material.^355^ Monitoring of changes in resistance or output voltages by this mechanism can be correlated to IOP. As a piezo-resistive material, AgNW is a promising candidate for an IOP sensor integrated in SCL due to its transparency and electrical conductivity (Figure 15e).^260^ The correlation between the relative resistance of the IOP sensor using AgNWs and the applied pressure was examined by using the PDMS eye model, which can be used to simulate the increase in IOP by injecting PBS. The sensitivity of the AgNW-based IOP sensor was 0.0294% per 1 mmHg, which is high enough for detecting abnormal IOP levels. For wireless communication, an ASIC chip integrated with an AgNW IOP sensor converted the output current into bits, indicating that IOP fluctuations are presented as changes in the output code. The *in vivo* test showed that the SCL with an IOP sensor using AgNWs monitored the continuous change in IOP from the rabbits while they took different postures (Figure 15f).

Applying piezo-resistive material on a strain gauge can elevate the sensitivity of the IOP sensor on the SCL platform. Piezo-resistive materials, such as graphene or a hybrid of reduced graphene oxide and carbon nanotubes, are patterned into a zigzag pattern of strain gauges as IOP sensors, leading to higher sensitivity.^356^ For instance, Liu et al. fabricated a strain gauge with self-assembly graphene, and this sensor exhibited a sensitivity of 1.0164 mV mmHg^–1^ and a detection resolution of 0.24 mmHg (Figures 15g and 15h).

Another strategy to improve the sensitivity of the IOP sensor is to locally concentrate the strain to the sensor.^32^ With structural design using different material, a reinforced ring and an elastic zone were fabricated in the rim region of the lens, and the IOP sensor using the strain gauge design was created in the elastic zone where the strain is accumulated locally (Figure 15i). Kim et al. identified that the relative change in resistance of the strain sensor increases as the areal ratio of the reinforced ring to the elastic zone increases. Thus, the authors optimized the ratio of the reinforced area to elastic area as 9/1, leading to improved sensitivity of the relative change in the resistance of 0.05% per mmHg. Along with the strain sensor on rigid-soft hybrid layer, an antenna using ultralong AgNF and AgNW and an NFC chip were electrically connected and integrated into the SCL platform. Wireless power and data transmission through the NFC chip facilitated monitoring the IOP with a smart phone, which is accomplished by formation of a stretchable interconnection with liquid metal to prevent the electrical disconnection. The in vivo test with rabbits demonstrated 24-h continuous recording of IOP wirelessly (Figures 15j and 15k). Furthermore, the human study showed reliable sensing performance of this SCL, which validated the possibility of using the SCL platform as a monitoring system of IOP (Figure 15l).

##### 5.2.1.2 Inductive Coupling

**Figure 16:**
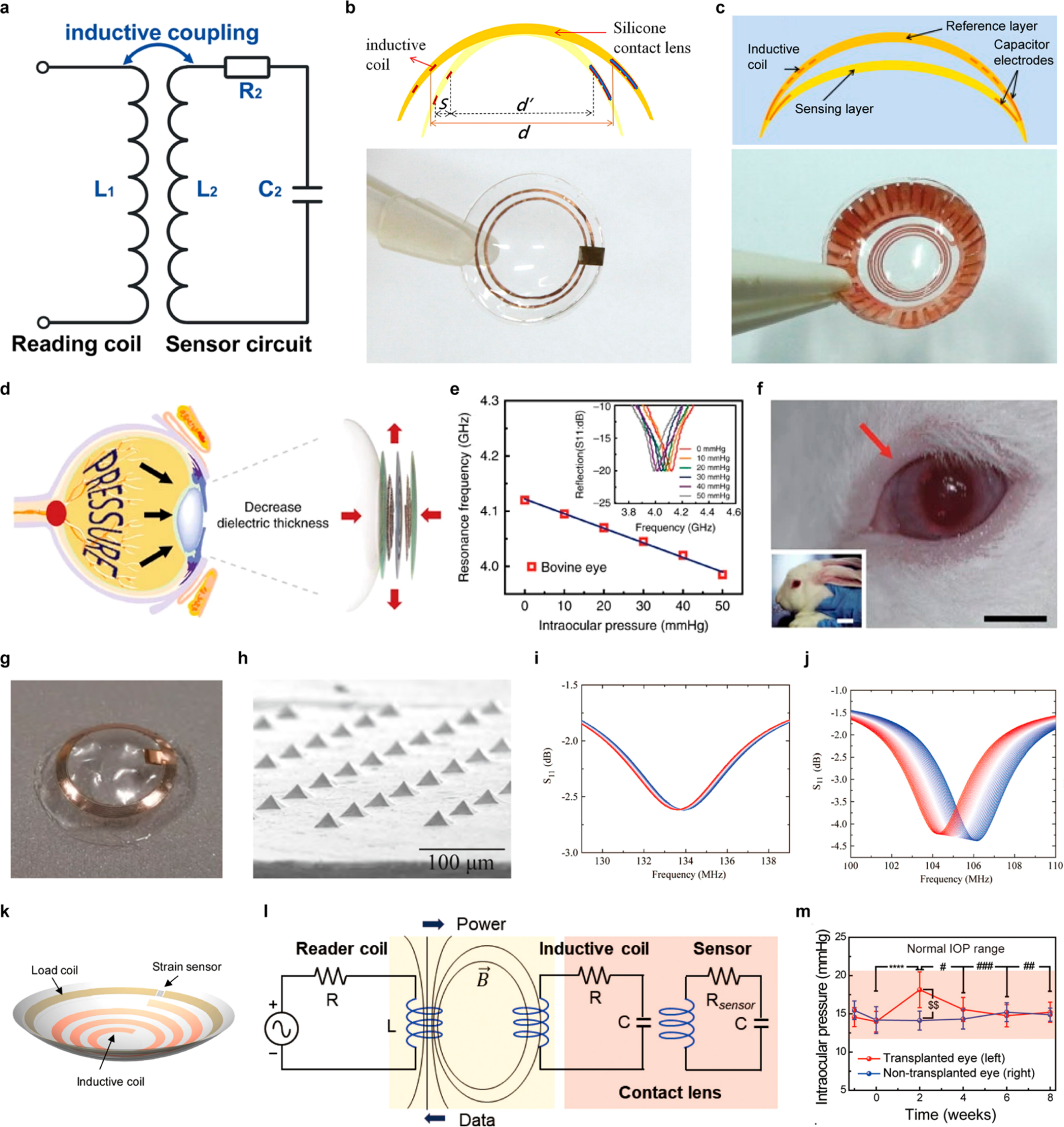
Inductive coupling sensor for IOP monitoring in SCL platform. (a) Circuit diagram of inductive coupling-based IOP sensor. Reproduced with permission from ref (342). Copyright 2021 John Wiley and Sons under CC BY 4.0 (https://creativecommons.org/licenses/by/4.0/). (b) Schematic illustration (top) and photograph (bottom) of inductance-based IOP sensor. Reproduced with permission from ref (358). Copyright 2014 Elsevier. (c) Schematic illustration (top) and photograph (bottom) of capacitance-based IOP sensor. Reproduced with permission from ref (360). Copyright 2013 Elsevier. (d) Schematic illustration of capacitance-inductance-based IOP sensor. (e) Photographs of rabbit’s eye wearing IOP monitoring SCL. Scale bars, 1 cm (black), 5 cm (white). (f) Resonance frequency response of IOP sensor on rabbit’s eye. (d–f) Reproduced with permission from ref (247). Copyright 2021 Springer Nature under CC BY 4.0 (https://creativecommons.org/licenses/by/4.0/). (g) Photograph of IOP monitoring SCL using a hydrogel-based dielectric layer. (h) SEM image of pyramid-microstructured Ecoflex. (i, j) S11 response to different pressures on IOP sensor with unstructured dielectric layer (i), and pyramid-microstructred dielectric layer (j). (g–j) Reproduced with permission from ref (345). Copyright 2022 American Chemical Society. (k) Schematic illustration of IOP monitoring SCL depending on resistance change. (l) Circuit diagram of the IOP sensor using shift in reflection value by resistance change at fixed resonance frequency. (m) Wireless monitoring on IOP of Lewis rats before and after islet transplantation. (k–m) Reproduced with permission from ref (362). Copyright 2020 American Chemical Society.

For wireless operation of SCL, resistor-inductor-capacitor (RLC) circuits have been adopted widely (Figure 16a).^357^ Detection of change in RLC circuits due to IOP fluctuation is one of the major methods used to detect IOP in the SCL platform. The resonance frequency, the main characteristic of the RLC circuit, can be expressed as the following equation:
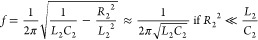
where *f* is the resonance frequency of the RLC circuit, and *L*_2_, *C*_2_, and *R*_2_ are the inductance, capacitance, and resistance of the sensor, respectively. As the equation implies, the resonance frequency can be shifted if either the inductance or the conductance changes. Thus, IOP can be monitored by detecting the degree of shift in resonance frequency due to the deformation of the SCL, which causes the alteration of inductance or capacitance.

First, the inductance of the RLC circuit can be changed by the shape deformation of the inductive coil. As the IOP induces change in the radius of the lens, the inductance of the RLC circuit integrated in an SCL can be changed, leading to shift in the resonance frequency of the corresponding circuit. For example, Chen et al. fabricated a RLC circuit with a spiral coil of copper, and they applied it to lens platform (Figure 16b).^358^ In order to improve the sensitivity of the inductance-based IOP sensor, An et al. used a stretchable inductive coil by replacing the rigid metal with liquid metal, i.e., Galinstan.^359^ The stretchability of coil offers advantages of higher sensitivity of 415–158 ppm mmHg^–1^ and more comfortable usability.

Another main factor that can affect the shift in resonance frequency is the capacitance of the RLC circuit. The capacitance depends on the thickness of the dielectric layer, which can be fluctuated by the deformation of the lens due to the changes in the IOP. Chen et al. fabricated a double layer of Cu coils with an air dielectric layer as a capacitive IOP sensor (Figure 16c).^360^ As the air dielectric layer is compressed by the increased IOP, the capacitance of the RLC circuit integrated in the lens increased, leading to a negative shift in the resonance frequency. This IOP sensor exhibits reliable sensitivity of 240 ppm mmHg^–1^ to detect the human IOP range. However, the formation of an air dielectric layer on the SCL platform is challenging because its lens has a small, curved structure. Thus, several researchers have presented various types of dielectric layers to replace the air dielectric layer and to enhance the sensitivity of the capacitive IOP sensors. Kim et al. used a silicone elastomer, Ecoflex, as a dielectric layer between two inductive spiral coils of AgNW.^320^ As the IOP increases, the distance between the coils decreases, and the two stretchable, spiral coils expand, which results in the increase of both conductance and inductance, resulting in a negative shift in resonance frequency (Figures 16d and 16e). This IOP sensor shows the sensitivity of 2.64 MHz mmHg^–1^, and it showed reliable operation on a bovine’s eye (Figure 16f). As an another example, Zhang et al. chose Silbione as a dielectric material and polystyrene-*b*-poly(ethylene-ran-butadiene)-*b*-polystyrene (SEBS) embedded with silver flakes (AgSEBS) as a material for inductive serpentine-shaped coils, which enables the elevation of both inductance and capacitance by increased IOP.^247^ Owing to low elastic modulus of Silbione compared to other silicone elastomers, the utilization of Silbione as a dielectric layer enhances the conductive sensitivity of the IOP sensor to 6.8 × 10^–4^ mmHg^–1^. The authors also demonstrated the continuous operation of the IOP monitoring SCL with dogs for 24 h reflecting the daily IOP fluctuation of the dogs.

In addition to adopting another material for the dielectric layer, structural modification of the dielectric layer also can enhance the capacitive sensitivity of the IOP sensor. Pyramid-microstructure hydrogel-based dielectric layer offers higher sensitivity as it can be deformed more under the same pressure compared to the unstructured dielectric layer (Figures 16g and 16h). The formation of the 12 × 12 array of the pyramid-microstructure with Ecoflex lowered the elastic modulus compared to pristine Ecoflex, leading to the high sensitivity of 1.101‰ mmHg^–1^ (Figures 16i and 16j).^361^

Along with the inductance and capacitance, the resistance of the RLC circuit can alter its characteristics. Kim et al. integrated the RLC circuit, including the resistor of the strain sensor on the load coil, inductor, and capacitor antenna coils into the SCL platform (Figure 16k). Figure 16l describes the circuit diagram of the IOP sensing SCL.^362^ This lens utilized the change in the reflection value (S11) at a fixed resonance frequency to measure the IOP of a subject. As the lens deformed according to the elevation of IOP, the resistance of the strain sensor on the load coil increases, resulting in an increment in S11, as shown in the following equation:
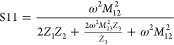
where ω is the angular frequency of the sensor, *M* is the coupling coefficient, and *Z* is the resistance of the reader coil (1), the inductive coil (2), and the load coil (3), respectively. Adoption of this mechanism of the IOP sensor to the SCL for rats could monitor the IOP of the streptozotocin-induced diabetic Lewis rats before and after the treatment of islet implantation (Figure 16m). This result indicates that the IOP monitoring SCL is a reliable platform for monitoring the condition of patients during the treatment.

##### 5.2.1.3 Microfluidic Channel

The integration of the microfluidic system with the SCL provides a biocompatible and noninvasive method for IOP monitoring without any electronic component. The microfluidic SCL detects IOP by converting the corneal deformation into fluidic volume changes.

**Figure 17:**
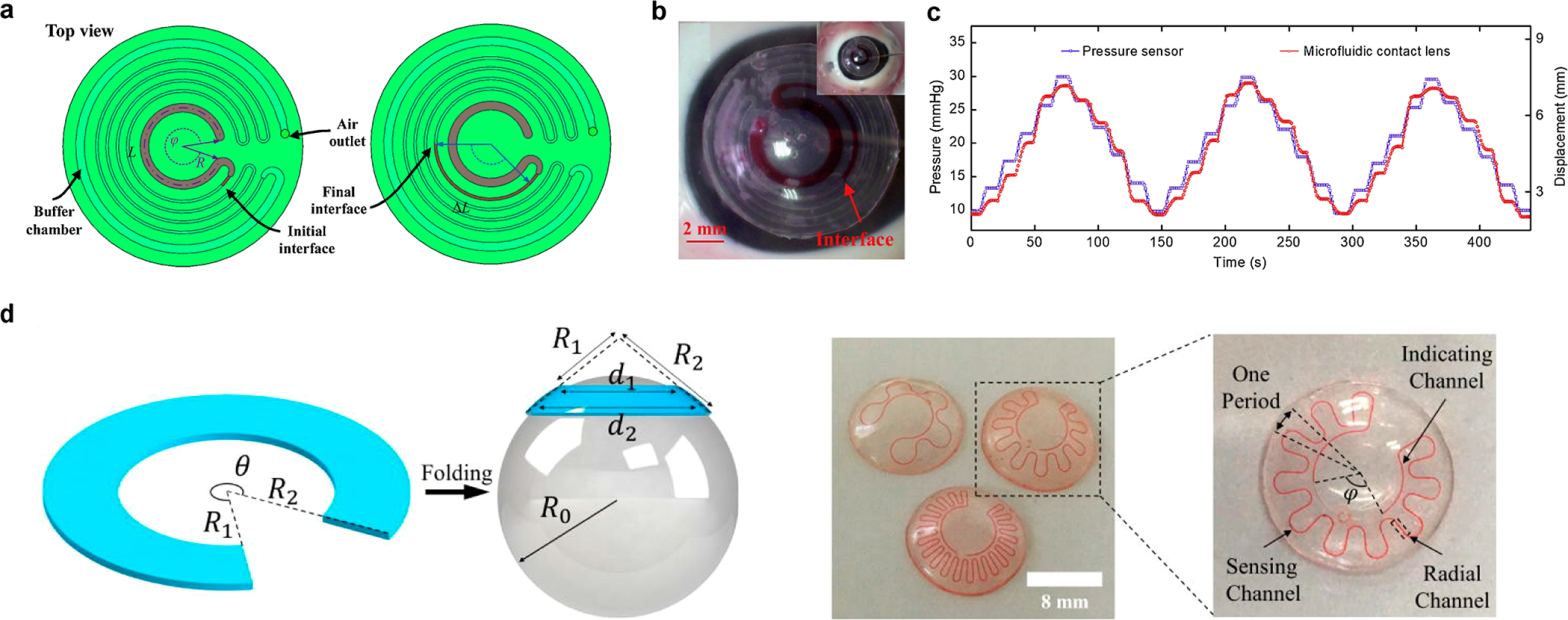
Physical diagnostic SCLs for IOP monitoring from microfluidic channel. (a) Schematics of microfluidic contact lenses under different IOP. (b) Photograph of the microfluidic contact lens onto the porcine eye *ex vivo*. (a, b) Reproduced with permission from ref (363). Copyright 2019 Elsevier B.V. (c) Variation of displacement and IOP during three cycles. (d) Microfluidic SCL with a notched-ring structure for distortion-free fabrication. (c, d) Reproduced with permission from ref (364). Copyright 2021 Yang et al.

An et al. introduced the microfluidic SCL for continuous IOP monitoring, with visual monitoring of the flow of the dyed liquid.^363^ As the IOP increases, the dyed liquid, which originally was located in the sensing chamber, flows toward the sensing channel. When the IOP returns to normal levels, the dyed liquid flows back to the sensing chamber (Figure 17a). To quantify the displacement of the liquid flow, the authors measured it while applying IOP within the range of 0 to 40 mmHg and calculated the sensitivity of the microfluidic IOP sensor. The adaptability and utility of the microfluidic IOP sensor incorporated into an SCL also were demonstrated through an *ex vivo* test on a porcine eye. The IOP level of the porcine eye was monitored in real-time using an SCL (Figures 17b and 17c). The displacement of the dyed liquid in the microfluidic sensor followed an identical pattern (blue line) with the IOP applied on the porcine eye (red line). In addition, the sensitivity of the sensor was measured to be 0.283 mmHg^–1^.

Incorporating a microfluidic sensor into an SCL poses a challenge due to the distortion of the structure that occurs when transforming a planar microchannel into a 3D structure to accommodate the corneal curvature. It also is crucial for continuous monitoring of the IOP and ocular conditions. To address this issue, Yang et al. designed a microfluidic SCL with a notched-ring structure to achieve the distortion-free 2D-to-3D transformation during fabrication.^364^ In addition, they optimized the geometry of the microchannel in the sensor to form a serpentine structure, which minimizes distortion during the folding process (Figure 17d). The performance of this IOP SCL was tested on an artificial silicon model eye that mimics the human cornea, specifically in terms of thickness and diameter.

#### 5.2.2 Temperature

The ocular surface temperature (OST) varies with diverse circumstances, such as eye blinking, aging, and temporal variation of body temperature. An abnormal ocular condition also can be a factor that affects the OST. For instance, eye inflammation increases the blood flow of the anterior eye, which in turn leads to an increase in the OST.^365^ In addition, measuring the OST is used to diagnose dry eye disease and diabetic retinopathy.^366^

**Figure 18:**
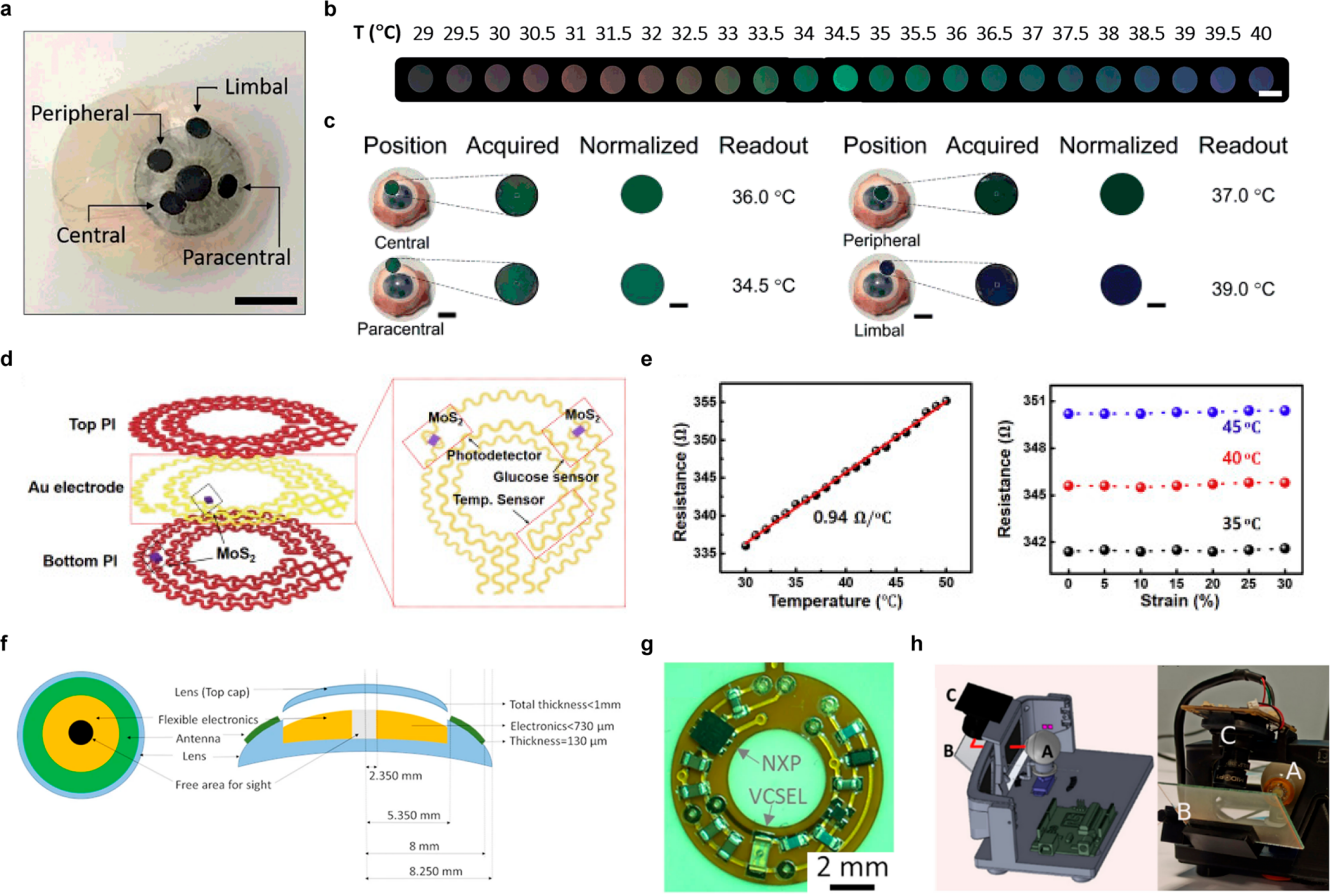
Physical diagnostic SCLs for monitoring of ocular surface temperature and eye movement. (a) Photograph of an SCL with four CLC temperature sensors. Scale bar, 1 cm. (b) Screenshot of normalized color images of CLC in different temperatures. Scale bar, 2 mm. (c) Smartphone readouts of the color of CLC at four sensing areas. Scale bars, 2 mm. (a–c) Reproduced with permission from ref (367). Copyright 2019 Te Royal Society of Chemistry under CC BY-NC 4.0 (https://creativecommons.org/licenses/by-nc/4.0/). (d) Schematics of a gold wire temperature sensor in multifunctional SCL. (e) The temperature-dependent resistance curve for gold wire temperature sensor (left). The resistance of the temperature sensor according to strain under different temperatures (right). (d, e) Reproduced with permission from ref (338). Copyright 2020 Elsevier Inc. (f) Schematics of the composition of eye tracking SCL. (g) Electronic circuit and components of eye tracking lens. Scale bar, 2 mm. (h) Schematic and photograph of the overall system depicting the artificial eye. (f–h) Reproduced with permission from ref (370). Copyright 2022 Maowen Xie et al. under CC BY 4.0 (https://creativecommons.org/licenses/by/4.0/).

The SCL can directly and effectively measure the OST. Moreddu et al. developed an SCL with an embedded temperature-sensitive, cholesteric liquid crystal (CLC).^367^ This SCL has four CLC sensors located at different sensing areas on the cornea to detect the changes in OST of each position (Figure 18a). The molecular arrangement of CLC forms a helical structure that can change in response to the temperature. Since CLC reflects specific wavelengths of light depending on the length or shape of the helical structure, it has temperature-sensitive reflection spectra that changes its color. The authors acquired a color image of CLC embedded in SCL using a smart phone, which automatically normalized the image. Figure 18b shows the list of normalized color images of CLC obtained by applying the temperatures ranging from 29 to 40 °C with a step size of 0.5 °C. The colorimetric analysis using the SCL was tested on an *ex vivo* porcine eye heated on a hot plate, as shown in (Figure 18c).

Guo et al. reported a multifunctional SCL that integrates a photodetector, glucose sensor, and temperature sensor.^338^ The photodetector and glucose sensor were based on an MoS_2_ transistor and consisted of individual drain electrodes and one common source electrode, allowing them to independently receive optical information and monitor the glucose levels. The temperature sensor was separately integrated through a thin, serpentine gold wire electrode (Figure 18d). This temperature sensor was designed to detect OST by measuring changes in the resistance of the gold wire. The sensitivity of the sensor was calculated as 0.94 Ω °C^–1^ within a temperature range of 30 to 50 °C. In addition, the authors demonstrated that the mechanical deformation of the gold wire sensor can be ignored by observing the resistance when applying mechanical strain (Figure 18e).

#### 5.2.3 Eye Movement

Eye movement reflects human consciousness, which is a result of a complex neural process that involves multiple levels of control. This process begins with the cerebral cortices and continues through the midbrain structures, ultimately reaching the lower brainstem where the desired eye movement is integrated. Therefore, eye tracking can be used to evaluate cognitive or attentional disorders.^368^ Furthermore, it has been studied to clarify the connection between the eye movement and neurodegenerative diseases, such as AD.^369^

Khaldi et al. measured eye movement using an SCL.^370,371^ They introduced the prototype eye tracking lens composed of the electronics, antenna, and encapsulations (Figure 18f). Figure 18g shows a circuit of electronics where the infrared (IR) beam is emitted to directly indicate the direction of eye gaze. It is combined with a secondary antenna embedded in the eye tracking lens to receive power from an eyewear power supply system. This system contains the primary antenna that transfers power from an external generator to the secondary antenna of the lens by magnetic coupling. For the eye tracking test, a camera was mounted on the eyewear, and the IR beam from the eye tracking lens put on an artificial eye was reflected by a beam splitter toward the camera (Figure 18h). The camera successfully recorded five different gaze directions.

However, this SCL had some limitations for use on human eyes. The excessive number of components in the electronics resulted in an inadequate weight for perfectly fitting the eye. As a result, the lens descended slightly and was below the center of the pupil, which could potentially affect vision when worn by a person. Furthermore, it is necessary to verify whether the radiofrequency for wireless communication or the IR laser exposed to the eye can lead to any severe risks to the human body. Therefore, further research is needed to enable the safe and suitable application of this eye tracking system to the SCL for human use.

### 5.3 Electrophysiological Diagnosis

The ERG is a diagnostic test that quantifies the electrophysiological activity of various neuronal and non-neuronal cells in the retina under light stimulation.^16,286−289^ In other words, the ERG measures the electrical potential differences associated with the retina’s response to a light stimulus. It is commonly used in ophthalmology as a standard clinical procedure for the diagnosis of many ocular diseases, such as glaucoma, diabetic retinopathy, RP, and other congenital degenerations.^372−377^

**Figure 19:**
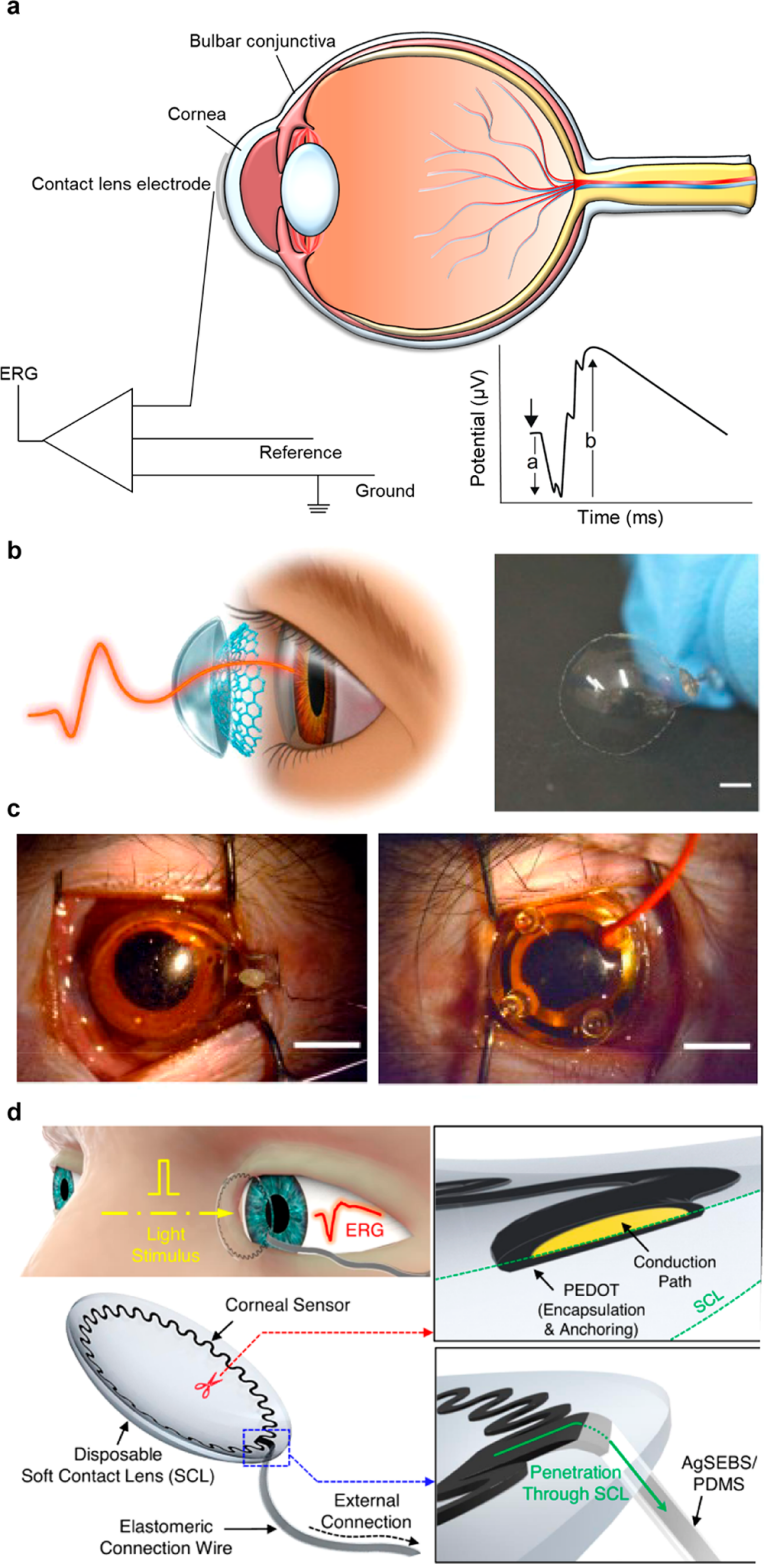
SCLs for electrophysiological recording. (a) ERG signals with a biphasic waveform characterized by the initial negative a-wave followed by the subsequent positive b-wave. (b) An image of soft, transparent graphene-based contact lens electrodes (GRACEs). (c) Photographs of a GRACE device and commercial jet electrode attached to the cornea of monkey. (b, c) Reproduced with permission from ref (377). Copyright 2018 Spring Nature under CC BY 4.0 (https://creativecommons.org/licenses/by/4.0/). (d) Schematic illustrations of all-printed stretchable corneal sensors on a commercial disposable soft contact lens. Reproduced with permission from ref (383). Copyright 2021 Spring Nature under CC BY 4.0 (https://creativecommons.org/licenses/by/4.0/).

As shown in Figure 19a, the detected ERG signals showed a biphasic waveform characterized by the initial negative a-wave followed by the subsequent positive b-wave. The a-wave is reflected by the activity of the cones and rods in the outermost layer of photoreceptors, and the b-wave is reflected by the activity of the amacrine, horizontal, bipolar, and muller cells in the inner retina. Specifically, the amplitude of the a-wave is the voltage difference between the baseline (0 μV) and the a-wave’s peak; the amplitude of the b-wave is the voltage difference between the a-wave’s peak and the b-wave’s peak; the implicit time of the a-wave and b-wave refers to the time difference between the first flash and each wave’s peak, respectively. To differentiate between normal and abnormal retina conditions, the characteristics of the amplitude and implicit time of these waves are used as valuable diagnostic tools. For example, patients with rod-cone dystrophy as the most common form of RP show a decrease in the amplitude and implicit time of the b-wave due to rod photoreceptor death. The degree of the severity of RP results in more death of these cells, further reducing the amplitude of both the a-wave and the b-wave.^378,379^

Generally, the detection of ERG signals is performed by three electrodes, (i.e., a recording electrode, a reference electrode, and a ground electrode).^33,372^ A recording electrode is positioned in direct contact with either the corneal surface or bulbar conjunctiva, along with the placement of a reference electrode and a ground electrode on the forehead and earlobe, respectively. The most common types of corneal electrodes (e.g., the Burian-Allen lens and the ERG-Jet lens) are comprised of a contact lens integrated with a metal conductor at the edge.^372,382,383^ These lens-based devices with a blepharostat offer strict corneal contact, thereby providing ERG signals with higher amplitude, higher sensitivity, and lower noise than conjunctival electrodes. However, they result in discomfort that arises from the use of a thick, rigid, hard contact lens that is not compatible to the soft and sensitive ocular structures with anteriorly protruding bumps and large outer curvature. To address this discomfort, hook and wire-type conjunctival electrodes (e.g., the Dawson Trick Litzkow (DTL) fiber) are introduced as alternatives.^380−382^ These electrodes have much-reduced discomfort as they do not disturb the central vision by hooking up the lower eyelid and placing them in the conjunctival fornix, and they also provide high stability and reproducibility of the signals. However, the use of these devices is associated with a significant reduction in signal quality, with amplitudes being reduced to those obtained using corneal electrodes due to the substantial distance from the cornea, thereby limiting the precise interpretation of the ERG signals. Recently, technological advances in transparent electrode materials, biosensors, and wireless modules have developed SCL that combined soft corneal electrodes and soft contact lenses for ERG detection.^377,383,384^ These SCL offer many advantages. First, the electrodes with softness enhance the level of comfort by achieving conformal contact with the sensitive ocular structures during ERG recordings. Second, the conformal contact between the soft electrodes and the curved surface of the cornea provides stable electrical and mechanical contact without any fixture (e.g., a blepharostat), resulting in high reliability and accuracy of the ERG signals that are acquired. Third, this conformal interfacing between the SCL and eyes serves to prevent the occurrence of thick tear films or air gaps, which is a critical factor in preserving the eye’s refraction. Fourth, the optical transparency of corneal electrodes allows the ERG response across the cornea compared to the conventional design with opaque electrodes, which only capture ERG near the edge of the cornea. This is crucial for correlating the distribution of corneal potentials in response to retinal activity and detecting localized retinal dysfunction under the stimulus. As a result, these potential advantages have enabled the development of various SCLs for ERG sensing.

With the advantage of the high electrical conductivity and optical transparency of graphene material, Yin et al. developed soft, transparent graphene-based contact lens electrodes (GRACEs) and conducted ERG sensing from cynomolgus monkeys and rabbits, as shown in Figure 19b.^377^ The GRACE devices offered superior conformal contact and tighter interface with the cornea (Figure 19c, left) compared to the conventional ERG jet electrodes (Figure 19c, right). As a result, they exhibit enhanced mechanical stability and high signal amplitude, enabling the capacity for high-quality, multifocal ERGs (mfERGs). Moreover, in the *in vivo* test, the graphene-based multielectrode arrays detected full corneal ERG signals owing to the optical transparent property, which is essential for ocular electrophysiology studies.

For practical ERG sensing, Kim et al. reported all-printed stretchable corneal sensors on a commercial, disposable, soft contact lens, as shown in Figure 19d.^383^ With this approach, the fabricated device offered many advantages compared to the conventional ERG jet electrodes. The device consisted of a commercial soft contact lens, a printed corneal sensor, an anchoring polymer, and an external elastomeric connection wire. By using the commercial soft contact lens with its super softness, biocompatibility, transparency, and wettability, they achieved conformal contact without the use of a speculum and corneal anesthesia, resulting in high-fidelity ERG signals. The direct printing methods of a corneal sensor on the commercial soft contact lens do not alter the good properties (i.e., softness, biocompatibility, transparency, and wettability), and they make a conductive pathway to external measurement as a single device. Moreover, the electrochemical anchoring of poly(3,4-ethylenedioxythiophene) further enhanced the mechanical and electrical stability of the fabricated devices, even under harsh conditions. As a result, the fabricated ERG SCL was >25-fold thinner, >3-fold lighter, and >2000-fold softer than the conventional ERG jet lens.

### 5.4 Optical Diagnosis

Extensive research has been conducted on contact lens sensors that enable the monitoring and transmission of information from the tear fluid of diverse analytes.^385,386^ In particular, SCLs that rely on colorimetric, fluorescence, and photonic crystal sensing mechanisms leverage their optical properties.

Recently, the integrated form of SCLs has witnessed a surge in the application of colorimetric sensing, primarily due to its remarkable specificity, sensitivity, and feasibility. Colorimetric sensing involves the utilization of a specific color reagent under a particular wavelength of light to quantify the concentration of a target organic or inorganic compound in a solution (e.g, tear fluid), with the advantage of flexible use of enzymatic chemicals.^387−391^
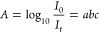


The Beer–Lambert Law relates the optical attenuation of physical material, when incident light passes through a solution, it undergoes reflection, absorption, and transmission, and can be expressed as a product of the optical path length and concentration of the attenuating species, where *A* is the absorbance, *I*_0_ is the intensity of incident light, *I*_*t*_ is the intensity of transmitted light, *a* is the absorptivity, *b* is the optical path length, and *c* is the concentration of the attenuating species.^392^ Therefore, any changes in the concentration of the tear fluid can be ascertained by measuring the absorbance of the incident light, and such changes typically are accompanied by observable color changes in the colorimetric sensor integrated with a contact lens.

**Figure 20:**
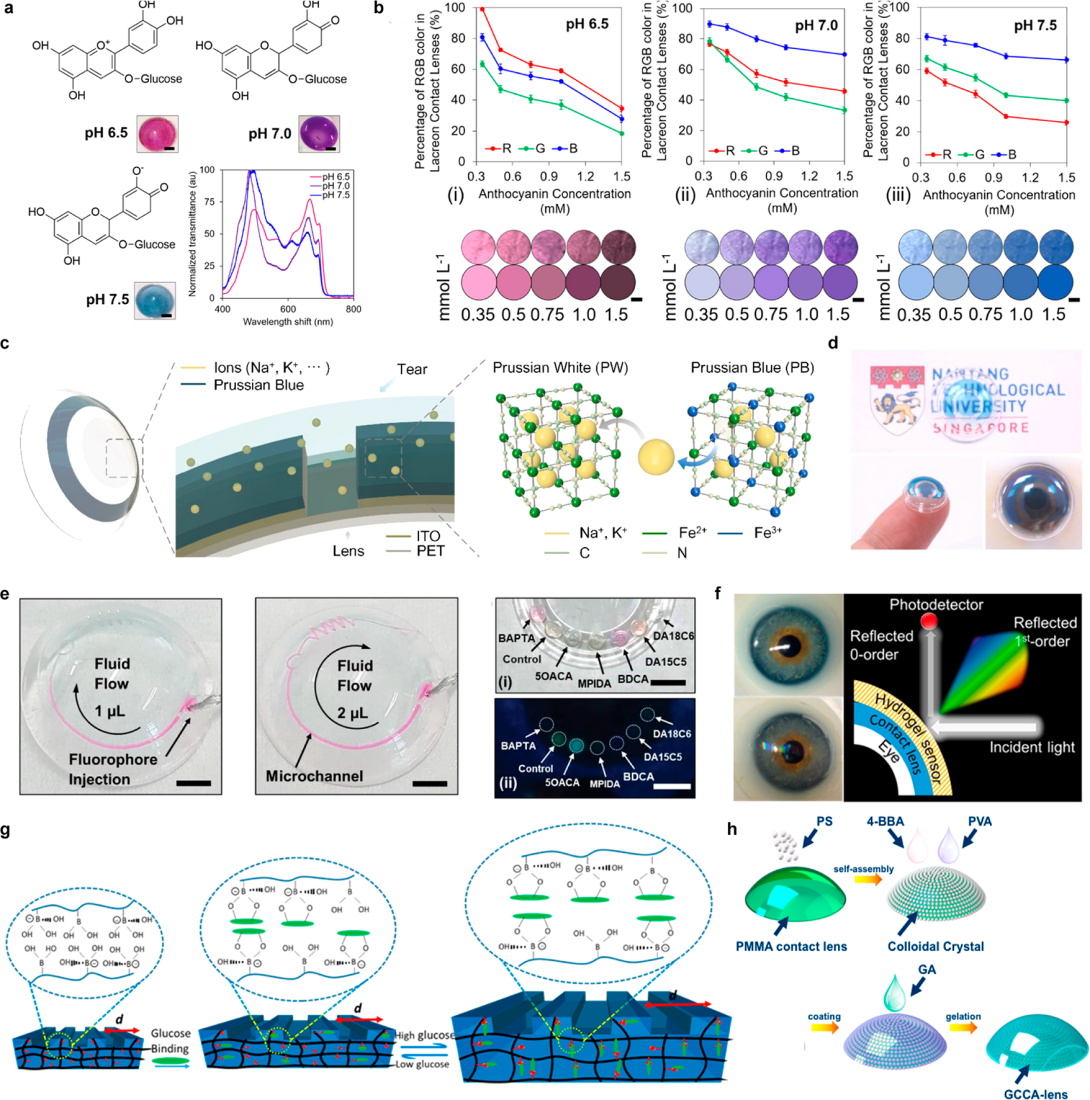
SCLs with optical properties. (a) Colorimetric SCLs that were functionalized with anthocyanin pigments for monitoring the pH level in tear fluid. (b) Colorimetric changes indued by pH differences of colorimetric SCLs. (a, b) Reproduced with permission from ref (387). Copyright 2019 American Chemical Society under CC BY-NC 4.0 (https://creativecommons.org/licenses/by-nc/4.0/). (c) The ratio changes of iron oxidation states and input of sodium and potassium ions by the electrochemical reactions. (d) The color changes induced by applying the voltage. (c, d) Reproduced with permission from ref (388). Copyright 2020 Elsevier B.V. (e) Fluorescence-based SCLs with 6-multiplexed using concavities by measuring electrolyte concentrations in tear fluid. Reproduced with permission from ref (389). Copyright 2019 Wiley under CC BY 4.0 (https://creativecommons.org/licenses/by/4.0/). (f) Photographs of both commercial contact lens and photonic-based contact lens, and the schematic illustrations of color changing process by volume change. (g) The schematic illustrations of the periodic photonic structures printed with a 1.6 μm distance on a hydrogel film with phenylboronic acid which modulates the volume change in response to glucose binding. (f, g) Reproduced with permission from ref (390). Copyright 2018 American Chemical Society under CC BY 4.0 (https://creativecommons.org/licenses/by/4.0/). (h) An image of the device consisted of a three-dimensional polystyrene CCA embedded in hydrogel functionalized with 4-boronobenzaldehyde (4-BBA)-modified poly(vinyl alcohol) (PVA) and gas permeable poly(methyl methacrylate) (PMMA)-based contact lens. Reproduced with permission from ref (391). Copyright 2017 MDPI under CC BY 4.0 (https://creativecommons.org/licenses/by/4.0/).

For monitoring the pH level in tear fluid based on colorimetric sensing, Riaz et al. developed SCLs that were functionalized with anthocyanin pigments extracted from *Brassica oleracea*, and they evaluated their color response to different ocular pH levels of 6.5, 7.0, and 7.5 as shown in Figure 20a.^387^ The SCLs exhibited a color shift from pink (pH 6.5) to blue (pH 7.5) with increasing pH values, and the functionalization was optimized based on dye concentration and soaking time. As shown in Figure 20b, to get the best results, the RGB color model was used to compare the results obtained from the naturally extracted dye and delphinidin chloride dye in an aqueous solution ranging from 0.0 to 1.5 mM to quantify the color changes of the anthocyanin functionalized SCL. Also, the soaking time of 24 h showed the best results. The leakage of the dye from the contact lenses was minimal for 18 h, and the developed sensors showed potential for continuous monitoring of ocular pH at point-of-care settings.

By using the visualization of images through colorimetric properties, Kim et al. presented SCLs based on electrochemical reactions that enable the direct transfer of data through color changes.^388^ With this system, they can prevent emergencies by visualization encoded images to warn deaf people. They consisted of two PB-based electrodes above and surrounding the pupil (i.e., one is for the WE, and the other is for the CE). In normal situations, the electrode above the pupil sustains transparent color with clear sight; however, the electrode above the pupil undergoes color change. The color changes were induced by applying the voltage; the electrochemical reactions occur with the ratio changes of iron oxidation states and input of sodium and potassium ions, as shown in Figures 20c and 20d. Furthermore, various pattern visualization encoded by controlling the duration of voltage according to the emergency situations of prerecorded video.

In the fluorescence basis, fluorophores, which are synthetic chemical compounds, are excited from the ground state to a singlet state by absorption of a specific wavelength of light and relaxed to a lower energy state by emitting the light with a longer wavelength.^393,394^ The wavelength of light during excitation and relaxation is determined by the chemical structure and composition of the target fluorophore, and the rapid transition of this fluorophore in microsecond enables high molecule-specificity. Therefore, pH and the major electrolytes present in tears Na^+^, K^+^, Ca^2+^, Mg^2+^, Cl^–^, and Zn^2+^, can be detected selectively for real-time fluorescence monitoring related to various ocular diseases, such as dry eye disease and diabetic retinopathy.^373,393^ Moreover, these interactions between fluorophores and target analytes are sensitive and cause little tissue damage. With these advantages, fluorescence-based SCLs have been developed as optical sensors.^389,393^

Yetisen et al. presented fluorescence-based SCLs to quantitatively measure the concentrations of electrolytes in tear fluid for point-of-care diagnosis of ocular diseases.^389^ They were based on fluorescent probes to measure pH and electrolyte concentrations, which were 6-multiplexed using concavities fabricated by laser ablation, as shown in Figure 20e. Each 6-multiplexed concavity and the microchannels were encapsulated with silicone hydrogel to prevent the leakage of tear fluid and to enable the spread of the electrolytes. To determine the pH and the electrolytes (Na^+^, K^+^, Ca^2+^, Mg^2+^, and Zn^2+^) values for optical measurements, benzenedicarboxylic acid (BDCA)-based fluorescent probes were used for pH, crown ether derivatives-based probes were used for Na^+^ and K^+^ ions, a 1,2 bis(o-aminophenoxy)ethane-*N*,*N*,-*N*′,*N*′-tetraacetic-acid–based probes were used for Ca^2+^, 5-oxazolecarboxylic acid-based probes were used for Mg^2+^, and *N*-(2-methoxyphenyl)iminodiacetate-based probes were used for Zn^2+^. Also, a portable readout platform with a light-emitting diode, optical filters, and a smartphone camera was used to obtain quantitative measurements. This overall hand-held system allows the delivery of *in vivo* diagnostics of ocular conditions to patients whenever they want.

Some SCLs are integrated with photonic-based sensors. These sensors are based on photonic crystals, which are periodically structured dielectric materials that exhibit distinct photonic bandgap and diffraction properties upon interacting with specific target analytes.^395,396^ With these periodic properties of photonic crystals, photonic-based sensors are classified into one-dimensional (i.e., periodic dielectrics occur in one direction), two-dimensional (i.e., periodic dielectrics occur in two directions), and three-dimensional (i.e., periodic dielectrics occur in three directions) photonic crystals.^373,395^
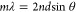


Based on Bragg’s law, when a beam of light with a particular diffraction order (*m*) is directed at a crystal lattice at an angle relative to the plane of refraction (θ), any variations in the interparticle distance (*d*) or refractive index (*n*) within the lattice will cause a shift in the wavelength of the diffracted light. Therefore, SCLs utilized with photonic-based sensors have been studied to measure the target analyte concentrations in tears for various ocular diseases.^390,391^

Elsherif et al. presented a wearable SCL integrated with photonic-based sensors that can measure glucose levels continuously, with readouts accessible through a smartphone.^390^ The periodic photonic structures were printed with a 1.6 um distance on a hydrogel film with phenylboronic acid, which modulates the change in the volume in response to glucose binding. As a result, the volume change in the sensors caused a difference in the diffraction angle and in the interspacing between the zero- and first-order interface, as shown in Figures 20f and 20g. SCLs integrated with photonic-based sensors showed a short response time of 3 s and a saturation time of 4 min.

With the same mechanisms above, Ruan et al. designed a crystalline colloidal array (CCA)-based photonic sensor in the form of SCLs for monitoring the levels of glucose in tears.^391^ The device consisted of a three-dimensional polystyrene CCA embedded in hydrogel functionalized with 4-boronobenzaldehyde (4-BBA)-modified PVA and gas permeable poly(methyl methacrylate) (PMMA)-based contact lens, as shown in Figure 20h. As a result, the diffracted light due to the volume changes underwent a change in wavelength between 567 and 468 nm in response to the glucose concentration ranging from 0 to 50 mM.

Diagnostic SCLs have received considerable attention in recent years due to their ability to noninvasively analyze tear components and provide valuable insights into a person’s health status. Although several challenges remain, tears contain biomarkers for various metabolic diseases and neurological disorders, highlighting the many benefits and potential of diagnostic SCLs. By analyzing biomarkers present in tears, such as proteins, enzymes, metabolites, and genetic materials, SCLs can potentially detect and monitor early signs of various diseases. The real-time monitoring capabilities of diagnostic SCLs have the potential to revolutionize the healthcare field by enabling early detection and intervention. It could offer a convenient and continuous monitoring solution, reducing the need for invasive or sporadic testing methods. Moreover, SCLs can provide personalized health information and enable healthcare providers to deliver timely interventions and treatments based on individual needs.

However, further advancements are still required to fully exploit the potential of diagnostic SCLs. For example, improvements in manufacturing techniques are needed to ensure comfortable and reliable long-term wear. Further advances in sensor technology, data analysis algorithms, and wireless communication are also necessary for real-time monitoring and seamless integration with biomedical systems.

In conclusion, diagnostic SCLs hold great promise as a noninvasive and continuous monitoring method. Although some challenges remain in terms of mass production technology and comprehensive understanding tear physiology, continued research and technological advances are expected to unlock the full potential of diagnostic SCLs to revolutionize disease detection, management, and personalized healthcare.

## 6 Therapeutic SCLs

Therapeutic SCLs are an emerging technology that has the potential to revolutionize healthcare by enabling real-time monitoring and treatment of various ocular and systemic conditions. These lenses are equipped with DDSs, miniaturized electronics, and heaters that can detect changes in physiological parameters, such as IOP, tear composition, and glucose levels, and provide targeted therapy. The ability of these lenses to continuously monitor and manage various health conditions can improve patients’ outcomes, increase treatment adherence, and reduce healthcare costs. In this context, this paper provides an overview of the development, design, and various applications of therapeutic SCLs in the field of ophthalmology and beyond.

### 6.1 Drug Delivery

**Figure 21:**
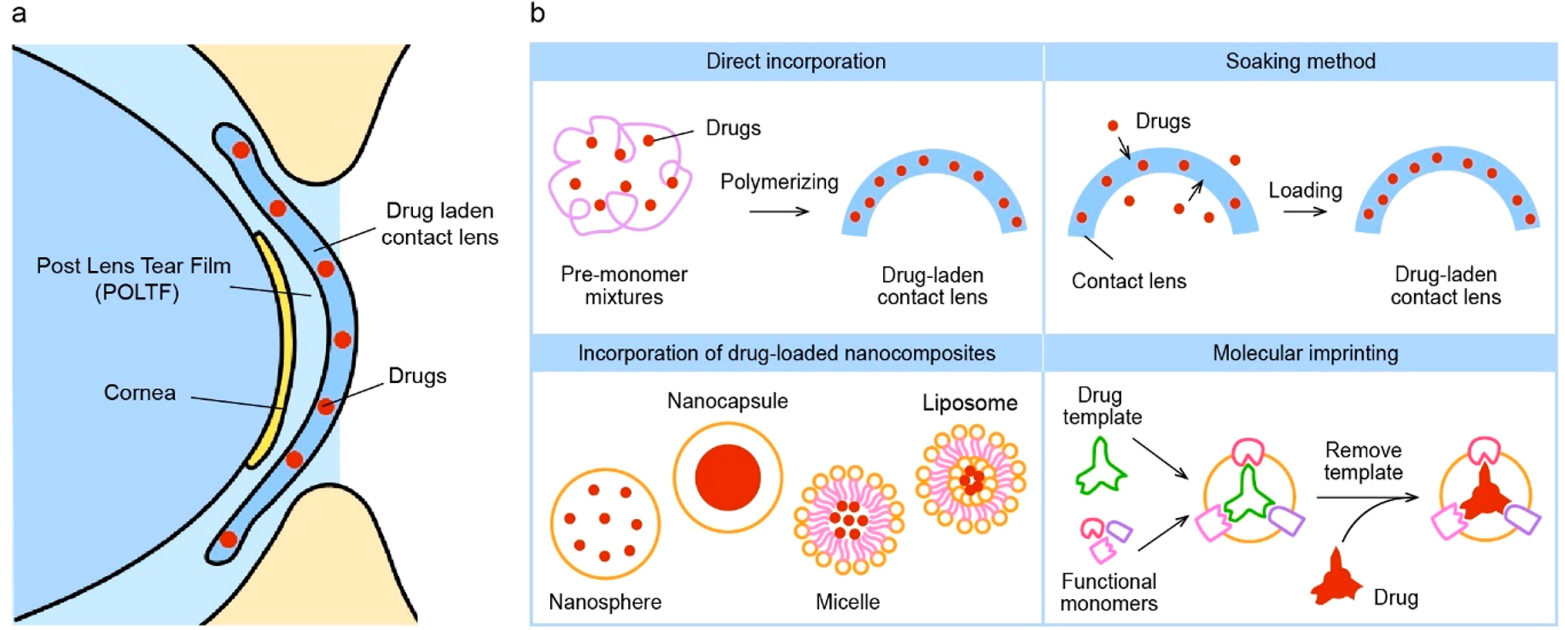
Methods of drug delivery using SCLs. (a) Schematic of the drug-laden contact lens inserted in the eye. (b) General methods for loading drugs into contact lenses.

Drug delivery through contact lenses has emerged as an innovative approach to treating ocular diseases since Sedlavek in Czechoslovakia first employed soft contact lenses in 1965. This approach has the potential to revolutionize traditional DDSs by incorporating drugs into the contact lenses themselves, allowing for sustained release directly to the eye. The main idea was to disperse drugs in the contact lens matrix, as shown in Figure 21a. When drug-loaded contact lenses are worn, the drug will diffuse from the contact lens and enter the postlens tear film (PLTF). This approach offers advantages such as increased convenience, improved patient compliance, and reduced risk of side effects compared to eye drops or injections. Recent progress in developing drug-eluting contact lenses has led to several products already on the market or in development, which can potentially transform the treatment of eye diseases, including glaucoma, dry eye, and corneal infections. We will discuss the different types of DDSs in contact lenses, which have evolved from mere vision correction devices to multifunctional tools that provide therapeutic benefits.

#### 6.1.1 Drug Loading into the SCL

Several types of DDSs can be incorporated into contact lenses for sustained release of medication to the eye. Various methods have been studied to develop a contact lens for drug delivery. Each DDS has its advantages and disadvantages, depending on the specific therapeutic need and desired release profile. The selection of DDS for a given condition will depend on various factors, such as the nature of the drug, the duration of treatment, and the desired release profile.

##### 6.1.1.1 Direct Incorporation

Direct incorporation is a technique used to load drugs or other therapeutic agents into contact lenses for sustained and controlled release. It involves the direct addition of the drug to the lens material during the manufacturing process. In this method, the drug is added to the premonomer mixture before polymerization, and the drug becomes uniformly distributed throughout the lens material (Figure 21b). The drug, in the form of a liquid or gel, is stored within the reservoir or microfluidic channels of the lens. Controlled release mechanisms, such as osmotic pumps or electrochemical actuators, are employed to regulate the release of the drug over a specific period. Wang et al. dissolved levofloxacin solution in a nanogel, which is the main material used to form a soft contact lens.^396^

Direct incorporation of drugs into contact lens material has several advantages. First, it allows for the controlled release of the drug, which helps maintain a consistent therapeutic effect. Second, it offers convenience to patients since they do not need to worry about administering the drug separately, which can improve compliance. In addition, the drug is protected from degradation and instability that can occur when exposed to external factors, such as light or air. However, direct incorporation also has some disadvantages. First, only certain drugs can be incorporated, limiting the range of drugs that can be delivered this way. Second, the addition of drugs can decrease the oxygen permeability of the contact lens, leading to discomfort and corneal damage over time. Finally, direct incorporation can increase the cost of the lenses.

##### 6.1.1.2 Soaking Method

The soaking method is a technique used to load drugs onto contact lenses by immersing them in a drug solution. The soaking method has several advantages, including flexibility in being able to load a variety of drugs onto lenses, reduced impact on oxygen permeability, and affordability. The process begins by preparing a solution that contains the drug at the desired concentration. The SCLs are then placed in this solution and allowed to soak for a specific period. During the soaking process, the drug molecules diffuse into the lens material, permeating its structure.

Goswami et al. reported hydrogel-based contact lenses with Avastin, which can be used to treat diabetic retinopathy.^397^ They soaked poly(2-hydroxyethyl methacrylate) hydrogel lens in Avastin (350 μg mL^–1^) containing PBS solutions for 24 h. After the soaking process, the hydrogel lens exhibited no significant change in transparency. Furthermore, it was observed that the soaking resulted in a drug release retention time of approximately 1500 min.

The soaking method also has several disadvantages, including inconsistent drug release, the risk of bacterial contamination during the soaking process, longer loading time compared to direct incorporation, and the need for patient compliance in soaking lenses in the drug solution before use.

##### 6.1.1.3 Nanocomposite-Based Method

A drug delivery system for contact lenses using nanocomposites involves adding nanoparticles containing drugs to the material of the lenses. These nanoparticles may be nanospheres, with drugs evenly spread out in a polymer matrix, or nanocapsules, containing a drug core and a polymer shell. The nanoparticles typically are added to the lenses by soaking them in a solution or mixed with the polymer matrix. The advantage of using this kind of drug delivery system is that it can release drugs for a longer time than traditional methods. The rate and length of drug release can be adjusted by changing the amount and type of polymer matrix and drug used.

Maulvi et al. modulated the drug release by changing the polymer/drug ratio used to produce nanocomposites.^398^ By increasing the ratio of ethyl cellulose to timolol maleate from 1:1 to 3:1 by weight, the dissolution of timolol maleate was prolonged, and the sudden burst effect was reduced. When the 3:1 ratio of ethyl cellulose to timolol maleate nanoparticles was loaded into ring implants and then incorporated into hydrogel contact lenses, the release of the drug was further prolonged.

Micelles are an emerging ocular vehicle that consists of core/shell structures formed by self-assembly. These structures are generated by the dispersion of amphiphilic molecules, which contain both hydrophobic and hydrophilic compounds in a single solution. Polymer micelles have high stability and are capable of encapsulating hydrophobic compounds in the core, promoting controlled or targeted release. Incorporating drug-loaded micelles into contact lenses has shown to be advantageous in controlling drug release, increasing corneal permeability and bioavailability of ophthalmic drugs, and improving wettability, lubrication, and comfort. Researchers have conducted studies to reduce the release of drugs from contact lenses by enclosing micelles containing drugs in the center of cross-linked micelles before adding them to the hydrogel network.^399−401^ This approach has been shown to promote the prolonged release of both model compounds and anti-inflammatory drugs for at least 14 days and up to 30 days, respectively, without adversely affecting the surface wettability or optical transparency of the hydrogels.

Liposomes are a new type of drug delivery system that can release drugs. They are spherical vesicles made up of phospholipids with a double-layer structure and an inner aqueous cavity. The unique structure of liposomes enables them to deliver both hydrophilic and hydrophobic drugs to specific sites.

As explained, nanocomposite-based loading has several advantages in drug delivery through contact lenses, including controlled drug release, enhanced drug loading capacity, reduced risk of toxicity, and improved biocompatibility. However, the technique also presents some drawbacks, such as complexity, higher production cost, limited drug compatibility, and the potential for decreased oxygen permeability.

##### 6.1.1.4 Molecular Imprinting

In contact lens technology, molecular imprinting is utilized to design a drug delivery system that can selectively recognize and attach to a specific molecule or drug. The molecular imprinting method in SCLs involves the creation of molecularly imprinted polymers within the lens matrix. This method utilizes specific functional monomers that interact with target molecules through chemical interactions, resulting in the formation of imprinted sites. After polymerization and template removal, the molecularly imprinted polymer is integrated into the contact lens.^385^ The imprinted sites within the lens selectively bind to the target molecules, enabling molecular recognition and detection. This technique enhances the capabilities of smart contact lenses for applications such as drug delivery, biosensing, and personalized healthcare.

Molecularly imprinted contact lenses have the potential to be used in a variety of applications, including the treatment of various eye diseases, such as glaucoma, cataracts, and dry eye syndrome. Alvarez-Lorenzo et al. developed imprinted hydrogel thin films that were produced for timolol, which is used to treat glaucoma.^402^ Poly(hydroxyethylmethacrylate) hydrogels were used to produce imprinted swollen films capable of releasing timolol for up to 6–10 h. The gels containing 100 mM methacrylic acid had the highest loading levels of 12 mg timolol g^–1^ dry hydrogel.

They also can be used for the delivery of other drugs and molecules, such as antibiotics or anti-inflammatory agents, in a controlled and targeted manner. While the technology is still in its early stages of development, molecular imprinting has shown promise as a powerful tool in the development of advanced DDSs for contact lenses.

#### 6.1.2 Methods of Drug Release

Numerous methods exist for loading drugs into contact lenses, and the means of drug release also exhibit significant variability. Factors such as pressure, heat, magnetic field, and electrical stimulation can serve as triggers for drug release, and their regulation can influence the timing and duration of drug release.

##### 6.1.2.1 Pressure-Triggered Drug Release

**Figure 22:**
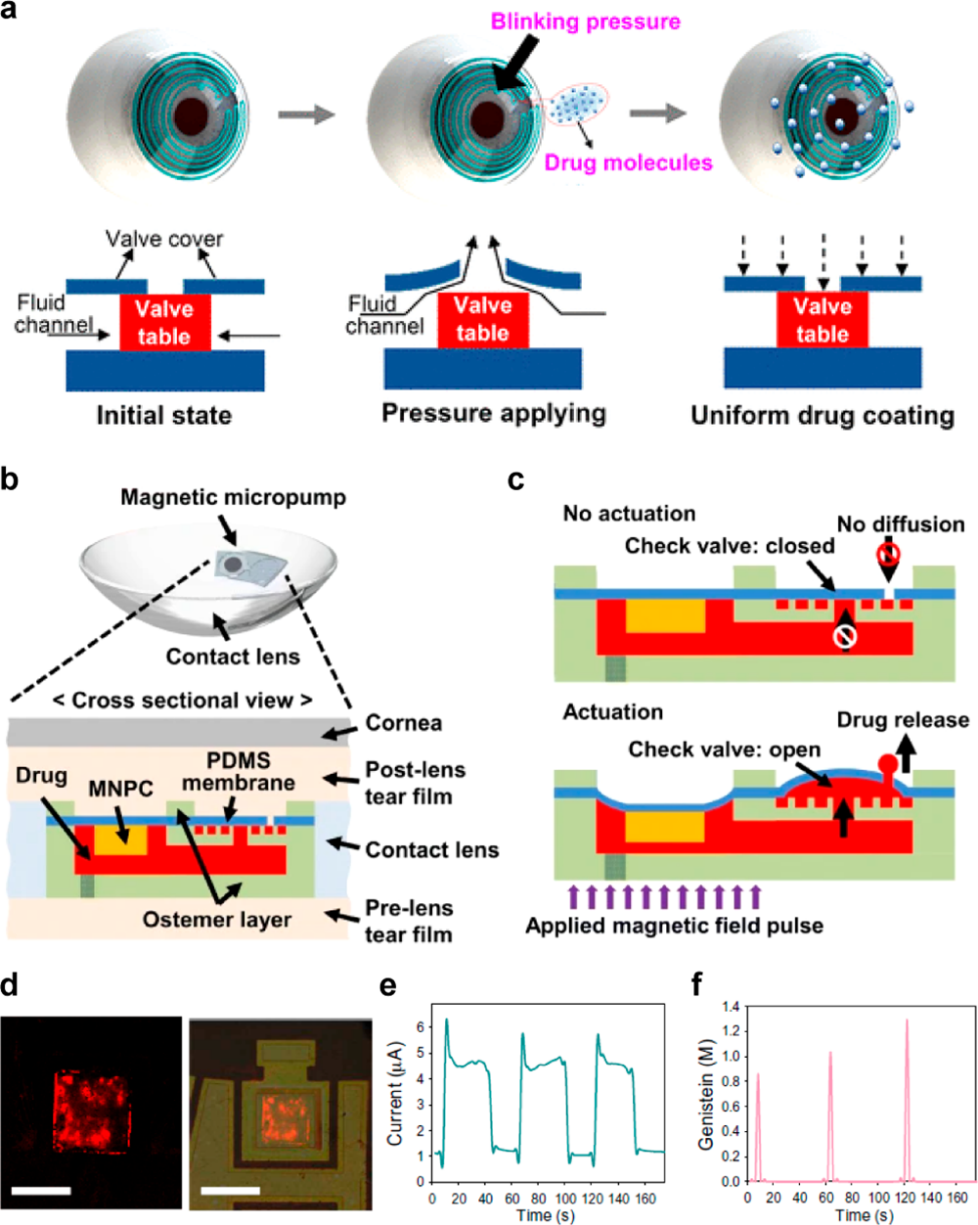
Therapeutic SCL using drug delivery system. (a) Pressure-triggered DDS in contact lens. Reproduced with permission from ref (404). Copyright 2022 American Chemical Society under CC BY 4.0 (https://creativecommons.org/licenses/by/4.0/). (b) Schematic illustration of the magnetic micropump integrated in contact lens. (c) The working process of the micropump, the status of the micropump under the magnetic field pulse, respectively. (b, c) Reproduced with permission from ref (405). Copyright 2020 Springer Nature under CC BY 4.0 (https://creativecommons.org/licenses/by/4.0/). (d) Confocal fluorescence microscopic images of rhodamine B dye released from drug reservoirs. Scale bars, 300 μm. (e) Current change between anode and cathode electrodes of the DDS. (f) Released concentration of genistein. (d–f) Reproduced with permission from ref (245). Copyright 2020 American Association for the Advancement of Science under CC BY-NC 4.0 (https://creativecommons.org/licenses/by-nc/4.0/).

Pressure-triggered drug release in SCLs involves the use of pressure-sensitive materials embedded with drug-loaded microcapsules. When pressure is applied to the lens, such as during blinking or eye movement, the pressure-sensitive material undergoes changes, leading to the release of medication from the microcapsules. This mechanism enables controlled and localized drug delivery directly to the eye, providing therapeutic efficacy and reducing the need for frequent application of eye drops or systemic drug administration. Du et al. conducted experiments to confirm that the release of liquid can be controlled.^403^ They investigated the opening pressure of the outlet check valve and conducted liquid flow tests. They developed a microchannel and circular micropump to ensure effective drug release. Rapidly pressing the circular pump chamber led to increased pressure inside the chamber due to the resistance in the serrated flow path. This increase in pressure would subsequently cause the check valve to open and release the drug preloaded in the reservoir. Figure 22a demonstrates three different states of the check valve, i.e., 1) equilibrium when the internal pressure and the external pressure are the same, 2) opening of the valve when chamber pressure is increased, and 3) rebound of the valve cover to close the valve after pressure is released. Pressure-based drug delivery has several benefits, including controlled release of drugs, no effect on oxygen permeability, and reduced risk of contamination. However, pressure-based DDSs, despite their potential benefits, do have some drawbacks. These include limited drug compatibility and loading capacity, as not all medications may be suitable for delivery through this mechanism. The rigidity of pressure-based systems may also limit the flexibility and comfort of the SCLS, potentially causing inconvenience for patients. Moreover, these systems may be sensitive to external factors such as blinking or eye movements, which can affect the consistency and accuracy of drug release.

It is important to address these limitations through ongoing research and development to improve the effectiveness and user experience of pressure-based DDSs in SCLs. By optimizing drug compatibility, enhancing loading capacity, improving flexibility, and minimizing sensitivity to external factors, the potential of these systems can be fully realized in providing effective and convenient drug delivery for patients.

##### 6.1.2.2 Magnetically Triggered Drug Release

The mechanism of magnetically triggered DDSs in SCLs involves the incorporation of magnetic nanoparticles and responsive materials into the lens design. The contact lens is engineered with magnetic nanoparticles either embedded within the lens material or attached to the surface. When an external magnetic field is applied, the magnetic nanoparticles respond by generating localized forces and movements. This movement triggers the release of the drug encapsulated within the responsive material, such as a hydrogel or polymer matrix. The drug is released in a controlled manner as the responsive material undergoes changes in its physical properties, such as swelling or the opening of nanopores, in response to the magnetic field.

Wang et al. devised a magnetic micropump embedded in a contact lens for drug delivery.^404^ The micropump consisted of several layers, including a top layer for transferring membranes, a functional thin PDMS membrane for the check valve and actuator, a composite of magnetic nanoparticles and PDMS (MNPC) for actuation, a middle chamber for loading drugs, and a bottom cover with an opening for loading drugs (Figure 22b). The top layer was positioned toward the cornea, allowing drug release into the postlens tear film between the lens and cornea. The micropump also featured a micro check valve, consisting of an elastic thin PDMS membrane and a rigid thick Ostemer layer, for one-way drug delivery. The thin PDMS membrane and the thick Ostemer layer had openings in different locations and sizes. The deflection of the thin PDMS membrane was regulated by a magnetic field acting on the MNPC, which resulted in the release of drugs from the openings. If there is no magnetic field present, the Microcheck valve will remain closed and there will be no deflection. This will prevent the diffusion of drugs in an undesired manner (Figure 22c, top). In contrast, when the external magnetic field is activated, the MNPC moves due to the applied magnetic field, causing the deflection of the PDMS membrane (Figure 22c, bottom). This results in an increase in internal pressure, which in turn pushes the micro check valve open. Consequently, the release of drugs is achieved. This method offers a noninvasive, remote-controlled way of delivering drugs to the eye, with the potential for precise dosing and targeted delivery.

However, magnetically triggered drug release systems also have some drawbacks. One limitation is the limited depth of penetration of magnetic fields, which may restrict their effectiveness in reaching specific target areas within the eye. This can potentially hinder precise and targeted drug delivery. Additionally, magnetic field-based DDSs often require external devices or equipment to generate the necessary magnetic field. This requirement can introduce complexity and inconvenience for the user, as it involves the need for additional equipment or setups.

##### 6.1.2.3 Electrically Triggered Drug Release

The mechanism of an electrically triggered drug delivery system in SCLs involves the integration of electrically responsive materials and drug reservoirs into the lens structure. The contact lens is designed with responsive polymers or hydrogels that can change their physical properties, such as swelling or ion exchange, in response to electrical stimuli. Electrically conductive materials, such as electrodes or wires, are also incorporated into the lens. When a voltage or current is applied through the electrodes, the responsive material responds by altering its structure or permeability, thereby releasing the drug from the reservoir.

Kim et al. designed a wireless theranostic SCL to detect IOP and treat glaucoma.^259,252^ The researchers created a gold channel within a contact lens that could be dissolved selectively when a voltage of 1.85 V was applied in a phosphate-buffered saline (PBS) solution. When applying electric current for drug release, it is important to consider the safety of the procedure, particularly when it involves the eye. The actual operation current for drug release in this case is 5–9 μA, which has been determined to be within a safe range that should not adversely affect the eyeball. It is crucial to ensure that the minimum current required to dissolve the gold channel flows while still maintaining safety. In the same manner, Keum et al. reported flexible DDS-laden Genistein with rhodamine B dye (red), as shown in Figure 22d.^245^ The gold channel was dissolved completely as a result of the electrochemical reaction between gold and Cl^–^ in either the PBS or physiological fluid. The current flowing across the cathode and anode of the gold channel was approximately 6 μA. As the current between the anode and cathode electrodes increased, Genistein was released from the gold channel (Figures 22e and 22f). Similarly, Yang et al. induced drug delivery via iontophoresis.^405^ Iontophoresis is a technique for delivering drugs noninvasively that involves using an electric current to increase the permeability of charged drugs or other molecules through biological membranes. In this method, the drug is delivered through the skin or other tissues using an electrode with the same charge as the drug.

One potential disadvantage of electrically triggered drug delivery in contact lenses is that it requires a power source, such as a battery, to provide the electric current necessary to trigger the release of the drug. This can increase the size and weight of the contact lens and make it less comfortable for the wearer. In addition, there is a risk of electrical shock if the power source malfunctions or the contact lens is damaged.

It is important to note that even a well-made lens can potentially cause damage to the eye depending on the intensity of the applied current. In the recent research, the current between reference electrodes in experiments has generally not exceeded 10 μA, and the voltage conditions have not exceeded 6 V. To ensure safety, researchers should conduct initial tests to determine the minimum current and voltage conditions required for electrode dissolution. Subsequently, *in vivo* experiments should be performed to thoroughly assess whether the technique poses any risks of eye damage. By following this sequential approach, researchers can carefully evaluate the parameters and potential effects on ocular tissues, ensuring the well-being of the subjects involved in the study.

##### 6.1.2.4 pH-Triggered Drug Release

The pH-triggered drug delivery in contact lenses refers to the release of drugs from a contact lens in response to changes in pH levels. Typically, the drug is embedded in a hydrogel material that is sensitive to changes in pH, and the release of the drug is triggered when the pH level of the surrounding environment changes. When the pH of the surrounding environment, such as the tear film on the surface of the eye, reaches a specific threshold, the pH-sensitive material undergoes swelling, dissolution, or changes in permeability. For example, if the pH level of tears changes due to an infection or disease, the hydrogel in the contact lens can release the drug to treat the condition. In general, the pH level increases when the eyeball becomes inflamed due to injury or surgery.^406^ When this happens, the hydrogel in the contact lens can release the drug to treat the condition.

A pH-triggered, controlled drug release from contact lenses also can be promoted by preparing nanoparticles based on pH-sensitive polymers, such as Eudragits. Maulvi et al. presented Eudragit-based contact lenses that were laden with nanocomposites.^398^ The nanocomposite demonstrated promising results as it continuously released the drug when Eudragit S100 was dissolved in tear fluid with a pH of 7.4. By adjusting the weight ratio of the nanocomposites and Eudragit, the drug release could be extended up to 156 h without any impact on the optical and physical properties of the contact lens.

This type of drug delivery system is advantageous because it can provide sustained and controlled drug release, reducing the need for the frequent administration of medication. In addition, the drug is delivered directly to the affected area, thereby minimizing systemic side effects. However, variations in the pH of individuals’ tears or ocular environment can affect the reliability and consistency of drug release in pH-responsive systems.

##### 6.1.2.5 Heat-Triggered Drug Release

The mechanism of a heat-triggered drug delivery system in SCLs involves the incorporation of heat-responsive materials and drug reservoirs into the lens structure. The contact lens is designed with heat-responsive polymers or hydrogels that change their physical properties, such as swelling or sol–gel transitions, in response to changes in temperature. When the temperature reaches a specific threshold, typically close to body temperature, the heat-responsive material undergoes a phase transition, leading to the release of the drug from the reservoir. This heat-triggered drug release mechanism enables controlled and targeted delivery of medication to the eye, with the drug being released in response to the natural heat of the eye or through external heating sources.

When exposed to heat, heat-responsive materials undergo a reversible phase transition that alters their physical properties. The transition can be triggered by a range of thermal stimuli, including temperature changes, IR radiation, or near-infrared (NIR) light. This transition can cause a change in the size, shape, or permeability of the materials, allowing it to release a drug or a bioactive molecule in response to a specific temperature.

A common example of a heat-responsive material used in SCLs is a hydrogel. One advantage of using heat-responsive hydrogels is their ability to release drugs or bioactive molecules in a controlled manner, which can minimize side effects and improve therapeutic outcomes. These hydrogels also can be designed to respond to specific temperatures, making them ideal for targeted drug delivery or tissue engineering applications.

However, heat-triggered drug release systems come with certain disadvantages. One primary concern is the need to carefully manage the amount of heat generated to prevent thermal damage to ocular tissues. Uncontrolled or excessive heating can lead to discomfort, tissue injury, and other adverse effects. It is crucial to ensure precise control over the heat delivery process to maintain the safety and well-being of the patient.

Additionally, not all drugs are suitable for heat-based delivery. Some medications may be sensitive to heat and can experience a loss of efficacy or stability when exposed to elevated temperatures. This limitation must be considered when designing heat-triggered drug release systems to ensure that the selected drugs remain effective and maintain their desired properties during the delivery process.

To overcome these challenges, ongoing research and development efforts focus on refining heat-based DDSs to achieve precise temperature control, minimize the risk of thermal damage, and optimize drug compatibility. By addressing these concerns, heat-triggered drug release systems can become safer, more efficient, and compatible with a broader range of medications, enhancing their potential as therapeutic tools in SCLs technology.

In addition to the aforementioned examples, various drugs can be incorporated into contact lenses. To provide our readers with more comprehensive information, we have compiled a list of drugs that can be contained in contact lenses (Table 8).^245,259,407−415^

**Table 8:** Types of the Therapeutic SCLs Using Drug Delivery

Drug	Incorporation method	Indication	Trigger	Reference
Timolol	Casting	Glaucoma	Electrical stimulation	(259)
Timolol	Nanocomposite-based	Glaucoma	Diffusion	(407)
Resveratrol	Soaking	Anti-inflammatory	Diffusion	(408)
Melatonin	Soaking	Dry eye	Diffusion	(409, 410)
Brimonidine tartrate	Direct incorporation	Glaucoma	Electrical stimulation	(405)
Doxorubicin	Soaking	Postoperative complications	Diffusion	(411)
Bevacizumab	Molecular imprinting	Anti-inflammatory	Diffusion	(412)
Levofloxacin	Direct incorporation	Bacterial infections	Diffusion	(397)
Atorvastatin	Soaking	Dry eye, blepharitis	Diffusion	(413)
Genistein	Casting	Diabetic retinopathy	Electrical stimulation	(245)
Avastin	Casting	Diabetic retinopathy	Electrical stimulation	(245)
Diclofenac sodium	Soaking	Anti-inflammatory	pH-triggered	(414)
Loteprednol etabonate	Direct incorporation, nanocomposite-based	Anti-inflammatory	Diffusion	(415)

### 6.2 Heat Therapy

The tear fluid consists of three layers, i.e., a lipid layer, an aqueous layer, and a glycocalyx layer. The meibomian gland secretes the lipid layer, which covers the aqueous layer and prevents the moisture from evaporating. If the gland is damaged, it leads to hyper-evaporative dryness on the surface of the eye, causing MGD, which is the primary cause of OSI. The normalization of gland function can be achieved by treating the eyelids with heat therapy. However, conventional thermal therapy equipment is not convenient for regular use because of its bulky components and obscured vision. For instance, medical devices, such as Lipiflow thermal pulsation devices and intense pulse light treatments, are effective for OSI, but they are only applicable in clinical settings as they are bulky and obstruct the user’s vision.

The integration of a heating element in contact lenses is an innovative approach that has garnered significant attention due to its potential therapeutic benefits. Heated contact lenses can treat a variety of eye conditions, such as dry eye syndrome, meibomian gland dysfunction, and corneal disorders. These lenses provide localized heat therapy to the eye, which can enhance blood circulation, increase tear production, and alleviate associated symptoms. In addition, this technology has promising applications in ocular drug delivery and biosensing. The development of heater-equipped contact lenses has opened up new avenues for the diagnosis, treatment, and prevention of various eye diseases.

Jang et al. reported that a heater wirelessly connected with a contact lens could be a convenient and noninvasive method for treating inflammation of the ocular surface.^240^ The contact lens was integrated with the NFC chip to measure the concentration of MMP-9 in real-time, and the heat patch connected to the Bluetooth received signals from the outside for the treatment of chronic ocular surface inflammatory disease. The system was tested in a small group of patients with chronic OSI, and the results showed that the SCL with heat patch were effective in reducing inflammation and improving symptoms.

### 6.3 Optical Therapy

Light-mediated therapy offers the advantage of noninvasively improving oxidative stress in retinal cells, which has led to increased interest in utilizing this approach to treat ocular disorders, including AMD. Light-stimulated contact lenses have been developed to use light-mediated therapy for improving eye function and enhancing the treatment of ocular conditions. The duration and intensity of light exposure are critical factors in phototherapy. While the exact exposure time and intensity vary depending on the specific treatment, it is essential to ensure that the light levels are within safe limits to prevent any potential harm to the eyes. Prolonged exposure or excessive intensity can potentially damage the delicate structures of the eye. Prolonged exposure or excessive intensity can potentially damage the delicate structures of the eye. Phototherapeutic modalities use red light within the wavelength range of 630–680 nm, which allows deep-tissue penetration without causing adverse effects at effective light intensities. This light stimulates the cytochrome C oxidase in mitochondria, promoting protein synthesis and the release of adenosine triphosphate (ATP), activating the lymphatic system, and reducing oxidative stress within human cells.^416,417^

**Figure 23:**
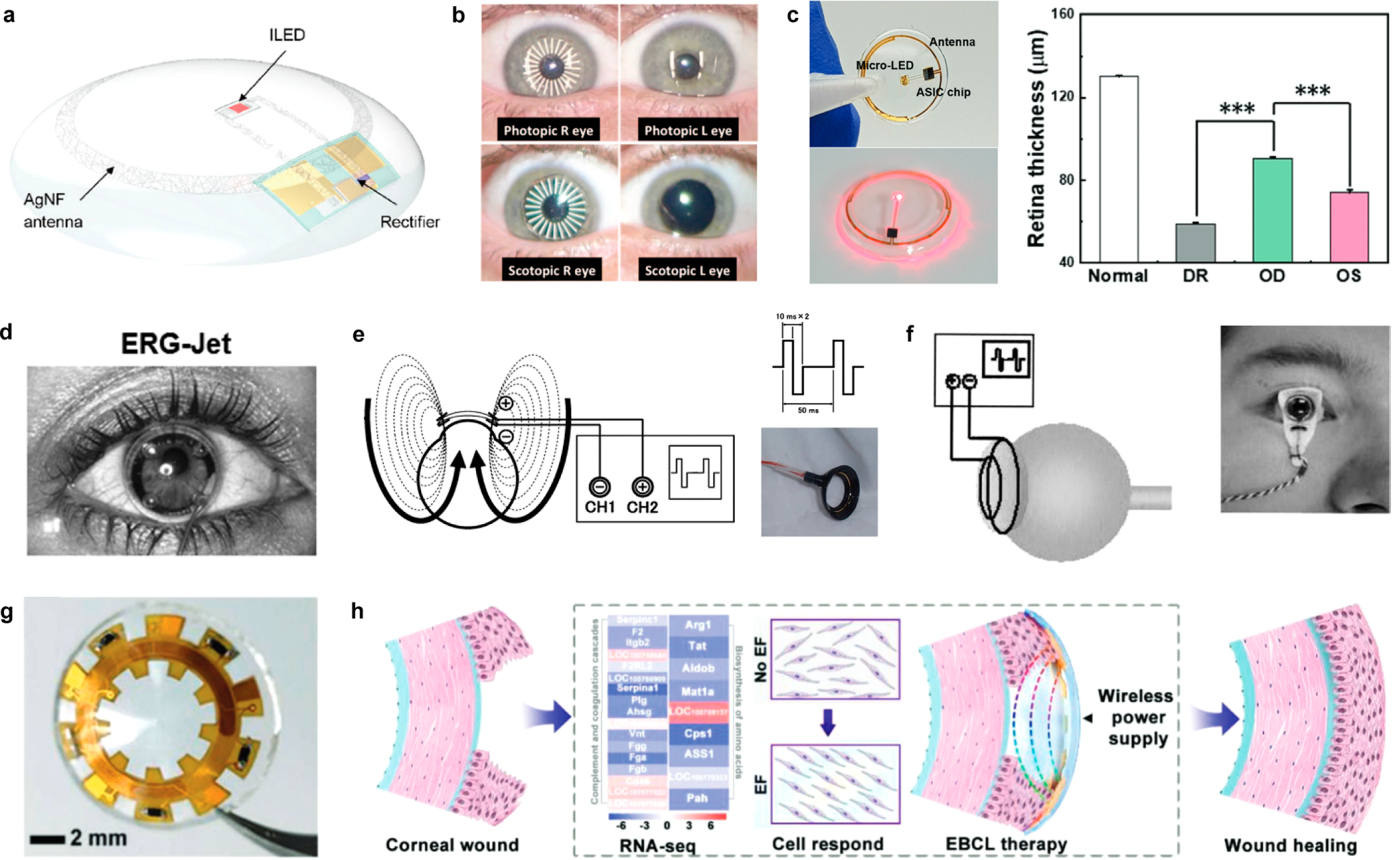
Smart contact lenses as photobiomodulator for optical and electrical stimulation therapy. (a) Schematic of wireless phototherapeutic contact lenses. Reproduced with permission from ref (418). Copyright 2019 Springer Nature. (b) Photographs of eyes with phototherapeutic lenses under photopic and scotopic conditions. Reproduced with permission from ref (422). Copyright 2018 IEEE. (c) Photograph of smart LED contact lens (left), retina thickness of the normal, diabetic retinopathy, oculus dextrus (OD) and oculus sinister (OS) (right). Reproduced with permission from ref (250). Copyright 2022 John Wiley and Sons under CC BY 4.0 (https://creativecommons.org/licenses/by/4.0/). (d) Photograph of ERG-Jet electrode (photo courtesy of Fabrinal SA). Reproduced with permission from ref (424). Copyright 2011 IEEE. (e) Schematic diagram of the pathways of the electrical stimulation therapy by contact lens. Reproduced with permission from ref (427). Copyright 2007 Springer Nature. (f) Schematic diagram of transcorneal electrical stimulation (left). Photograph of the Burian-Allen contact lens electrode (right). Reproduced with permission from ref (428). Copyright 2006 Springer Japan KK. (h) Schematic of accelerated corneal wound recovery by the electrical bandage contact lens. (g, h) Reproduced with permission from ref (429). Copyright 2022 John Wiley and Sons under CC BY 4.0 (https://creativecommons.org/licenses/by/4.0/).

Numerous studies have been conducted to explore alternative materials and designs to create thin, breathable, and flexible phototherapeutic contact lenses. Park et al. presented a platform that potentially could be effective for ophthalmologic diseases, such as AMD, by demonstrating a glass-type device and a contact lens-type device (Figure 23a).^418^ A contact lens with red inorganic light-emitting diodes (ILED) was developed by miniaturizing the pixels and placing them at the boundary of the pupil. This design facilitates unobstructed viewing for the user while potentially allowing the red light emitted by the ILEDs to reach the optic nerve and provide therapeutic effects. In addition, a built-in antenna was included to control the light intensity and enable wireless operation. An *in vivo* test was conducted on a live rabbit to confirm the safety of the device in terms of heat generation, which could potentially cause harmful effects to the eye. The study confirmed that the device maintained a safe temperature of 37 °C, thereby demonstrating its efficacy and reliability as a phototherapeutic device.

Recent studies have highlighted concerns regarding the safety of phototherapy, leading to suggested restrictions on exposure time and intensity levels. Based on these studies, it is currently recommended to limit the maximum exposure time of phototherapy in SCLs to 2 h and maintain an intensity level of 200 cd m^–2^.^418^ These recommendations aim to minimize the potential risks and adverse effects associated with prolonged or excessive exposure to light therapy.

Photobiomodulation also has been used in research to prevent and treat diabetic retinopathy, which is one of the leading causes of blindness in adults. The primary symptom of diabetic retinopathy is the leakage of blood from weakened blood vessels. Elevated blood glucose levels can cause damage to the microvasculature of the retina, leading to reduced blood flow and oxygen supply to the retinal cells. This can result in retinal edema and hypoxia-induced damage to the capillaries.^419^ In response to this damage, retinal cells release signaling molecules that promote the formation of new blood vessels. However, these newly formed blood vessels often are abnormal and fail to establish a functional blood-retina barrier, leading to the leakage of blood and fat into the retina and causing visual impairment.^420^ Thus, it is crucial to prevent the generation of new blood vessels and to promote the expansion of existing blood vessels.

IR and NIR light in the NIR spectrum can induce the production of nitric oxide (NO) in retinal endothelial cells through a photochemical reaction.^250,421^ When retinal endothelial cells are exposed to IR and NIR light, photons of specific wavelengths and energies are delivered, which are absorbed by hemoglobin, an oxidized form of hemoglobin present in red blood cells within the vascular endothelial cells. Upon exposure to light, hemoglobin undergoes a temporary conversion into yellow hemoglobin, and during this process, yellow hemoglobin absorbs red and NIR light, leading to the generation of hydroxyl radicals. These hydroxyl radicals induce the oxidation of NO in vascular endothelial cells, resulting in the production of NO. NO is a vital signaling molecule known to play various physiological roles in vascular endothelial cells, including vasodilation, blood pressure regulation, inflammation inhibition, and blood coagulation inhibition.

By harnessing these principles, IR and NIR light irradiation can aid in the expansion of retinal microvessels and the inhibition of new blood vessel formation. Consequently, research is currently underway to explore the use of light irradiation for the prevention and treatment of diabetic retinopathy. Common approaches involve noninvasive techniques such as light irradiation, laser irradiation, intraocular injection, or surgery to coagulate or inhibit the formation of new blood vessels in the retina.

As an alternative to current treatment methods, Cook et al. developed a phototherapeutic contact lens that can be used for the treatment of diabetic retinopathy (Figure 23b).^422^ The contact lens is designed to emit NIR light and reduce retinal metabolism by suppressing rod cell dark current, which can help to protect retinal cells from the damage caused by oxidative stress. Lee et al. developed a wireless contact lens that emits near-infrared light for the treatment of diabetic retinopathy.^250^ This contact lens incorporates a far red/NIR LED and a wireless power transfer system, including an application-specific integrated circuit chip and an antenna (Figure 23c, left). In addition, a Bluetooth module is integrated to regulate the transmission frequency and power of radiofrequency waves to a smartphone. In rabbit experiments, it was confirmed that the retinal vascular permeability, an angiogenic factor, was reduced, and demonstrating safety through the absence of corneal damage caused by heat generation and maintenance of corneal thickness (Figure 23c, right). These phototherapeutic contact lenses enable continuous noninvasive ophthalmological treatments and have significant potential as an ophthalmic healthcare platform, thus further research is warranted in this area.

### 6.4 Electrical Stimulation

Recent research has been focused on developing contact lenses that deliver electrical stimulation to improve eye health, including enhancing visual function, activating cortical plasticity, and promoting the healing of wounds. The minimally invasive electrical stimulation therapy at the cornea level presents fewer side effects, e.g., mild corneal punctate keratopathy and a lower risk of complications.^423^

As a type of lens-based device, the ERG-jet electrode is a hard contact lens electrode primarily designed for delivering electrical stimulation to the retina (Figure 23d).^424^ The device includes a contact lens with a golden foil attached to either its inner surface (monopolar) or outer surface (bipolar).^425^ Morimoto et al. demonstrated the improvement in visual function through transcorneal electrical stimulation (TES) using contact lenses.^426^ The authors conducted an *in vivo* test using rats with optic nerve damage, and they found that TES therapy increases an insulin-like growth factor (IGF)-1 in the retina, which is a neurotrophic factor that promotes the survival and regeneration of RGCs. The study suggests that TES delays and may even prevent the degeneration of retinal neurons. Xie et al. developed a mathematical model of TES, and they predicted that stimulation using an ERG-jet electrode could enhance visual perception by activating a wide range of RGCs that transmit visual information to the brain.^424,407^ Furthermore, clinical studies have confirmed that TES using the ERG-jet electrode leads to the activation of the visual cortex.

Electrical stimulation treatment of these contact lenses is used as a means of improving the visual function of patients with ophthalmic diseases. Inomata et al. conducted a study in which a patient with long-standing retinal artery occlusion underwent electrical retinal stimulation therapy using a bipolar contact lens electrode (Figure 23e).^427^ The result of the study demonstrated that, following the treatment, the patient exhibited improvements in visual acuity and field, as well as an increase in multifocal ERG responses. Several studies have investigated the potential correlation between TES and visual function in patients with nonarteritic ischemic optic neuropathy (NAION) or traumatic optic neuropathy (TON) (Figure 23f).^428^ A bipolar Burian-Allen contact lens showed that TES therapy improved visual acuity, and this suggested that electrical stimulation therapy using a contact lens could be a safe and effective treatment option.

As the research into the effectiveness of electrical stimulation in promoting the healing of wounds and supporting vision recovery advances, contact lens-type devices that can deliver electrical stimulation are being developed. Wu et al. developed a wireless-powered electrical bandage contact lens to promote recovery from wounds by applying a localized electric field to a rabbit’s corneal injury (Figure 23g).^429^ The researchers demonstrated that the electric field generated by the contact lens can stimulate cell migration, proliferation, and differentiation, as well as enhance the secretion of growth factors (Figure 23h). After applying electrical stimulation treatment to rabbits with corneal epithelium defects through EBCL for 3 days, complete healing of wounds was observed, whereas the control group showed remaining epithelial defects.

Although electrical stimulation therapy through a contact lens-type device has been reported to protect deteriorating retinal cells and improve visual function, it is still a relatively new treatment modality for ophthalmic disease. However, further investigation is necessary to establish the safety and efficacy of this therapy for the treatment of ophthalmic diseases.

The therapeutic potential of SCLs is vast by considering the ability of SCLs to provide real-time monitoring and treatment of eye-related diseases through the integration of various existing technologies. These lenses can be equipped with advanced features such as DDSs and microelectronics to detect physiological changes (such as intraocular pressure, tear composition, and glucose levels) and to perform targeted therapy. Continuous monitoring and management of these lenses can significantly improve patient outcomes, increase treatment adherence, and reduce healthcare costs.

Moreover, SCLs can provide additional therapeutic functions, including heat therapy, optical therapy, and electrical stimulation, alongside their drug delivery capabilities. Hyperthermia involves the use of a built-in heating element to provide local heat treatment to the eye to stimulate blood circulation, increase tear production, and relieve associated symptoms. Optical therapy utilizes light-emitting devices integrated in contact lenses to improve visual function, activate cerebral plasticity, and aid in the recovery of damaged retinal cells. Also, electrical stimulation, delivered through contact lenses, can help improve vision, promote wound healing, and protect retinal cells by delivering targeted electrical impulses. Although these therapeutic functions of SCLs are very promising, many of these technologies are still in the research and development phase and have not yet been commercialized. Further studies and clinical trials are necessary to ensure safety and efficacy.

## 7 Therapeutic Modalities That Can Be Integrated with SCLs for Ocular Therapy

We have introduced therapeutic modalities and platforms based on SCL that function noninvasively on the outer space of the eye (i.e., the cornea). However, on some occasions, direct stimulation to the retina is required for patients to excite the remaining neurons in the retina with retinal diseases, such as RP and AMD, which cause photoreceptor cells to degenerate, making the retina unresponsive even when light enters the eye. In this section, we introduce other forms of therapeutic modalities that directly stimulate the retina, invasively or noninvasively, thereby restoring their visual function. We envision that these modalities can provide synergetic effects when integrated with SCLs.

### 7.1 Retinal Prosthesis

#### 7.1.1 The Mechanism of Retinal Prosthesis

Retinal prosthesis, which stimulates the retina directly by implanting the device in locations near the retina, can restore visual function through the generation of the unique phenomenon, known as phosphene. Phosphene is used to describe the situation in which visual perception is experienced even though no light enters the eye. It can be implemented through various types of stimulation, i.e., physical, electrical, and magnetic stimuli. The easiest way to experience phosphene is to close your eyes and rub or apply pressure to your eyelids using your fingers. During this process, an individual will be able to feel visual perception in the form of flashing lights with diverse patterns. This is caused by the physical stimulation of the retinal neurons generated from the pressure applied to the eyelids.^430^ Several methods to generate phosphene have been identified (e.g., electric, ultrasound),^431^ but, in general, current retinal prostheses implement the generation of phosphene by electrical stimuli.

The idea of generating artificial vision with electrical stimuli began in 1752 with the theorisations of the eminent Benjamin f, who postulated to the Royal Society of London that sight and hearing could be restored with the use of electricity.^432^ In 1755, Le Roy et al. discovered the electrically induced visual percepts, also known as phosphenes, when they applied an electrical current to a blind patient, who reported seeing visual perceptions.^433−436^ Years later, in 1929, Föerster further demonstrated this phenomenon by showing that acute external stimulation could elicit phosphenes, showing a significant breakthrough in the advance of artificial vision.^430^ Brindley and Lewin took advantage of this knowledge and implanted an electrode to electrically stimulate the visual cortex.^433^ Potts and Inoue further developed the concept by demonstrating that the application of an electrical current to individuals with RP can elicit phosphenes and produce measurable responses from electrodes positioned over the occipital scalp. Overall, these findings illustrate how the understanding of electrically induced phosphenes has evolved and how researchers have utilized this knowledge to develop new technologies for treating visual impairments with retinal prostheses.

**Figure 24:**
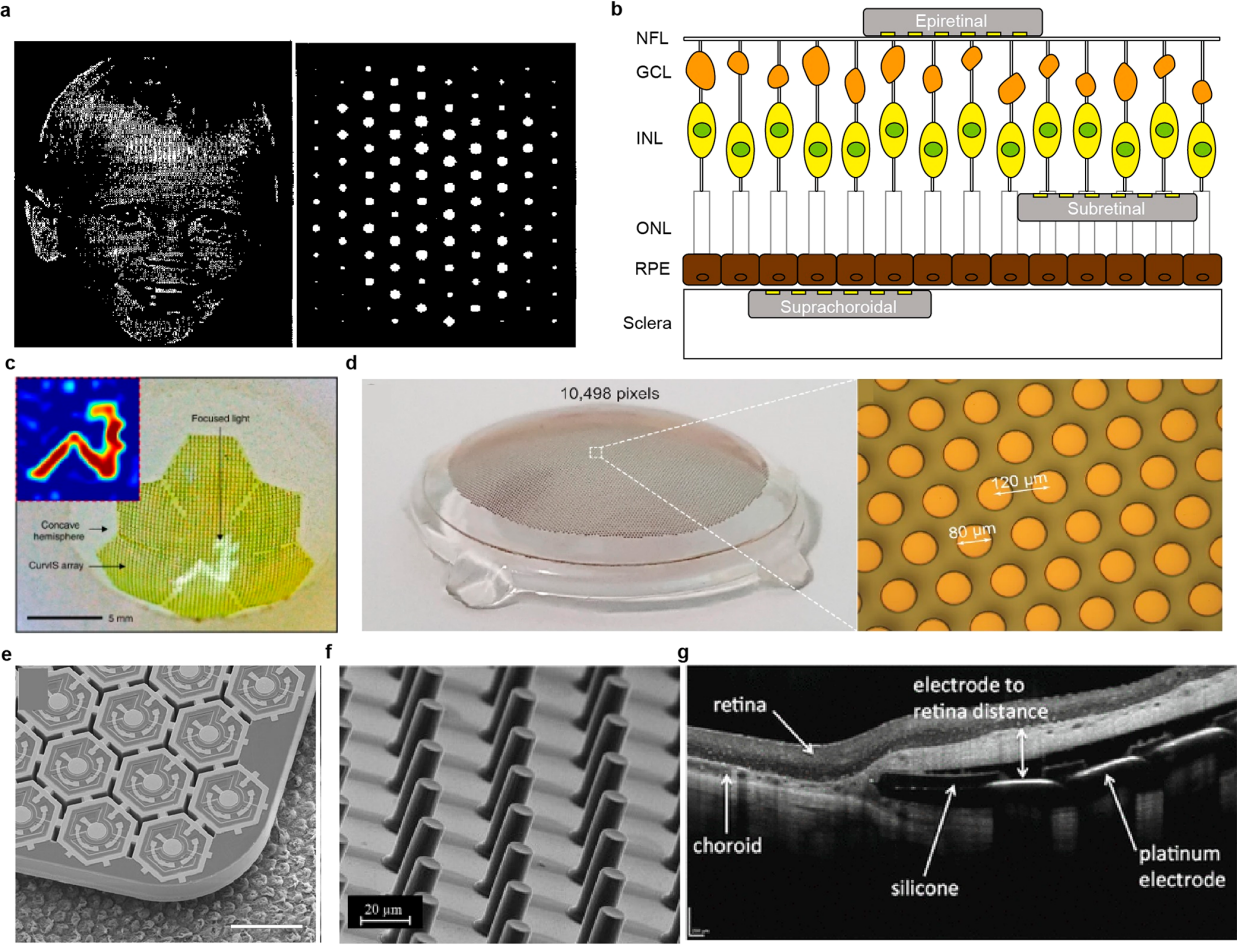
Retinal prosthesis. (a) Original image (left) and the generation of phosphenes according to the image with 100-pixels (right). Reproduced with permission from ref (437). Copyright 2004 IEEE. (b) Schematic of the types of retinal prosthesis classified by their implanted positions. (c) Photograph of an ultrathin optoelectronics with truncated icosahedron design. Scale bar, 5 mm. Reproduced with permission form ref (441). Copyright 2017 Springer Nature under CC BY 4.0 (https://creativecommons.org/licenses/by/4.0/). (d) Photograph of a retinal prosthesis design of wide retinal coverage for the wide angle of view (left), magnified optical microscope image of each pixel (right). Reproduced with permission form ref (442). Copyright 2021 Wiley under CC BY 4.0 (https://creativecommons.org/licenses/by/4.0/). (e) SEM image of photovoltaic pixels for the wireless stimulation of the retina. Scale bar, 100 μm. Reproduced with permission form ref Copyright 2020 Wiley under CC BY 4.0 (https://creativecommons.org/licenses/by/4.0/). (f) SEM image of 3D electrodes of retinal prosthesis. Scale bar, 20 μm. (g) Cross-sectional OCT image of the implanted suprachoroidal prosthesis. (f, g) Reproduced with permission form ref (446). Copyright 2014 Ayton et al. under CC BY 4.0 (https://creativecommons.org/licenses/by/4.0/).

In summary, when the RGCs are stimulated directly by adequate external stimuli, inward movement of sodium ions and outward movement of potassium ions are made through the cell membrane of the cell body (i.e., depolarization), and this is known as the starting phase of the action potential.^435^ When an electrical gradient (i.e., electrical stimulation voltage) occurs near the RGCs with stimulating electrodes, they undergo consecutive steps of depolarization, repolarization, and hyperpolarization, thereby generating action potentials. This neural code, in the form of a spike train (sequence of action potential firing timings), traverses through the optic nerve and is interpreted by the visual cortex, resulting in the perception of vision.^436^ Thereby, stimulating the remaining cells in the retina and generating phosphene at the point where light enters enable patients with RP or AMD to perceive visual information. Although it can only implement colorless white percept, it aims to restore vision to the level where shapes can be distinguished by stimulating the retina with multiple microsized arrays of electrodes (Figure 24a).^437^

Retinal prostheses consist of several components, including a camera system, an image processing unit, a microelectrode array, and a stimulation system. The camera system captures visual information and sends it to the image processing unit, which converts the visual information into electrical signals. Then, the electrical signals are transmitted to the microelectrode array, which is placed near the retina (e.g., epiretinal, subretinal, and suprachoroidal) to deliver these electrical signals to the remaining retinal cells and generate phosphene.

#### 7.1.2 Types of Retinal Prosthesis

The retinal prosthesis can be classified mainly into three types (i.e., epiretinal, subretinal, and suprachoroidal) according to the location of the microelectrode array (Figure 24b and Table 9).

**Table 9:** Characteristics of Retinal Prosthesis According to Position of Microelectrode Array

Type of retinal prosthesis	Epiretinal	Subretinal	Suprachoroidal
Implant position	On the surface of the retinal nerve fiber layer	Between the photoreceptor layer and the retinal pigment epithelium/choroid	Between the choroid and sclera
Major target cells	RGCs	Bipolar cells	Bipolar cells
Stimulation efficacy	Directly stimulation of the RGCs	Directly interfaces the bipolar cells	Low proximity to the target cells
	Low stimulation threshold	Low stimulation threshold	High stimulation threshold
			Spread of current, thereby reducing the spatial resolution
Surgical method	Transvitreal through a pars plana sclerotomy	Vitrectomy, retinotomy	Intravitreal surgery
Surgical limitations	Possibility of retinal detachment or hemorrhage	Risk of damaging healthy photoreceptor cells during insertion	Less invasive compared to other two and does not involve direct contact with the retina
	Requires tacks to fix the prosthesis on the retinal surface	Postsurgical complications such as inflammation or proliferation of tissue over the implant	
	Prosthesis may move or become dislodged, requiring further surgical intervention		

##### 7.1.2.1 Epiretinal Prosthesis

The epiretinal prosthesis is attached to the innermost surface of the retina, adjacent to the optic nerve and the RGCs. Due to its location, the main advantage of the epiretinal prosthesis is that it can stimulate the RGCs directly in close proximity, providing efficient charge injections and reducing stimulation thresholds. Also, its surgical delivery method, transversal through a pars plana sclerotomy, is familiar to surgeons, and revision of the device placement and explanation can be less complex.^438,421^ The device can be attached to the retina by using a tack, which is surgically the best approach for attaching a device to the epiretinal membrane.^439^

The representative epiretinal prosthesis is the Argus II epiretinal prosthesis (Second Sight Medical Products, Inc., Sylmar, USA). It was the first device to receive CE marking, and is the most extensively used retinal prosthesis.^440^

The current state-of-the-art epiretinal prosthesis has evolved with diverse strategies. Choi et al. developed an epiretinal prosthesis utilizing ultrathin optoelectronics and a truncated icosahedron design to minimize the excessive pressure applied to the retina caused by of the mechanical mismatch between the implanted device and the retina, which can damage the retina (Figure 24c).^441^ Ferlauto et al. proposed a unique design of foldable, wide-field, epiretinal prosthesis, by the integration of the PDMS-photovoltaic interface with 2,215 stimulating pixels distributed on an active area of 12.7 mm and the dome-shaped PDMS support, which can be implanted into the eye through a small scleral incision. After the implantation of this device in a folded form, it opens up to a hemispherical shape to match the curvature of the eye and gently adheres to the retina. Chenais et al. developed a retinal prosthesis with photovoltaic materials to enable wireless operation of the prosthesis.^442^ This epiretinal prosthesis consisted of 10,498 physically and functionally independent photovoltaic pixels, allowing for wide retinal coverage and high-resolution stimulation for the wide angle of view (Figure 24d).

Direct stimulation of RGCs can be disadvantageous because it bypasses the residual intraretinal processing system, thereby restricting the ability to replicate the physiological topographic organization of the retina. In addition, the proximity of epiretinal devices to the adjacent axonal nerve fibers may result in ectopic visual percepts arising from the unintended RGC axonal stimulation.

To overcome these limitations, approaches have been developed to locate the retinal prosthesis on the opposite side to stimulate the upper stream of the retinal neurons (i.e., bipolar cells) and facilitate the use of a natural visual processing system.

##### 7.1.2.2 Subretinal Prosthesis

The subretinal implant is based on the rationale to exploit the intrinsic signal processing capacity of the retina by positioning the device in the ONL, where the degenerated photoreceptors are positioned and stimulating the bipolar cells. Furthermore, the device is located closer to the bipolar cells compared to the epiretinal approach, which can utilize the natural retinal signal amplification, resulting in lower stimulation intensities.

The Alpha IMS (Retina Implant AG, Germany), which is the most implanted subretinal implant, received the CE marking, in 2013. The device contains a photovoltaic array known as a multiphotodiode array (MPDA), which consists of a 3 mm^2^ microchip, containing 1,500 independent photodiode-amplifier-electrode units that convert ambient light into an electrical signal. As an active device, it uses an extrinsic power source connected to a subdermal coil. Then, a coil that is attached magnetically to the subdermal coil allows electromagnetic power induction to the active device. While improvement in the object recognition for a couple of months after the implantation with the subretinal device was reported, the best visual acuity is known as 20/546 via the Landolt C-ring testing. However, issues such as RPE degeneration and adhesion to the retina can complicate subfoveal device placement, resulting in more surgeries to reposition or replace the subretinal prosthesis, and device failure in severe cases.

Lorach et al. demonstrated the use of photovoltaics for subretinal implants with 70-μm-wide pixels to stimulate the retina of rats with retinal degeneration wirelessly (Figure 24e).^35,443^ The results enabled the retinal responses with a spatial resolution of 64 ± 11 μm, which corresponds to half of the normal visual acuity in healthy rats. Prévot et al. reported the evaluation of these photovoltaic subretinal prostheses in three awake nonhuman primates using the aforementioned near IR sensitive photovoltaic subretinal prosthesis to stimulate the retina with multipixel stimulation.^444^ The results have shown a promising approach with the fully wireless photovoltaic high-resolution implant activating the blind nonhuman primate retina with a single 100-μm *in vivo*.

Owing to the advances in photovoltaics and device designs, a hexagonal subretinal prosthesis based on photovoltaics was developed by Pixium Vision S.A. that contained 378 electrodes with the size of 2 × 2 mm and a 30-μm thickness. Each pixel has its own local electrical return path to provide more targeted stimulations. A mini camera mounted on the specially constructed glasses captures the visual information and processes it into pulses of NIR to activate the photovoltaics placed on the subretinal space. Then, the electrical current generated within the photovoltaics polarizes the adjacent neuronal tissue, without requiring transscleral wires or additional power induction like in the Alpha IMS. Animal model studies have shown encouraging preclinical results using this approach. With Royal College of Surgeon (RCS) rats, which have degenerated photoreceptors in their late stages, the visual-evoked potential (VEP) generated by the photovoltaic stimulation, which occurs from the visual cortex of the brain, showed latency and amplitude to VEPs in rats with normal visual functions. Although contrast sensitivity was limited, anodic-first biphasic pulses lowered stimulation thresholds of the RGCs and satisfy the limit of ocular safety.

The subretinal devices exhibit significant improvements in major concerns of external interconnections; however, there are still limitations to consider. First, this approach assumes that the anatomical organization of the retinal interneuron network remains intact, which is unlikely to be the case as the remaining RGCs and bipolar cells undergo subtle changes in their structure to large-scale reorganization (i.e., retinal remodeling).^445^ Second, due to the low proximity to the target cells compared to the epiretinal prosthesis. As such, this may require higher power consumptions as well as heat generation, limiting its safety in terms of clinical trials.

To overcome these limitations, several strategies including the fabrication of stimulating electrodes in 3D have been made. Structures such as pillars or concave forms enable them to reach out to the target cells thereby stimulating them in close proximity (Figure 24f).^446^ Also, these 3D structures can enable the target cells to remodel through the concave structure to decrease the distances to the cells.

##### 7.1.2.3 Suprachoroidal Prosthesis

The suprachoroidal retinal prosthesis does not necessitate transvitreal surgery and is therefore potentially less invasive and more easily accessible for repair or replacement, as it is placed in the suprachoroidal space, which is between the sclera and the outer retina/choroid.

Ayton et al. reported the first-in-human trial to investigate the use of retinal implants in the suprachoroidal space in three human subjects with end-stage RP and reported the 12-month postoperative efficacy data (Figure 24g).^447^ The first of these was a 24-channel system, consisting of 20 stimulation channels and 4 return electrodes. This system involves the dissection of the temporalis muscle for attachment of a percutaneous connector to the bone, likewise to the Alpha-IMS. In 2012, a pilot study was conducted on three subjects with advanced RP, in which a device that relies on a camera and an image processor for the stimulation pattern of the electrodes was implanted for a period of two years. The device had no photovoltaic properties. The surgical procedure lasted between 3 and 4 h; however, after the surgery, all of the patients developed a combined subretinal and suprachoroidal hemorrhage.

A motion tracker was mounted on the finger and a camera facing the eye was used to monitor the gaze of the patients, and the elicited location, shape, and size of the phosphene were mapped. Although the phosphenes were reported, the threshold of the stimulation and the location varied in all of the patients.

To date, the suprachoroidal prosthesis has gained high expectations, but it has not shown good clinical results. The limitation of this suprachoroidal stimulation mainly relates to the proximity of the device to the retinal neurons. Due to its distance from the target retinal neurons, it requires higher stimulations to elicit visual percepts. Also, this geometrical gap between the device and the target neurons generates the risks of spreading the current and reducing the spatial resolution. In addition, this suprachoroidal location is known to be highly vascular, risking hemorrhage and fibrosis after implantation.

In addition to the development of microelectrode arrays and their adequate positioning near the retina for effective stimulation, the camera system that serves as the patient’s actual eye plays a crucial role in the function of the retinal prosthesis. It is usually worn on the head in the form of goggles. Although visual scanning of human individuals can be performed by a combination of head and eye movements, the eye movements have different effects for systems based on the head-mounted camera, because the source images and stimulation patterns do not change unless the camera is moved, even if the eye points in different directions. For example, patients often report spatial localization errors on a daily basis. Therefore, mounting components that process visual information from the eye (e.g., the retina and the cornea) to provide patients with nonawkward perception are considered one of the key challenges in restoring vision with retinal prosthesis. For instance, state-of-the-art subretinal prostheses use photodiodes in the subretinal region to bypass the use of head-worn goggles. Advances in SCLs that integrate visual processing units with the SCL platforms, can naturally align their position with eye movement, and also can provide synergetic effects regarding effective localization of vision by not only scanning head motions but the eye movement to mimic the natural visual system are key.

#### 7.1.3 Other Types of Retinal Prosthesis

Retinal prostheses that electrically stimulate the retinal neurons for vision restoration have been extensively explored over the past decade. However, other modalities of retinal stimulation, such as magnetic and mechanical stimulation methods, have also been reported to induce phosphenes within degenerated retinas. Although none of these methods have successfully restored vision in clinical trials, extensive research in this field has revealed promising prospects for the next-generation retinal prosthesis.^448,449^

Magnetic stimulation, fundamentally an electrical phenomenon, can be executed noninvasively without any electrical contacts. Transcranial magnetic stimulation (TMS) is a representative application of this technology. In addition to showing promise for neurological disorder treatments, it has also demonstrated potential for enhancing retinal function in Royal College of Surgeons (RCS) rats, an animal model for retinal degeneration.^450^ Phosphenes induced via direct magnetic stimulation of the retina were first observed by d’Arsonval.^451^ Lövsund et al. reported that magnetic fields could successfully evoke RGC responses in frog retinas. Shin et al. verified the feasibility of magnetic stimulation for eliciting retinal responses, using time-varying magnetic fields to generate eddy currents that stimulate the retina.^452^ Basham et al. reported the first magnetic stimulation-based epiretinal prosthesis using ferrite cores to focus the magnetic flux within a small area on the retina for localized activation..^453^ Lee et al. reported microcoils inducing different retinal responses depending on their spatial orientation, showing the potential to selectively activate different retinal neurons.^454^ A following study presented the activation of the cortical neurons and behavioral responses in mice.^455^

Mechanical stimulation of the retina has also been reported.^456^ Rountree et al. demonstrated that mechanical stimulation, based on pulsatile injections of Ames medium onto degenerated rat retinas, could elicit spatially localized retinal responses similar to those evoked by light within normal rat retinas.^457^ A conceptual optomechanical retinal prosthesis based on a high-density array of photosensitive mechanical probes was proposed to mechanically stimulate the retina by light-induced oscillation. This device was hypothesized to provide more natural vision by stimulating RGC somas without causing antidromic conduction, which can elicit irregular visual perceptions.

### 7.2 Genetic Approach: Optogenetics

**Figure 25:**
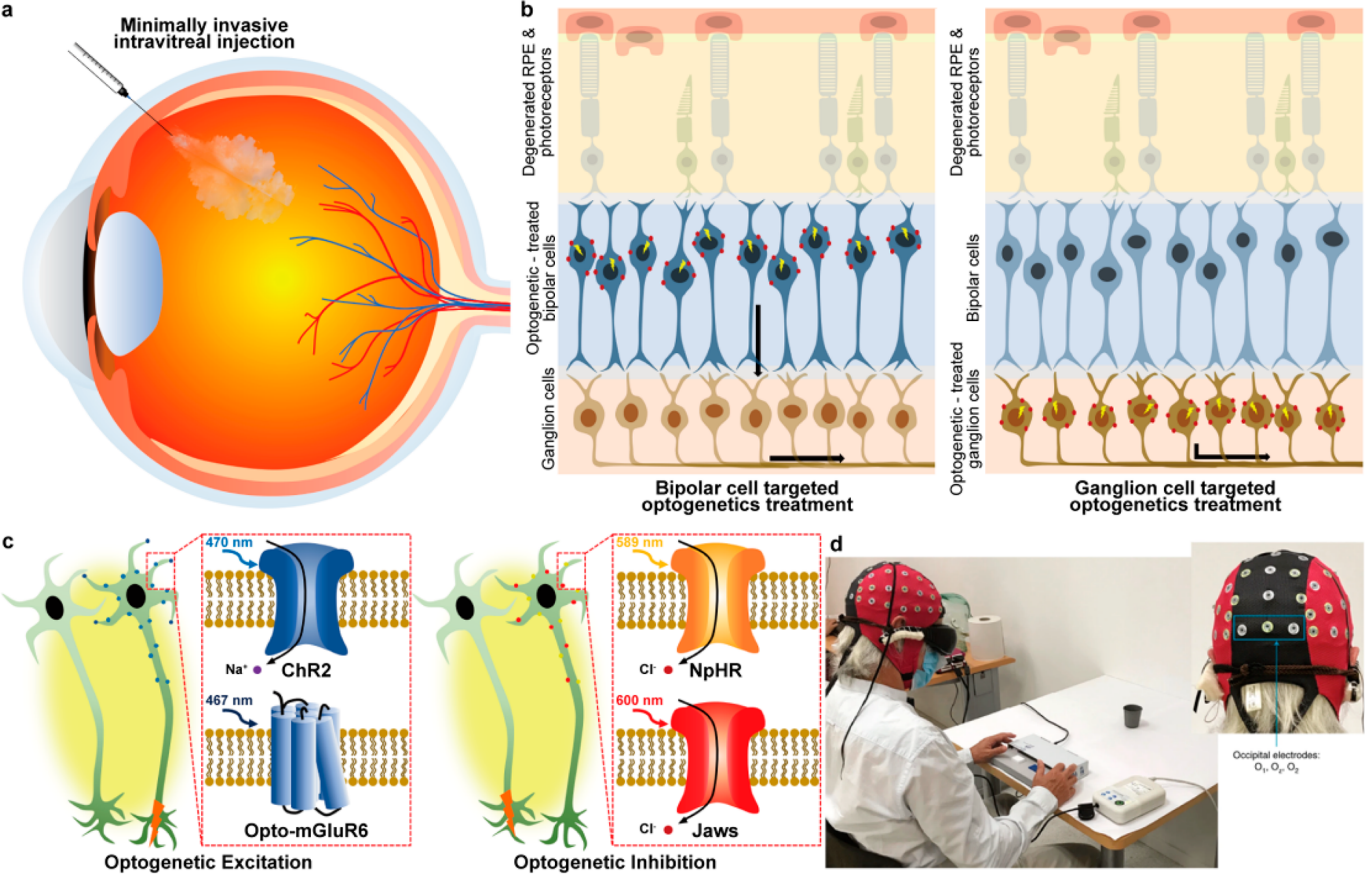
Optogenetic therapy for vision restoration. (a) Optogenetics vector delivery by intravitreal injection into vitreous cavity. (b) Electrophysiological difference in phototransduction by varying target cells. Targeting bipolar cells (left) can have advantage over RGCs (right) because of the additional transduction process from bipolar cells to RGCs. (c) Various opsins and its effect on retinal cells. ChR2 and Opto-mGluR6 cause neuronal excitation on 470 and 467 nm wavelength of light, respectively (left), while NpHR and Jaws cause neuronal inhibition on 589 and 600 nm wavelengths of light, respectively (right). (d) Experimental setup image of visual task in partial vision restoration by device supported optogenetics treatment. Behavior response was recorded in patient wearing light enhancing goggles, while occipital electrodes monitor brain activity. Reproduced with permission from ref (479). Copyright 2021 Springer Nature.

From the first demonstration of phosphene perception by electrically stimulating visual cortex in 1929 by Förster,^458^ various attempts to restore vision have been researched, which developed into retinal prosthesis. The retinal device that electrically stimulates the retina directly had made great progress, mimicking photoreceptors using photodiodes of developed resolution with smaller pixels.^459^ However, retinal prosthesis inevitably requires devices that protrude toward the adjacent region of the target cells for selective and precise stimulation, which is invasive and challenging for specific targeting. Optogenetics, in this regard, holds a great potential in vision restoration on retinas with degenerated photoreceptors, owing to the minimally invasive procedures by intravitreal injection with extremely high-selective targeting of neuronal cells in the retina (Figure 25a).

As one of the greatest breakthroughs in recent neuroscience, the field of optogenetics emerged rapidly. First optogenetics was demonstrated in mammalian neurons using microbial opsin called channelrhodopsin-2 (ChR2), and it was applied to neuroscience to unveil neural activity and synapses of the brain.^460,461^ Now, application of optogenetics is not only fixed on the brain but also has broadened to other regions of the body, especially drawing attention to the retina because of its unique mechanism of cell activation using light. Unlike external electrical stimulation, optogenetics enables the self-driven stimulation process only by light exposure to genetically modified cells. Optogenetics-treated cells undergo genetic modification by infecting the cells with adeno-associated virus (AAV), which is encoded with genes that can produce light-sensitive protein called opsins.^462^ These light-sensitive opsins first originated from unicellular algaes in wild nature, such as *Chlamydomonas*, *Volvox*, and *Natronomonas pharaonis*.^463^ Now, various opsins that originated from microbial to mammalian sources are the promising therapeutic candidate for the optogenetics tool.

Optogenetic activators can either inhibit or excite the neuronal activity of the target cell, which allows various strategies for vision restoration by targeting different kind of cells. In normal retina, phototransduction is progressed through three primary layers, transmitting the light response in the sequence of the photoreceptor cells, bipolar cells, and RGCs. Specifically, there are two types of bipolar cells, i.e., ON-bipolar cells and OFF-bipolar cells. When the bipolar cells receive excitatory neurotransmitter glutamate, ON-bipolar cells depolarize and OFF-bipolar cells hyperpolarize, which provides spatial sensitivity.^464^ The electrophysiology of the phototransduction and the morphology of the retina are the important and fundamental considerations in the therapeutic use of optogenetics for photoreceptor degenerated retinal disorders.

When photoreceptor degeneration progresses, apoptosis of the photoreceptors occurs, accompanied by phagocytosis that accelerates the degeneration.^465,466^ Optogenetics treatment can be used to overcome the degenerated photoreceptors by replacing the role to other retinal cells. Retinal cells that engage in phototransduction can be the candidate of the target cell of optogenetic treatment. However, debris of photoreceptors and RPE cells undergo clearance phagocytosis.^467^ Also, subretinal injection for gene delivery to photoreceptors or RPE cells is less favorable than intravitreal injection for RGCs or bipolar cells. For these reasons, targeting bipolar cells and RGCs is preferred in optogenetic treatment over photoreceptors (Figure 25b). Even though the photoreceptors are degenerated, it is still crucial to mimic the native visual phototransduction in optogenetics treatment because of the undergoing sophisticated visual process. Therefore, bipolar cells, middle-stream of phototransduction, are the best-known target for optogenetics. However, RGCs have the advantage over bipolar cells in terms of high ratio gene transition of the cells since targeting the layer is easier regarding its innermost location. To enhance the bipolar cell-target selectivity, promoters to improve expression have been uncovered, including Grm6 and Pcp2.^468−471^

Since the first demonstration of ChR2, various opsins have been discovered and applied in neuroscience. Both excitatory and inhibitory opsins can be used for the treatment of retinal disorder (Figure 25c). One approach for optogenetics in the retina is to excite the target RGCs or ON-bipolar cells. Bi et al. were the first to make an attempt to use optogenetics for the restoration of vision in the preclinical stage by expressing ChR2 to *rd1* mice via intravitreal injection.^472^ The expressed ChR2 was observed mainly in RGCs, but it also was observed occasionally in horizontal cells, amacrine cells, and bipolar cells, and it was most sensitive to blue light of 460 nm (although it is commonly reported that the wavelength of 470 nm is the most efficient). While various optogenetic tools target RGCs, van Wyk et al. presented Opto-mGluR6, a chimeric protein couple with melanopsin and glutamate receptor mGluR6, that targets ON-bipolar cells, which was to enhance light sensitivity by targeting the upper level of phototransduction.^473^ Melanopsin in Opto-mGluR6, which is sensitive to blue light of 467 nm, serves as a light responsive switch. While native ON-bipolar cells depolarize on light stimulation by opening transient receptor potential M1 (TRPM1) cation channel, Opto-mGluR6 responses to light with negative feedback on TRPM1 response kinetics, consequently hyperpolarizing the ON-bipolar cells. Therefore, the ON-bipolar cell with Opto-mGluR6 transmits an inversed ON/OFF light signal by negative feedback, the role of which is now replaced as OFF-bipolar cell.

The other strategy is inhibiting target cells to reactivate the retinal pathway of vision processing. Regarding the phototransduction, targeting the degenerated photoreceptor or the OFF-bipolar cell with inhibitory optogenetic tools can suggest the possibility to recuperate the retina dysfunction. *Natronomonas pharaonis* halorhodopsin (NpHR), coined after the originated algae, is the representative inhibitory optogenetic tool of neuronal activity.^474^ NpHR enables hyperpolarization of cells through light-responsive chloride pumps, which are sensitive to yellow light with the wavelength of 589 nm. Gradinaru et al. developed the opsin into enhanced NpHR (eNpHR), while Busskamp et al. performed the eNpHR on the photoreceptor cells of *rd1* mice, demonstrating the recovered retinal pathways with resensitized photoreceptors.^475,476^ Jaws, a red-shifted microbial rhodopsin with a highest sensitivity of 600 nm, is another inhibitory optogenetic tool used to target photoreceptor cone cells with increased ganglion cell spikes, which was performed from rodents to nonhuman primates.^455,477,478^ Although the use of optogenetics in OFF-bipolar cells has not been implemented yet, inhibiting the OFF-bipolar cell can suggest a possible method to recover vision. Also, potentials of optogenetic tools for OFF-bipolar cells are highly anticipated because of the synergetic performance with the simultaneous optogenetics treatment in ON-bipolar cells.

A clinical trial was performed on a blind patient to partially recover visual function, which was the first case reported in optogenetics for the restoration of vision.^479^ Diagnosed with RP 40 years ago, the patient was treated with vector injection by AAV to express ChrimsonR, a red-shifted channel rhodopsin with maximum light intensity that is around 590 nm. In addition, with the vector injection into the retina, the patient was supported with light-stimulating goggles, a device necessary for the restoration of partial vision, consisting of a camera for scene detection and a light-projector to target the eye with a beam of light. With the assistance of EEG recording, the visual signal was monitored from the occipital cortex of the brain (Figure 25d). While visual perception was unavailable when tested only with either case of optogenetics injection or goggle assistance, the patient was able to detect objects when both were performed with tasks to perceive, locate, count, and touch. Although the patient required assistive goggles with high irradiance of 13 mW cm^–2^ compared to normal daylight, it was the first research that demonstrated the neurophysiological potential of optogenetics on blind people with a highly promising result.

The main issues in optogenetics for vision restoration are the low light and temporal sensitivity. To overcome these issues, various approaches are currently being studied, such as opsin enhancement or device assistance. Current optogenetic approaches for vision restoration utilize head-worn goggles that process visual information and project the converted image onto the retina. Similar to the challenges of the head-mounted camera system for retinal prostheses, patients require special training to adapt to head movements for visual perception localization. Therefore, advances in technology integrating visual processing and optical therapeutic modalities in the SCLs can significantly facilitate the realization of natural vision, while maintaining their great potential for vision restoration in terms of minimal invasiveness and the nature-mimicking phototransduction mechanism. Therefore, optogenetics presents a new vision to blind patients with universal application for diseases related to photoreceptor degeneration, opening the new era for the restoration of vision.

In conclusion, both technologies have the potential to restore vision to the patient with degenerative retinal diseases. However, they are at different stages of development, and both have challenges that need to be addressed. Retinal prosthesis can restore visual function to patients with severe vision loss or blindness, particularly from conditions such as RP. Also, certain types of retinal prosthesis have already shown promising results in clinical trials. However, current technology only allows for low-resolution vision, and requires invasive surgical procedures for implantation, with risks of complications such as infection, retinal detachment, and inflammation. Optogenetic approaches for vision restoration are less invasive as they utilize intravitreal injection of viral vectors to deliver opsins to retinal neurons. Also, they provide high precision in controlling retinal activity with light. Still, they are largely in their early research stage, with a limited number of clinical trials to date. Also, as they require to deliver genes into target cells, they can raise potential ethical and safety issues. Therefore, future advances will likely continue to refine these techniques and expand their applicability.

## 8 Challenges and Future Outlook

### 8.1 Challenges for Practical Application

The eye has been considered as a suitable organ for diagnosis, monitoring, and treatment of diseases. In order to fabricate wearable devices for eyes, extensive research has been conducted, ranging from developments in individual elements to integration technologies. Despite these efforts to utilize the ocular devices, such as SCLs and retinal prostheses, by numerous people in daily life, several obstacles still exist that must be addressed for improved accuracy and feasibility. In this section, we discuss the challenges of wearable ocular devices to be overcome for commercialization and future perspectives as a diagnosis and treatment platform.

#### 8.1.1 Inaccurate Measurement

The eye contains various biomarkers, including chemical and physical factors for a wide range of diseases and health conditions. Some well-known substances, such as glucose, uric acid, and cholesterol, exist in relatively high concentrations in tears, and numerous sensors have been developed to measure them. However, the other substances or physical factors, which are candidates for biomarkers of specific diseases, are present in very small quantities in the eyes. In order to detect and measure minuscule biomarkers in the eyes, more accurate and sensitive sensing technologies are required. To address these limitations, the sensing materials that have high sensitivity and specificity have been investigated.^480,481^ For instance, several researchers have used new materials that they deem to be suitable for target biomarkers, such as enzymes, aptamers, and antibodies, to improve the sensing performance of electrochemical sensors. Along with the innovation in sensing technologies, machine learning and artificial intelligence technology have contributed to the utilization of SCLs as a reliable platform for diagnosing diseases and providing therapy. Pattern analysis of changes in various types of biomarkers through these technologies can enhance the feasibility of diverse biomarkers with high accuracy and sensitivity to corresponding diseases.

A method for maintaining a constant and stable collection of biomarkers also is required for reliable operation of SCLs or retinal prostheses. As one example, for SCLs, including electrochemical sensors, collecting regular amounts of tears is crucial for accurate measurement of biomarkers in the tears. The collection of tears can be affected by various factors, such as blinking, evaporation, and flow rate, which can result in fluctuations in the volumes and compositions of tears. In particular, reflex tears that occur during lens wear can make the composition of tears different. It is necessary to apply a soft material and make it thin to minimize the occurrence of reflex tear even when wearing an SCL. Therefore, if electrodes or antenna sensors can be made stretchable and properly integrated with soft materials, reflex tearing caused by wearing lenses can be reduced. In addition, tear collection may be irregular depending on the structure of the SCL or the size, position, or material of the sensor. As a result, the change in the volume or composition of tears due to the inconsistent collection of tears makes it difficult to accurately diagnose diseases. Therefore, it is necessary to consider how to collect a certain amount of tears. To address the challenge of maintaining a constant and stable collection of biomarkers in SCLs, several approaches have been proposed. One approach is to incorporate microfluidic channels or reservoirs within the lens to regulate the flow of tears and minimize evaporation. This can help maintain a consistent volume and composition of tears for analysis. Another method of collecting tears involves the use of hydrogels, which can absorb tears and swell to a certain extent. Then, the absorbed tears can be analyzed for biomarkers. However, this method has limitations in terms of the amount of tear fluid that can be collected and the duration of tear collection, as the hydrogel eventually may become saturated. Achieving a consistent and reliable acquisition method for biomarker analysis is critical for the success of SCLs as disease management platforms. Continued research and development in this area are indispensable to ensure that SCLs can effectively monitor and diagnose diseases using tear biomarkers.

#### 8.1.2 Biomarkers for the Management of Health

Previously, we described various diseases that can be diagnosed by examining one’s eyes. Numerous papers have presented devices that actualize the diagnosis of disease through the eye by measuring the glucose and cholesterol in tears. In addition, we suggested the possibility of diagnosing various metabolic diseases and neurodegenerative diseases through the measurement of various biomarkers in tears. However, the correlation between biomarkers and disease should be reconsidered and verified for higher fidelity. As mentioned earlier, there are various biomarkers that can be detected in tears and other ocular fluids. It is essential to establish the correlation between the fluctuations in these biomarkers and the presence or progression of a particular disease to assign the validity of biomarkers in the eyes. Without this correlation, the measurements of these biomarkers may not have any diagnostic or prognostic significance. Furthermore, the levels of certain biomarkers in tears are affected by a range of factors, such as age, gender, diet, and medications, making it even more challenging to discover a reliable correlation with a particular disease. Therefore, it is essential to conduct rigorous clinical studies to investigate the validity and reliability of biomarker measurements in tears and other ocular fluids for the diagnosis of disease.

In addition, it is important to consider that the specificity and sensitivity of the biomarker may be in question. A specific biomarker will only be present and affected by the presence of a particular disease, while a sensitive biomarker will be present in even small amounts in the presence of the disease, irrespective of the progression of the disease. Both specificity and sensitivity should be satisfied for the convincing usefulness of a biomarker for the diagnosis of disease. Overall, while the potential of measuring biomarkers in tears and other ocular fluids for disease diagnosis is promising, further research is necessary to establish a reliable correlation between the biomarkers and the disease, as well as to standardize the methods of measuring biomarkers.

#### 8.1.3 Wireless Operation

Wireless operation is indeed a major challenge for ocular devices, including SCLs and retinal prostheses. As mentioned above, while devices have built-in energy storage components, such as BFCs or the use of wireless communication technology to receive power from an external source, these solutions are still in the early stages of development.

One issue with built-in energy storage components is that they are miniaturized to fit within the small size of the device, leading to limited storage capacity. In addition, it can be difficult to ensure a stable energy supply, which is necessary for reliable operation.

In the case of wireless communication technology, it is free from this problem, but it should be considered that an external device for supplying power is required. Sensimed’s Triggerfish is one commercially available SCL that measures IOP and uses an embedded antenna for wireless communication. However, the device requires a bulky wireless actuation device that attaches peripherally to the eye. It also contains a heavy battery connected to it. This causes inconvenience to the user in daily life, and the external power source requires periodic charging due to its limited operating time. In addition, the Argus II system is a commercial retinal prosthesis that was first approved by the US FDA in 2013. Like Triggerfish, the device’s battery and electronics are contained within an external component that must be worn on the body, adding to the bulkiness of the system.

In order to solve this problem, research is ongoing to develop more advanced wireless communication and power supply technologies for wearable ocular devices. For example, some researchers are exploring the use of wireless power transfer systems, which could potentially provide a more convenient and reliable source of energy for wearable ocular devices. In addition, advancements in miniaturization and energy storage technology could help to address the issue of limited storage capacity in built-in energy storage components. As these technologies continue to evolve, we can expect to see improvements in the convenience, reliability, and overall performance of wearable ocular devices for disease management and health optimization.

It is important to break through the clinical barriers to solve the current challenges of contact lens technology. Ensuring the lenses are comfortable, durable, and safe for long-term wear remains a challenge. Overcoming these manufacturing hurdles is essential to make these technologies widely accessible and accepted in clinical settings. In addition, the accuracy and reliability of measurements are critical for effective disease diagnosis. Robust sensor technology, advanced algorithms for data analysis, and validation through rigorous clinical trials are necessary to address these concerns. For effective smart contact lens development, it is important to create an environment with low barriers to clinical trials through the system. Additionally, in the process of commercialization of SCLs, it will be important to prepare systems related to personal information protection and medical device regulations in advance.

#### 8.1.4 Clinical Barriers

It is important to break through the clinical barriers to solve the current challenges of contact lens technology. Ensuring the lenses are comfortable, durable, and safe for long-term wear remains a challenge. Overcoming these manufacturing hurdles is essential to make these technologies widely accessible and accepted in clinical settings. In addition, the accuracy and reliability of measurements are critical for effective disease diagnosis. Robust sensor technology, advanced algorithms for data analysis, and validation through rigorous clinical trials are necessary to address these concerns. For effective SCL development, it is important to create an environment with low barriers to clinical trials through the system. Additionally, in the process of commercialization of SCLs, it will be important to prepare systems related to personal information protection and medical device regulations in advance.

### 8.2 Future Outlook

Despite the various challenges mentioned above, innovative ocular devices, such as SCLs and retinal prostheses, are expected to rapidly establish themselves as personalized disease management platforms. Currently, SCLs primarily target only one biomarker and serve a single diagnosis or therapeutic function. However, as most diseases require complex analysis of multiple biomarkers, development in sensor technology to detect numerous biomarkers simultaneously is essential to enhance the feasibility of SCLs as an effective platform for complex analysis of various signals generated from the eye. In fact, as a multifunctional platform that can diagnose and treat simultaneously, the advancement of SCLs is actively progressing. In order for treatment through the SCLs or retinal prostheses to be applied appropriately and effectively, real-time health information that can determine the degree of treatment must be provided, and this can be achieved by a feedback system between diagnosis and treatment. Consequentially, wearable eye devices will develop into closed-loop platforms capable of complex diagnosis and treatment based on diagnosis data collected in real time, and will become powerful tools for personalized disease management.

Furthermore, the SCL is also a promising platform for implementing augmented reality (AR) similar to smart glasses. The integration of display technology in SCLs can open up a new range of possibilities for personalized disease management. AR can be implemented in SCLs to overlay digital information on top of the real-world environment. This can be particularly useful for individuals with vision impairments or those who need assistance in daily activities, such as reading, navigation, and recognizing people. The combination of AR and ocular devices can provide a more natural and intuitive interface compared to current AR devices, and provide real-time feedback by immediately monitoring health parameters without a separate device (such as a smart phone). For instance, individuals with diabetes can benefit from SCLs that can continuously monitor their glucose levels and provide real-time feedback to optimize insulin dosage. Similarly, individuals with glaucoma can receive the notifications from SCLs that continuously measure IOP and alert the wearer when the pressure reaches threshold levels. In summary, the integration of display technology in SCLs can lead to a new era of personalized disease management by providing real-time feedback, monitoring health parameters, and implementing AR technology.

## 9 Conclusion

With the popularization of wearable healthcare devices, the eye has emerged as a valuable site for disease monitoring and health management due to its complex network of physiological information and biomarkers. The use of innovative ocular devices has the potential to improve the convenience and efficiency of disease management and optimize health conditions. Especially, SCLs are promising platforms for monitoring and treating diseases in real time, while retinal prostheses can offer the possibility of restoring sight to people with certain types of blindness. However, there are still challenges to be addressed, such as sensor technology and wireless communication, to fully realize the potential of these devices. Further advances to provide multifunctional capabilities to devices can provide sophisticated, personalized, and convenient approaches to disease management. These advances in eye-based healthcare devices can offer people new opportunities to manage diseases and health conditions with sophisticated personalization, improved efficacy, and convenience.

## Abbreviations

AAV: adeno-associated virus
AD: Alzheimer’s disease
AgNFs: silver nanofibers
AgNW: silver nanowire
AH: aqueous humor
AMD: age-related macular degeneration
ApoB: apolipoprotein B
BAB: blood-aqueous barrier
BDNF: brain-driven neurotrophic factor
BFC: biofuel cell
BRB: blood-retinal barrier
CE: counter electrode
CGM: continuous glucose monitoring
cGMP: cyclic guanosine monophosphate
ChR2: channelrhodopsin-2
CLC: cholesteric liquid crystal
CNS: central nervous system
CVD: cardiovascular disease
DC: direct current
DDS: drug delivery systems
DME: Diabetic macular edema
EEG: electroencephalography
EFCs: enzymatic fuel cells
EHD: engineering electrohydrodynamic engineering
ERG: electroretinogram
FAF: fundus autofluorescence
FET: field-effect transistor
FPG: fasting plasma glucose
GMP: guanosine monophosphate
GOx: glucose oxidase
IL-6: interleukin-6
INL: inner nuclear layer
IOP: intraocular pressure
IPL: inner plexiform layer
LDL-C: lipoprotein cholesterol
LEDs: Light-emitting diodes
Lox: lactate oxidase
MFCs: microbial fuel cells
mGluR6: metabotropic glutamate receptor
MMP-9: matrix metalloproteinase-9
MS: multiple sclerosis
MSU: monosodium urate
NFC: near field communication
NGF: nerve growth factor
NpHR: Natronomonas pharaonis alorhodopsin
NTG: normal tension glaucoma
OCT: optical coherence tomography
OGTT: oral glucose tolerance test
OMS: optical monitoring system
ONL: outer nuclear layer
OPL: outer plexiform layer
OS: outer segment
OSI: surface inflammation
OST: ocular surface temperature
PACG: primary angle-closure glaucoma
PD: Parkinson’s disease
PDMS: polydimethylsiloxane
POAG: primary open-angle glaucoma
RE: reference electrode
RF: radio frequency
RFID: Radio-Frequency Identification
RGCs: retinal ganglion cells
RLC: resistor-inductor-capacitor
ROS: reactive oxygen species
RP: retinitis pigmentosa
RPE: retinal pigment epithelium
SCL: smart contact lens
T1D: Type 1 diabetes
T2D: Type 2 diabetes
TC: total cholesterol
TGs: triglycerides
TLRs: Toll-like receptors
TNF-α: tumor necrosis factor-alpha
TRPM1: transient receptor potential M1
VH: vitreous humor
WDs: wearable devices
WE: working electrode
WPT: wireless power transmission
